# Differential abilities to engage inaccessible chromatin diversify vertebrate Hox binding patterns

**DOI:** 10.1242/dev.194761

**Published:** 2020-11-23

**Authors:** Milica Bulajić, Divyanshi Srivastava, Jeremy S. Dasen, Hynek Wichterle, Shaun Mahony, Esteban O. Mazzoni

**Affiliations:** 1 Department of Biology, New York University, New York, NY 10003, USA; 2 Center for Eukaryotic Gene Regulation, Department of Biochemistry and Molecular Biology, The Pennsylvania State University, University Park, PA 16802, USA; 3 Neuroscience Institute, Department of Neuroscience and Physiology, New York University School of Medicine, New York, NY 10016, USA; 4 Department of Pathology and Cell Biology, Columbia University Irving Medical Center, New York, NY 10032, USA; 5 Department of Neuroscience, Columbia University Irving Medical Center, New York, NY 10032, USA

**Keywords:** Hox, Binding, Chromatin, Spinal cord, Stem cell differentiation, Patterning

## Abstract

Although Hox genes encode for conserved transcription factors (TFs), they are further divided into anterior, central and posterior groups based on their DNA-binding domain similarity. The posterior Hox group expanded in the deuterostome clade and patterns caudal and distal structures. We aimed to address how similar Hox TFs diverge to induce different positional identities. We studied Hox TF DNA-binding and regulatory activity during an *in vitro* motor neuron differentiation system that recapitulates embryonic development. We found diversity in the genomic binding profiles of different Hox TFs, even among the posterior group paralogs that share similar DNA-binding domains. These differences in genomic binding were explained by differing abilities to bind to previously inaccessible sites. For example, the posterior group HOXC9 had a greater ability to bind occluded sites than the posterior HOXC10, producing different binding patterns and driving differential gene expression programs. From these results, we propose that the differential abilities of posterior Hox TFs to bind to previously inaccessible chromatin drive patterning diversification.

This article has an associated ‘The people behind the papers’ interview.

## Abstract (precis)

**Highlighted Article:** Analyses of Hox transcription factor (TF) DNA-binding and regulatory activity demonstrates that the ability of mammalian posterior Hox TFs to bind to previously inaccessible chromatin is the predominant force driving their patterning diversification.

## INTRODUCTION

Hox genes encode a highly conserved transcription factor (TF) family that endows cells with positional identity during embryonic development (McGinnis and Krumlauf, 1992; Lewis, 1978; Duboule and Dolle, 1989). In mammals, Hox genes are organized into four clusters located on different chromosomes (*HoxA*, *HoxB*, *HoxC* and *HoxD*). Each cluster contains a subset of 13 similar paralogous Hox genes, genomically arranged in the same linear order as their spatial and temporal expression patterns in the developing embryo, a phenomenon known as collinearity (Kmita and Duboule, 2003; Duboule and Morata, 1994). Changes in Hox gene expression patterns induce gross morphological changes, resulting in well-characterized homeotic transformations. However, how Hox TFs assign different positional identities during cell differentiation is not entirely understood.

Hox genes encode for similar homeodomain (HD)-containing TFs (Akam, 1989; Regulski et al., 1985). HDs are highly conserved helix-turn-helix DNA-binding domains that recognize similar consensus DNA sequences (Gehring et al., 1994; Noyes et al., 2008; Affolter et al., 2008; Berger et al., 2008). Vertebrate Hox TFs are divided into anterior (HOX1-5), central (HOX6-8) and posterior (HOX9-13) paralog groups. The posterior Hox group is particularly interesting because it has expanded in the deuterostome clade. In *Drosophila* there is only one *Abd-B*, whereas several *Abd-B*-related Hox genes assign different posterior positional identities during vertebrate development (Izpisúa-Belmonte et al., 1991) (reviewed by Duboule, 2007; Lanfear, 2010). Thus, understanding how paralogous Hox TFs differentiate their genomic binding activity to specify cell fates is at the core of understanding vertebrate body patterning.

*In vitro* binding studies have investigated the intrinsic sequence preferences of Hox TFs alone or in complex with specific co-factors. These studies demonstrate that the anterior (HOX1-5) and central Hox paralog groups (HOX6-8) prefer to bind the canonical TAAT core sequence, whereas the posterior paralog groups (HOX9-13) preferentially bind TTAT core sequences (Noyes et al., 2008; Mann et al., 2009; Berger et al., 2008; Ekker et al., 1994). Moreover, the interaction between Hox TFs and MEIS and PBX co-factors increases the specificity and selectivity of Hox DNA binding (reviewed by Mann and Chan, 1996; Merabet and Mann, 2016; Mann and Affolter, 1998). However, how vertebrate Hox TFs within a single group diversify their genomic binding patterns remains obscure.

Despite the extensive analysis of Hox TF binding *in vitro*, relatively little is known about Hox binding specificity in the context of cellular chromatin landscapes (De Kumar et al., 2017; Huang et al., 2012; Donaldson et al., 2012). For example, virally expressed HOXA9-13 and HOXD9-13 in primary chicken mesenchymal limb progenitors exhibit some binding specificity differences between the posterior group Hox TFs, with HOXA/D13 paralogs being the most different (Jerković et al., 2017). In *Drosophila*, recent studies have investigated the role of chromatin accessibility in shaping Hox TF binding. Of the central and posterior fly Hox factors, *Drosophila* Abd-B displays an increased ability to bind previously inaccessible chromatin (Beh et al., 2016; Porcelli et al., 2019). However, it is not possible to investigate how binding selectivity has diverged between the vertebrate Abd-B-derived posterior Hox TFs (HOX9-HOX13) using *Drosophila* models. In line with their differential patterning activities, the vertebrate Hox TFs might have diverged in their sequence preferences or abilities to engage inaccessible chromatin.

In vertebrates, Hox genes pattern various developing tissues. Notably, spinal cord neuronal diversity requires Hox gene activity along its rostrocaudal axis (Sweeney et al., 2018; Dasen and Jessell, 2009; Dasen et al., 2003). The limb-innervating expression program is controlled by central Hox TFs (HOX6 and HOX8) at the brachial spinal cord and by posterior Hox TFs (HOX10) at the lumbar spinal cord (Dasen et al., 2008; Jung et al., 2018; Lacombe et al., 2013; Rousso et al., 2008; Wu et al., 2008). Thus, a similar neuron fate is induced by Hox TFs with different DNA sequence preferences. Meanwhile, the posterior HOXC9 induces thoracic fate (Jung et al., 2010,^2014^). Thus, two posterior group genes, *Hoxc9* and *Hoxc10*, induce different spinal cord fates. In agreement with their genomic cluster position, *Hox13* paralogs are expressed late during development, distally and in posterior regions. They are associated with patterning structures derived from the caudal tail bud by inhibiting cell growth or inducing apoptosis (Economides et al., 2003; Godwin and Capecchi, 1998; Denans et al., 2015; Young et al., 2009). As a model to understand the differential patterning activities of Hox TFs, we sought to understand how central and posterior Hox TFs bind the genome to induce different spinal cord identities.

The study of genome-wide Hox TF binding in cellular contexts is challenging due to the lack of availability of homogenous relevant cell populations at scales compatible with chromatin immunoprecipitation (ChIP). To mitigate this technical limitation, we opted for an embryonic stem cell (ESC) differentiation system that recapitulates ventral spinal cord early development (Wichterle et al., 2002; Davis-Dusenbery et al., 2014; Wichterle and Peljto, 2008). In response to the dorsoventral Hedgehog and rostrocaudal retinoic acid (RA) patterning signals, ESCs differentiate into motor neurons (MNs) and interneurons by transitioning through progenitor states (Wichterle et al., 2002). The culture acquires a rostral spinal cord identity, with 90% of cells expressing *Hoxa5* (Peljto et al., 2010; Peljto and Wichterle, 2011). Moreover, differentiating ESC-derived MNs respond to Hox gene overexpression similarly to those in the developing spinal cord (Narendra et al., 2016; Tan et al., 2016; Machado et al., 2014; Jung et al., 2010). Thus, ESC-to-MN differentiation recapitulates crucial aspects of MN differentiation and constitutes a suitable model to study Hox TF activity in relevant cellular and chromatin environments.

To understand how Hox TFs control different fates, we induced individual Hox TFs in progenitor MNs. We analyzed the differential binding of the Hox TFs in the context of their underlying sequence motifs and interactions with the pre-existing chromatin accessibility environment. We focused on a subset of HOXC TFs (HOXC6, 8, 9 and 10) due to their importance in inducing spinal cord identities. We complemented these studies by examining additional HOX9 paralogs and the posterior HOXC13. Our results suggest that limb-innervating fate is not the product of identical central and posterior Hox binding patterns, as HOXC10 does not mimic HOXC6 and HOXC8 binding profiles. Although posterior group Hox TFs have similar DNA motif preferences, they do not bind to the genome with identical patterns. This difference is mainly due to their differing abilities to bind motifs occluded in inaccessible chromatin. For example, HOXC9 and HOXC10 bind similar DNA sequence motifs, whereas HOXC9 has a greater ability to bind previously inaccessible chromatin. In summary, our work describes divergence in the abilities to engage inaccessible chromatin among vertebrate posterior group Hox factors derived from a single *Drosophila* gene. From these results, we propose that the differential abilities of posterior Hox TFs to bind to previously inaccessible chromatin is the predominant force driving their patterning diversification.

## RESULTS

### Hox TF expression controls neuronal fates during *in vitro* spinal cord differentiation

**Fig. 1.**
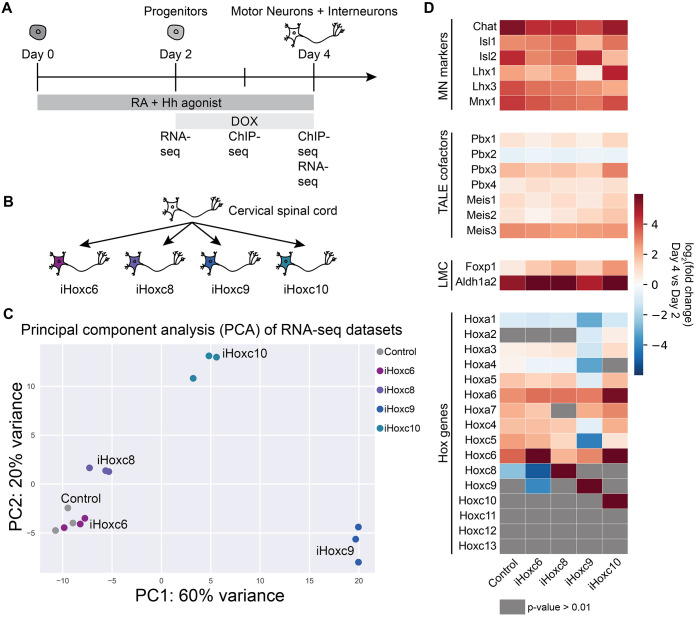
**Hox TFs control cell fates during *in vitro* spinal cord differentiation.** (A) Overview of the experimental procedure. ESCs differentiate into MNs and interneurons in response to RA and Hedgehog patterning signals. Hox expression was induced by treating cells with Dox. Cells were collected at distinct time points for RNA-seq and ChIP-seq for Hox TFs. (B) ESC-derived MNs differentiated with RA and Hedgehog acquire a rostral cervical spinal cord fate. They respond to Hox gene overexpression by inducing distinct transcriptional profiles, evident in Fig. 1C and Fig. S1C. (C) PCA of the RNA-seq datasets (day 4) reveals similarities in the gene expression profiles induced by Hox TFs (each dot represents independent differentiations). (D) RNA-seq heatmap showing the expression of representative marker genes in no Dox control, iHoxc6, iHoxc8, iHoxc9 and iHoxc10 neurons relative to day 2 progenitors (*n*=3 independent differentiations).

Hox proteins have similar DNA-binding domains, yet they control positional identity along the rostrocaudal axis. In particular, posterior HOXC9 and HOXC10 have a single shared *Drosophila* ortholog, Abd-B, yet they pattern different spinal cord fates. We focused our attention on a subset of *HoxC* genes due to their cardinal spinal cord patterning activities (Dasen and Jessell, 2009; Dasen et al., 2003; Jung et al., 2010; Wu et al., 2008). We generated isogenic mouse ESC lines that expressed *Hoxc6* (brachial), *Hoxc8* (brachial), *Hoxc9* (thoracic) or *Hoxc10* (lumbar) upon doxycycline (Dox) treatment (Fig. 1A,B, Fig. S1A,B) (Mazzoni et al., 2011; Iacovino et al., 2011).

As time progresses, cells become neurons, peaking at 48 h after Dox addition when the culture mostly consists of postmitotic MNs as well as interneurons. We refer to these as iHoxc6, iHoxc8, iHoxc9 and iHoxc10 neurons. Importantly, all Hox proteins are N-terminally Flag-tagged, which allows for immunoprecipitation with the same antibody, eliminating any bias that could occur from different antibody affinities.

HOXC6, HOXC9 and HOXC10 divide the spinal cord into three important levels: brachial, thoracic and lumbar, respectively. *Hoxc6*, *Hoxc8* and *Hoxc9* inductions during *in vitro* MN differentiation have been individually characterized with expression changes in a few downstream genes, and they produce the expected phenotypes (Jung et al., 2010; Narendra et al., 2016; Tan et al., 2016). To have a comprehensive and comparative integration of Hox expression consequences, we performed RNA-seq in *Hoxc6*-, *Hoxc8*-, *Hoxc9*- and *Hoxc10*-induced postmitotic neurons, as well as control neurons not treated with Dox (Fig. 1A, Fig. S1A). In a principal component analysis (PCA), the first two principal components explained 80% of the variance in RNA-seq tag counts, reflecting a combination of the paralog group and the subtype identities specified by the Hox proteins (Fig. 1C). Control cells expressed Hox genes up to paralogs *Hox5*. Thus, iHoxc6 induced some expression changes grouping close to control (Fig. 1C). The slightly more posterior inducing Hox, iHoxc8, separated along PC2. iHoxc9 grouped the furthest away, whereas iHoxc10 grouped between cells expressing *Hox5-8* genes (control, iHoxc6 and iHoxc8) and iHoxc9. Multidimensional scaling (MDS), a non-linear dimensionality reduction technique, produced a similar lower-dimensional embedding separating each inducible Hox line (Fig. S1C). Overall, these data show that Hox TFs induce distinct gene expression profiles during *in vitro* spinal cord differentiation, with an increasing transcriptional diversity by posterior Hox TFs (Fig. 1C, Fig. S1C,D).

Although there is no global gene expression characterization of Hox expression manipulation during embryonic development, iHox lines induced the expression of marker genes in agreement with previous studies. For example, *Hoxc6*, *Hoxc8* and *Hoxc10* overexpression induced canonical lateral motor column (LMC) markers *Raldh2* and *FoxP1* (Fig. 1D, Fig. S2A,B) (Narendra et al., 2016; Tan et al., 2016). *Hoxc9* overexpression led to the repression of anterior *Hox1-5* paralogs (Fig. 1D, Fig. S2A,B) (Jung et al., 2010). The various induced Hox genes displayed similar RNA levels, which are also comparable to those of endogenous *Hoxc5* expressed in the control (Fig. S2C). Importantly, Hox induction during *in vitro* spinal cord differentiation did not derail the ability of cells to acquire an MN identity (Fig. 1D, Fig. S2A,B). Thus, Hox TF activity during ESC differentiation induces distinct spinal cord fates, recapitulating aspects of embryonic differentiation.

### HOXC6, HOXC9 and HOXC10 TFs have different genome-wide binding profiles

To understand how Hox TFs assign positional identity, we assayed the genomic binding of HOXC6, HOXC8, HOXC9 and HOXC10 by performing ChIP-seq experiments 24 h and 48 h after Dox treatment, with newborn and young postmitotic MNs, respectively (Fig. 1A, Fig. S1A). These time points are crucial for Hox positional identity patterning because Hox TFs control MN types at early postmitotic states (Dasen et al., 2003). We restricted the analysis to the top 10,000 sites in each dataset for all downstream analyses to ensure the least amount of bias from comparing different experiments and performed differential binding analysis using MultiGPS (Mahony et al., 2014), which runs edgeR (Robinson et al., 2010) internally.

**Fig. 2.**
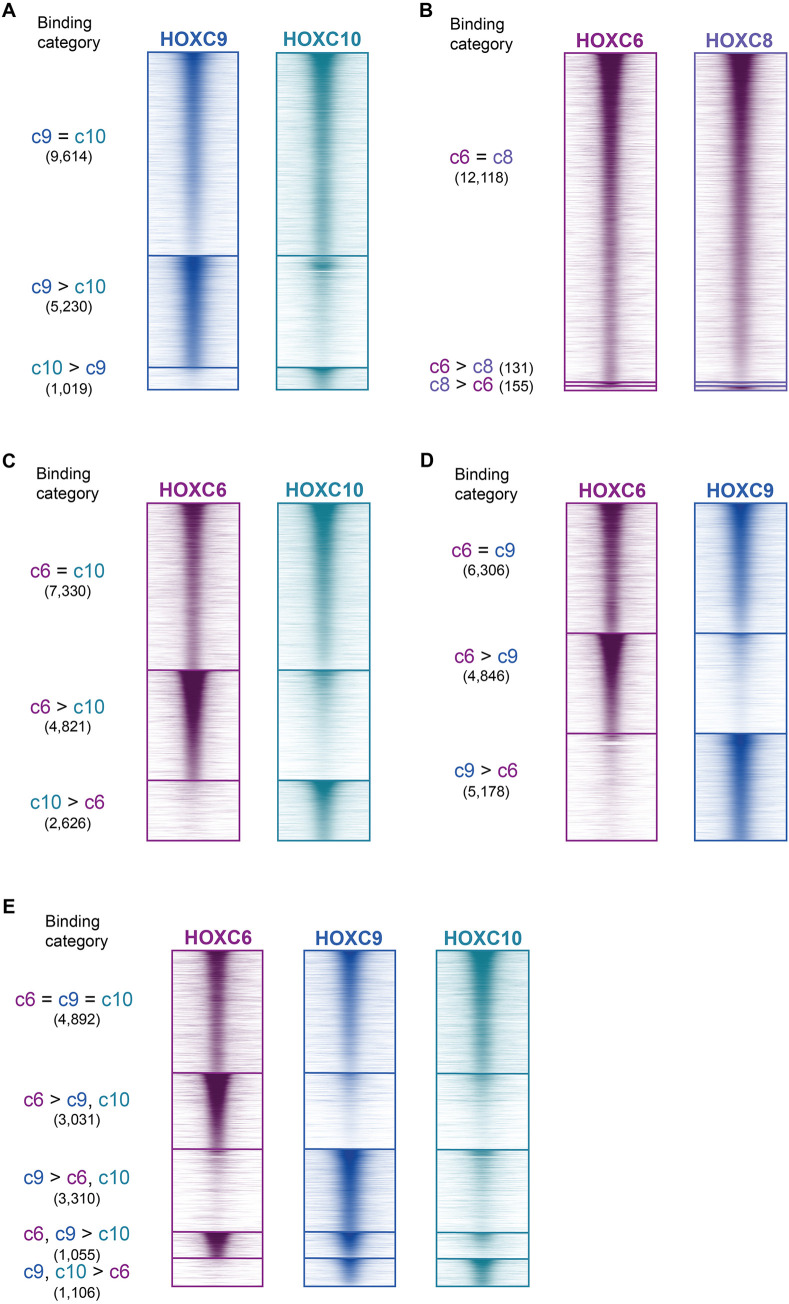
**HOXC6, HOXC9** **and HOXC10 TFs have different genome-wide binding profiles.** (A-D) ChIP-seq heatmap showing binding comparisons of Hox TFs in differentiating neurons at day 3 (*n*=2 independent differentiations). Sites bound by both indicated Hox TFs noted as ‘=’ sites. Preferentially bound sites by HOXC6, HOXC8, HOXC9 or HOXC10 noted as ‘c6 >’, ‘c8 >’, ‘c9 >’ and ‘c10 >’, respectively. (E) ChIP-seq heatmap showing binding comparisons of HOXC6, HOXC9 and HOXC10 in differentiating neurons at day 3 (*n*=2 independent differentiations). Sites bound by all three Hox TFs are indicated as ‘c6=c9=c10’ sites. Preferentially bound sites by HOXC6, HOXC9, HOXC6 and HOXC9, or HOXC9 and HOXC10 are indicated as ‘c6>c9, c10’, ‘c9>c6, c10’, ‘c6, c9>c10’, and ‘c9, c10>c6’, respectively.

Despite sharing 82% similarity within their HDs (Fig. S3A), we found that the posterior HOXC9 and HOXC10 displayed divergent genomic binding patterns (Fig. 2A, Fig. S3B). HOXC10 primarily bound a subset of HOXC9 binding sites; while 90% of the top 10,000 binding sites for HOXC10 showed similar enrichment levels for HOXC9 and HOXC10, an additional 5230 sites were bound preferentially by HOXC9 (Fig. 2A). Thus, although HOXC9 and HOXC10 contain similar DNA-binding domains, HOXC9 binds to additional genomic locations.

We wondered if another pair of similar Hox TFs would also diverge in binding patterns. Thus, we compared the binding of central HOXC6 and HOXC8 TFs. HOXC6 and HOXC8 displayed few differences in enrichment at their top-most bound sites; fewer than 2% of sites were significantly differentially bound by the two TFs (Fig. 2B). Thus, unlike the posterior HOXC9 and HOXC10, the two main brachial central group Hox proteins bind similar target sites in differentiating neurons. To facilitate the interpretation of the subsequent comparisons, we picked the canonical HOXC6 to represent the brachial-inducing Hox binding profile.

Next, we compared the binding patterns of the central HOXC6 TF versus each of the posterior Hox TFs, HOXC9 and HOXC10. Although both induce limb-innervating spinal cord fate, HOXC6 and HOXC10 do not have identical binding patterns. Although there are sites that HOXC6 and HOXC10 bind at similar levels, there are also unique HOXC6 and HOXC10 sites in differentiating cells (Fig. 2C, Fig. S3C). Similarly, HOXC6 and HOXC9 bound to some sites at similar levels but there were also sites differentially bound by HOXC6 or HOXC9 (Fig. 2D, Fig. S3D). Thus, the different patterning abilities of central and posterior Hox proteins might be explained in part by differential genome-wide binding profiles.

To better characterize the diversity of sites bound by the various Hox TFs, we performed a joint differential binding analysis for HOXC6, HOXC9 and HOXC10 (Fig. 2E, Fig. S3E). This analysis revealed that 4892 sites were bound similarly by HOXC6, HOXC9 and HOXC10 (‘c6=c9=c10’) (Fig. 2E). HOXC6 and HOXC9 differentially bound large sets of private sites: 3031 sites were bound by HOXC6 (‘c6>c9,c10’) and 3310 sites were bound by HOXC9 (‘c9>c6,c10’). There were 1106 sites preferentially bound by posterior Hox TFs (‘c9,c10>c6’). Finally, there were 1055 sites bound by HOXC6 and HOXC9 (‘c6,c9>c10’). Of note, Hox binding sites were overwhelmingly distal to transcriptional start sites (Fig. S4A), and Hox TF binding patterns were mostly the same in newborn and young postmitotic MNs (Fig. S4B-D).

Altogether, we found a set of sites that are bound by all assayed Hox TFs, regardless of paralog group or fate inducing activity. The brachial HOXC6 and thoracic HOXC9 bind additional sets of unique sites. Finally, although the posterior HOXC10 and HOXC9 are predicted to share sequence specificity, HOXC10 cannot bind to a large fraction of HOXC9 sites.

### Sequence preference does not explain posterior HOXC9 and HOXC10 binding differences

**Fig. 3.**
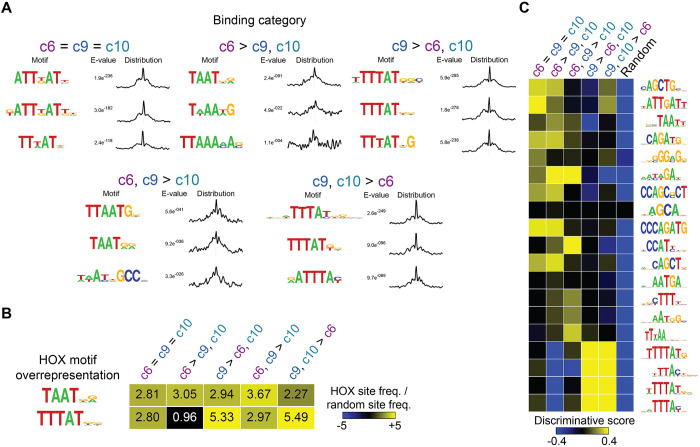
**Sequence preference does not explain posterior HOXC9 and HOXC10 binding differences.** (A) Selected top enriched motifs discovered via MEME-ChIP at the indicated Hox binding categories featured in Fig. 2E. Distributions to the right of each motif show the distribution of each motif occurrence with respect to the midpoint of each peak (500 bp windows). (B) Hox TF anterior and posterior motif over-representation (compared with randomly selected sequences) at each category of Hox binding sites. (C) SeqUnwinder analysis characterizing motifs that are discriminative between the various classes of HOXC6, HOXC9 and HOXC10 binding sites.

Next, we sought to investigate whether distinct sequence preferences define the differential HOXC9 and HOXC10 binding patterns. Previous *in vitro* binding preference studies reported that anterior and central Hox paralogs prefer the TAAT core motif, whereas the posterior paralogs prefer the TTAT motif (Noyes et al., 2008; Mann et al., 2009). Indeed, *de novo* motif discovery, using the ensemble method MEME-ChIP (Machanick and Bailey, 2011), revealed distinct motifs when comparing sites bound by central versus posterior Hox TFs during MN differentiation (Fig. 3A). The representative enriched motifs detected at sites bound by HOXC6 contained the TAAT sequence. In contrast, the identified sequences at sites bound by HOXC9 and HOXC10 matched the posterior motif TTTAT, and the bipartite PBX (TALE co-factor) and posterior Hox motif TGATTTAT at c6=c9=c10 sites (Fig. 3A). Thus, central and posterior Hox proteins bind to motifs that are in agreement with previous *in vitro* binding preference studies (Noyes et al., 2008; Mann et al., 2009). However, both c9>c6,c10 and c9,c10>c6 binding categories have similar detected TTTAT motifs, failing to discriminate sequence preference within the posterior group. We next used motif scanning approaches to directly compare the over-representation of each type of Hox motif. These results were consistent with the previous motif-finding results and show similar over-representation levels of the TTTAT motif at sites bound by HOXC9 versus HOXC9=HOXC10 (Fig. 3B).

Finally, we used a multi-class discriminative *k*-mer based motif-finder (SeqUnwinder; Kakumanu et al., 2017) to find motifs that discriminate between each subset of Hox binding sites (Fig. 3C). We found both cognate Hox TAAT motifs and additional secondary motifs that discriminate sites bound by the central HOXC6 from sites bound by posterior Hox TFs. However, notably, SeqUnwinder discovered no motifs that could discriminate between sites bound by HOXC9 alone versus those bound by HOXC9 and HOXC10. Thus, we see no evidence for sequence preference differences that could explain the differential binding observed between HOXC9 and HOXC10.

### HOXC9 has a higher preference for relatively inaccessible chromatin than HOXC6 and HOXC10

In addition to the sequence preferences of a TF, cell type-specific chromatin environments can specify genome-wide TF binding specificity. As we found no strong evidence that sequence preference explains HOXC9 versus HOXC10 differences, we decided to explore whether binding to previously inaccessible sites shapes their binding patterns.

**Fig. 4.**
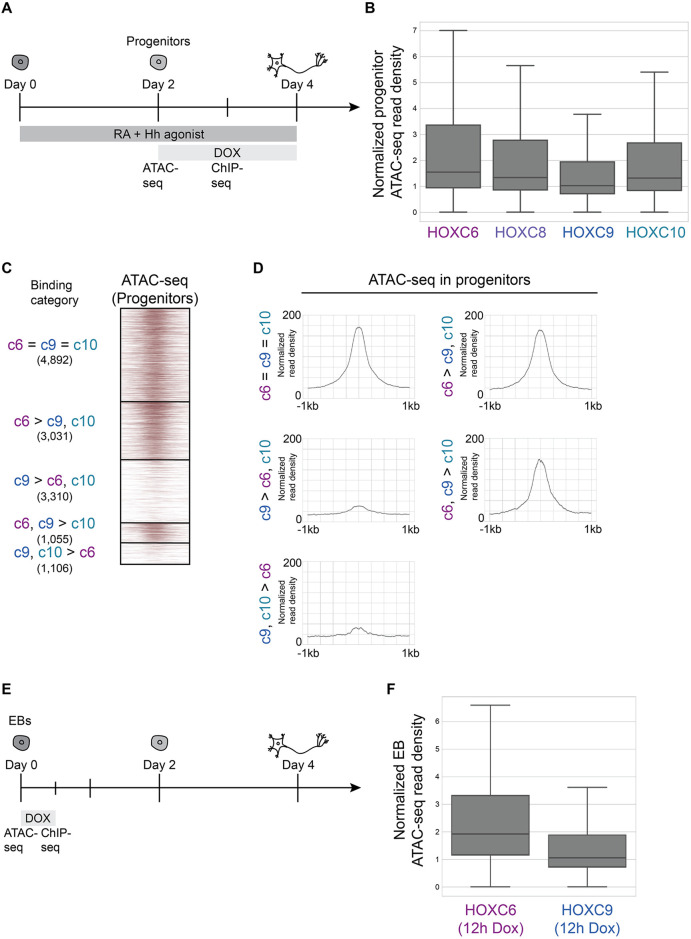
**HOXC9 has a higher preference for inaccessible chromatin than HOXC6 and HOXC10.** (A) Overview of the experimental procedure. ESCs differentiate into MNs and interneurons in response to RA and Hedgehog patterning signals. Hox expression was induced by treating cells with Dox. Cells were collected at distinct time points for ATAC-seq and ChIP-seq. (B) The distribution of day 2 progenitor ATAC-seq read density at the top 10,000 HOXC6, HOXC8, HOXC9 and HOXC10 sites at day 3 (*n*=2 independent differentiations). Data are ordered based on normalized read density (tags per million per site) and divided into quartiles. (C) ATAC-seq heatmap displaying the accessibility in day 2 progenitors (before Hox induction) at the indicated Hox binding categories from Fig. 2E. (D) Metagene plots of accessibility in progenitors displaying the prior accessibility (before Hox induction) at the indicated binding category. Normalized read density represents tags per million per 1000 sites. (E) Overview of the experimental procedure for the results in Fig. 4F. Hox expression was induced by treating unpatterned EBs with Dox. Cells were collected at distinct time points for ATAC-seq and ChIP-seq. (F) The distribution of EB ATAC-seq read density at the top 10,000 HOXC6 and HOXC9 sites in EBs (12 h Dox induction; *n*=2 independent differentiations). Data are ordered based on normalized read density (tags per million per site) and divided into quartiles. Boxes display the central 50% (quartile 2 and quartile 3), and the top and bottom whiskers represent the top 25% and bottom 25% (the top and bottom quartiles) of the data, respectively.

To investigate whether the chromatin accessibility landscape that exists before Hox induction shapes Hox TF binding patterns, we characterized genome-wide chromatin accessibility states by ATAC-seq at the progenitor stage (Fig. 4A, Fig. S1A). The distribution of progenitor ATAC-seq read density at HOXC6, HOXC8, HOXC9 and HOXC10 sites revealed that HOXC9 binding sites had the lowest median accessibility before each factor is induced (Fig. 4B). We then analyzed prior accessibility differences within the different established Hox binding categories (Fig. 2E). In agreement, all sites with HOXC6 binding (c6=c9=c10, c6>c9,c10 and c6,c9>c10 categories) harbored similar prior accessibility landscapes (55%, 54% and 45% of sites overlap accessible domains, respectively) (Fig. 4C,D). On the other hand, c9>c6,c10 and c9,c10>c6 binding occurred in genomic locations with much lower prior accessibility (16% and 18% of sites overlap accessible domains, respectively) (Fig. 4C,D). Interestingly, the sites with higher prior accessibility landscapes were associated with non-Hox motifs; for example, bHLH factors that are common during neuronal differentiation (Fig. 3C).

To test whether Hox differential preferences for accessible regions were Hox TF intrinsic or due to a progenitor-specific chromatin and co-factor environment, we investigated the binding of HOXC6 and HOXC9 TFs in undifferentiated cells (Fig. 4E). Even in this different cell type, HOXC9 maintained a higher preference for inaccessible chromatin than HOXC6 (Fig. 4F). Thus, the intrinsic ability of Hox TFs to bind to inaccessible chromatin seems to be independent of the particular cellular environment in which they are expressed.

Altogether, comparing Hox TF binding with prior accessibility revealed that limb-innervating HOXC10 and HOXC6 rely more on chromatin accessibility established at progenitor stages to find their target sites. Moreover, these results divide the posterior paralog group by their ability to bind previously inaccessible chromatin, with HOXC9 displaying a greater ability to bind inaccessible chromatin compared to HOXC10.

### HOX TF binding increases chromatin accessibility

**Fig. 5.**
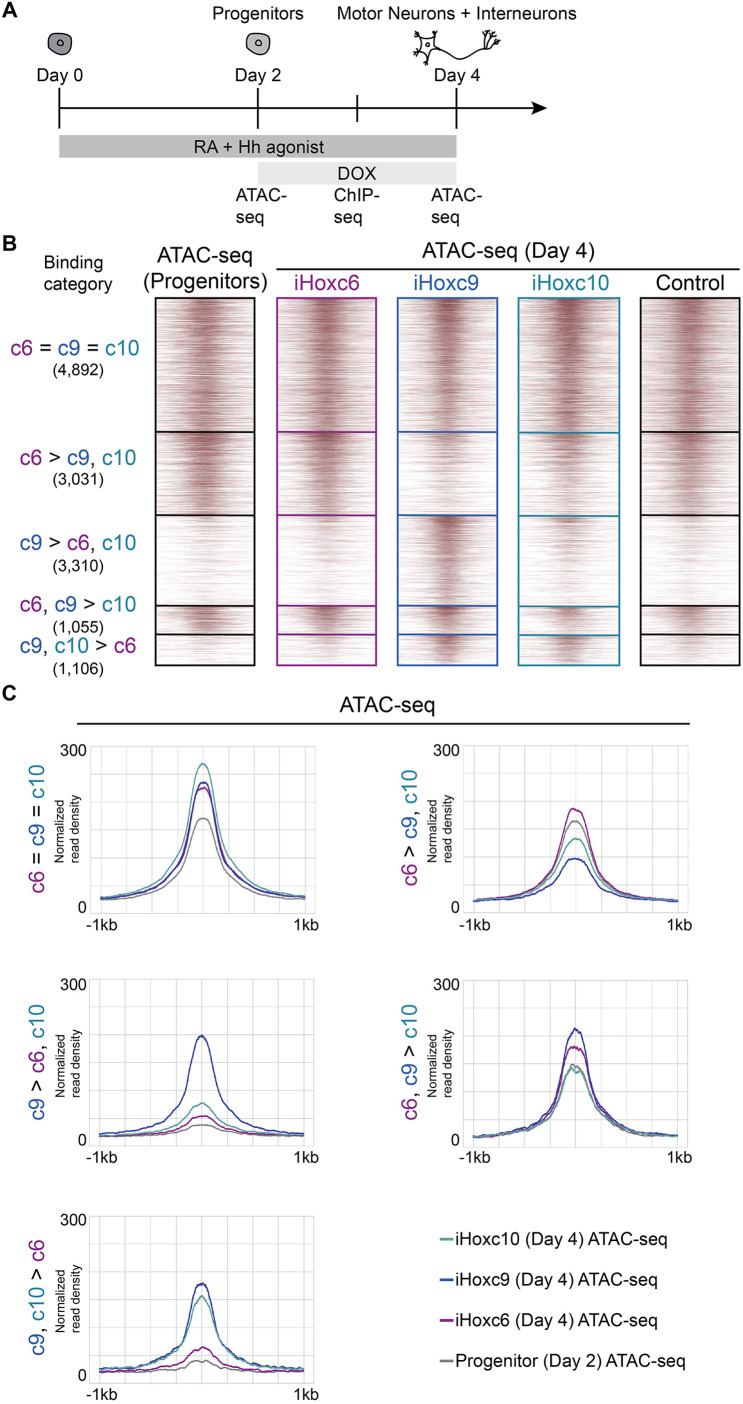
**HOX TF binding increases chromatin accessibility.** (A) Overview of the experimental procedure. ESCs differentiate into MNs and interneurons in response to RA and Hedgehog patterning signals. Hox expression was induced by treating cells with Dox. Cells were collected at distinct time points for ATAC-seq and ChIP-seq. (B) ATAC-seq heatmaps displaying the accessibility in day 2 progenitors versus iHoxc6 versus iHoxc9 versus iHoxc10 versus No Dox control neurons, at the indicated binding categories from Fig. 2E (*n*=2 independent differentiations). (C) Metagene plots showing the accessibility gain in iHoxc6 versus iHoxc9 versus iHoxc10 neurons at the indicated binding category. Normalized read density represents tags per million per 1000 sites.

The difference in the abilities of Hox TFs to bind to inaccessible chromatin prompted us to investigate whether Hox TFs change the accessibility landscape after binding. Would the ability of HOXC9 to bind inaccessible sites be coupled with increasing accessibility after binding? To characterize the accessibility changes after Hox binding, we compared the accessibility status of progenitors and postmitotic neurons at Hox binding events (Fig. 5A, Fig. S1A).

We found that sites bound by HOXC9 and HOXC10 (c9,c10>c6) gained accessibility in both iHoxc9 and iHoxc10 but did not significantly change in iHoxc6 neurons (Fig. 5B,C). Strikingly, exclusive HOXC9 sites increased accessibility the most and only in response to *Hoxc9* expression (Fig. 5B,C). Consistent with Hox TFs maintaining the pre-existing chromatin status, c6=c9=c10 sites gained some accessibility after Hox binding (Fig. 5B,C). Accordingly, c6>c9,c10 sites gained some accessibility in iHoxc6 neurons, whereas they lost accessibility in iHoxc9- and iHoxc10-expressing cells.

The dynamic accessibility changes after Hox binding revealed that not all Hox TFs have the same ability to modify chromatin accessibility. Among those we analyzed, HOXC9 stands out in its ability to bind to a large set of sites in relatively inaccessible chromatin and increase the accessibility status after binding.

### Posterior group Hox TFs display a range of abilities to bind inaccessible chromatin

**Fig. 6.**
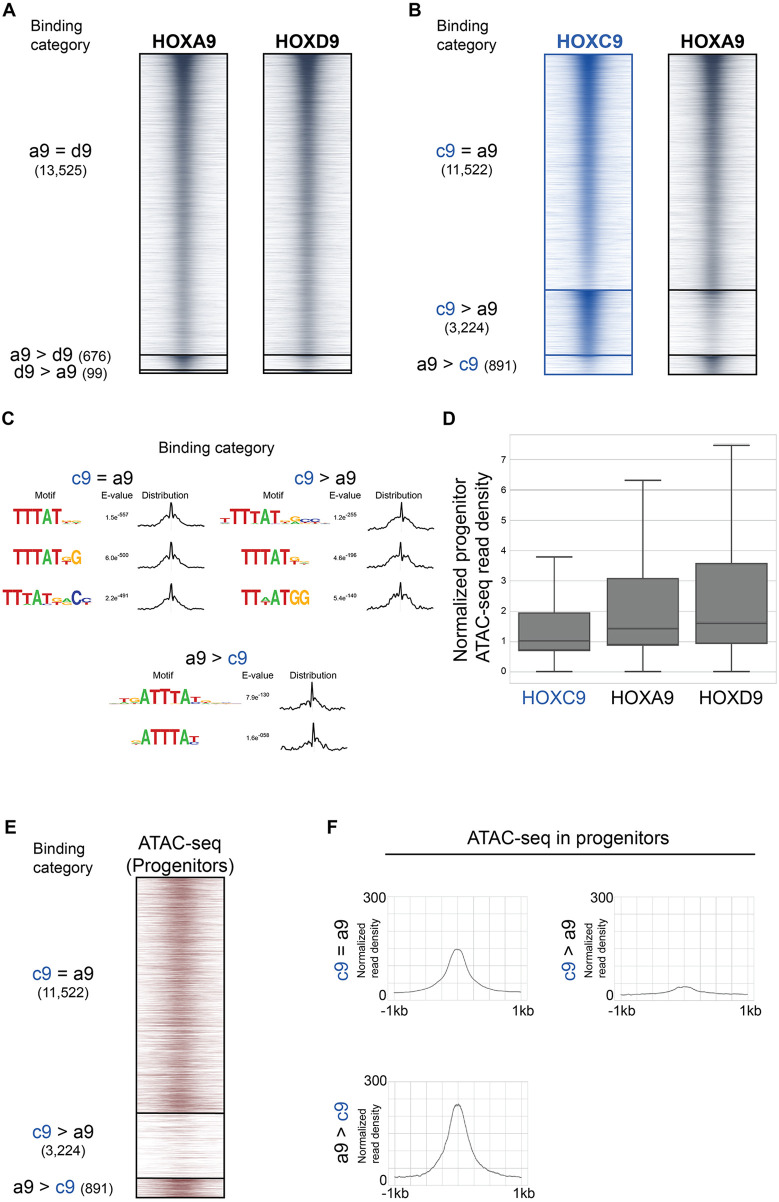
**HOXC9 binds to a larger fraction of sites in inaccessible chromatin than HOXA9 and HOXD9.** (A,B) ChIP-seq heatmap showing binding comparisons of the indicated Hox TFs in differentiating neurons at day 3 (*n*=2 independent differentiations). Sites bound by both indicated Hox TFs noted as ‘=’ sites. Preferentially bound sites by HOXA9, HOXD9 or HOXC9 noted as ‘a9 >’, ‘d9 >’ or ‘c9 >’. (C) Selected top enriched motifs discovered via MEME-ChIP at the indicated Hox binding categories. Distributions to the right of each motif show the distribution of each motif occurrence with respect to the midpoint of each peak (500 bp windows). (D) The distribution of day 2 progenitor ATAC-seq read density at the top 10,000 HOXC9, HOXA9 and HOXD9 sites at day 3. Data are ordered based on normalized read density (tags per million per site) and divided into quartiles. Boxes display the central 50% (quartile 2 and quartile 3), and the top and bottom whiskers represent the top 25% and bottom 25% (the top and bottom quartiles) of the data, respectively. (E) ATAC-seq heatmap displaying the accessibility in day 2 progenitors (before Hox induction) at the indicated Hox binding categories. (F) Metagene plots of accessibility in progenitors displaying the prior accessibility (before Hox induction) at the indicated binding categories. Normalized read density represents tags per million per 1000 sites.

We next wondered whether the ability of HOXC9 to bind to a large set of previously inaccessible sites is unique among posterior Hox TFs. Thus, we compared the binding of HOX9 paralog group TFs. A comparison of induced HOXA9 and HOXD9 ChIP-seq using MultiGPS revealed that they share a majority of their binding sites (Fig. 6A). However, comparing HOXC9 and HOXA9 binding patterns showed that HOXC9 uniquely binds an additional large category of sites (Fig. 6B). Suggesting a shared sequence preference, the detected motifs at sites bound by HOXA9 and HOXC9 resembled the posterior Hox TTTAT motif (Fig. 6C). HOXC9 bound to sites with the lowest median prior accessibility among the HOX9 paralogs (Fig. 6D). Accordingly, the sites uniquely bound by HOXC9 showed lower prior accessibility than other sets of sites (Fig. 6E,F). Hence, our results suggest that there is a divergence in the ability to bind inaccessible sites, even within the HOX9 posterior paralog group.

**Fig. 7.**
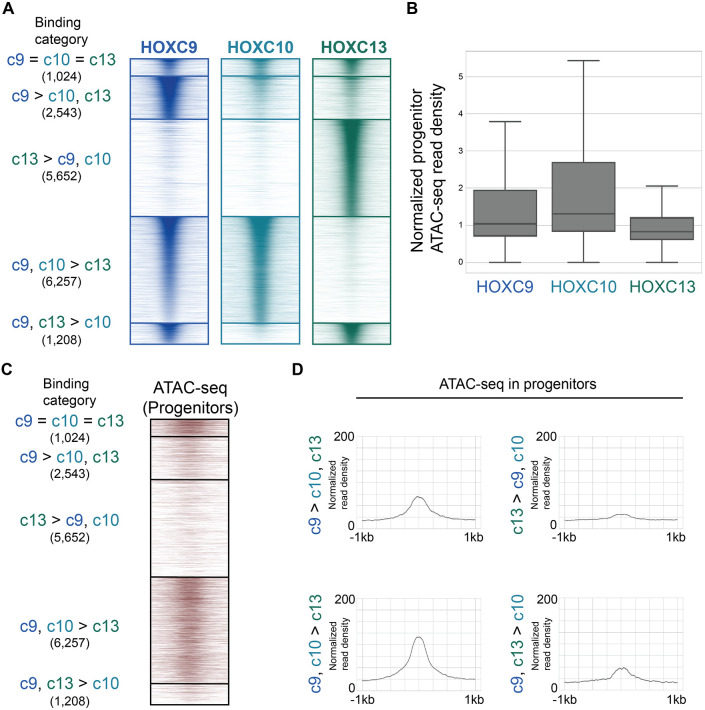
**Posterior group Hox TFs display a range of abilities to bind inaccessible chromatin.** (A) ChIP-seq heatmap showing binding comparisons of HOXC9, HOXC10 and HOXC13 in differentiating neurons at day 3 (*n*=2 independent differentiations). Sites bound by all three Hox TFs are indicated as ‘c9=c10=c13’ sites. Preferentially bound sites by HOXC9, HOXC13, HOXC9 and HOXC10 or HOXC9 and HOXC13 are indicated as ‘c9>c10, c13’, ‘c13>c9, c10’, ‘c9, c10>c13’ and ‘c9, c13>c10’, respectively. (B) The distribution of day 2 progenitor ATAC-seq read density at the top 10,000 HOXC9, HOXC10 and HOXC13 sites at day 3. Data are ordered based on normalized read density (tags per million per site) and divided into quartiles. Boxes display the central 50% (quartile 2 and quartile 3), and the top and bottom whiskers represent the top 25% and bottom 25% (the top and bottom quartiles) of the data, respectively. (C) ATAC-seq heatmap displaying the accessibility in day 2 progenitors (before Hox induction) at the indicated Hox binding categories. (D) Metagene plots of accessibility in progenitors displaying the prior accessibility (before Hox induction) at the indicated binding categories. Normalized read density represents tags per million per 1000 sites.

The analyzed posterior Hox binding profiles revealed that they do not entirely overlap in their genomic binding, despite sharing motif preferences. Thus, we sought to expand these analyses to another relevant posterior Hox gene. *Hox13* paralogs are also posterior group genes but have the unique ability to terminate axial elongation. To assess whether HOXC13 would bind like the other posterior Hox TFs, we compared HOXC9, HOXC10 and HOXC13 genomic binding overlaps (Fig. 7A). Surprisingly, only a small fraction of all sites (1024) were shared by HOXC9, HOXC10 and HOXC13 (‘c9=c10=c13’). As in all previous comparisons, HOXC9 retained a subset of private sites 2543 (‘c9>c10,c13’). However, in this comparison, a large category (5652) of sites was bound by HOXC13 alone (‘c13>c9,c10’). A direct comparison of HOXC9 and HOXC13 binding profiles supported the finding that they primarily bind distinct sets of sites (Fig. S5A). Motif analysis at these sites revealed that HOXC13 binds distinct motifs containing the TTTAC sequence (Fig. S5B,C), in agreement with previous *in vitro* binding characterizations (Berger et al., 2008). Thus, HOXC13 has a distinct motif preference, thereby increasing the posterior Hox TF binding diversity.

We then asked whether the posterior Hox group TF binding diversity also correlates with their differential ability to bind to previously inaccessible chromatin. The distribution of progenitor ATAC-seq read density at HOXC9, HOXC10 and HOXC13 sites revealed that HOXC13 binding sites had the lowest median accessibility in MN progenitors, even lower than HOXC9 (Fig. 7B). Dissecting the accessibility at the different Hox binding categories underscored the ability of HOXC13 to bind to sites with the lowest prior accessibility (Fig. 7C,D). In agreement, recent *in vivo* studies revealed that the patterning activity of HOX13 paralogs during limb and genital development relies on their ability to increase accessibility at specific sites (Desanlis et al., 2020; Amandio et al., 2020).

**Fig. 8.**
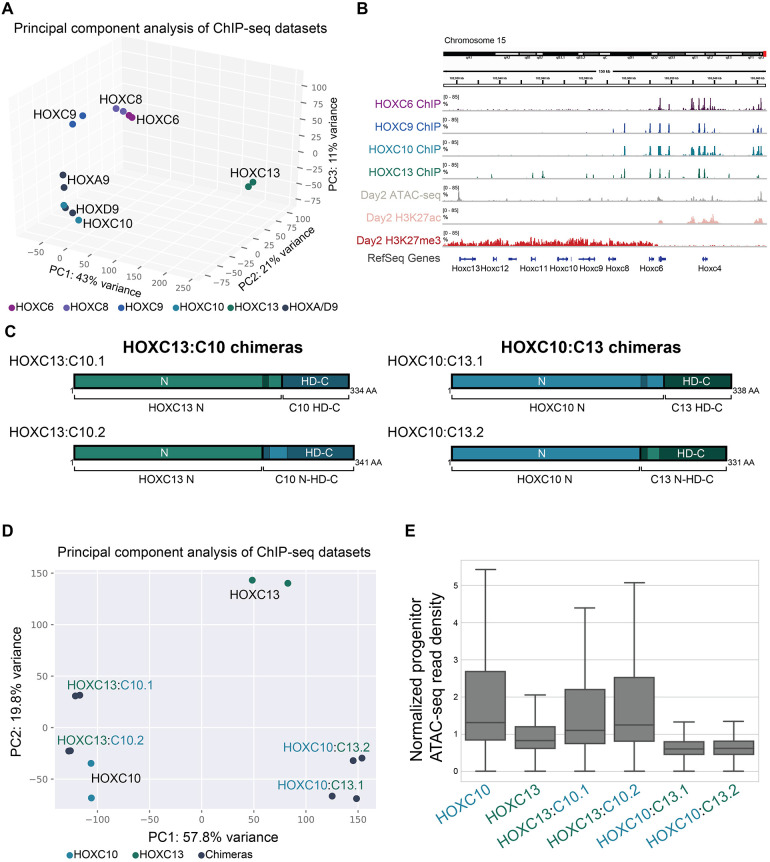
**The** **HD** **and C-terminus domain are responsible for binding to inaccessible regions.** (A) PCA of the ChIP-seq datasets reveals similarities in the binding patterns of Hox TFs (each dot represents independent differentiations). (B) Browser screenshots of the indicated Hox ChIP-seqs, day 2 ATAC-seq, H3K27ac and H3K27me3 ChIP-seqs at the *HoxC* gene cluster. All tracks are rescaled to the 85th percentile. (C) Schematic describing HOXC13:C10 and HOXC10:C13 chimeric Hox proteins. (D) PCA of the ChIP-seq datasets reveals similarities in the binding patterns of Hox TFs (each dot represents independent differentiations). (E) The distribution of day 2 progenitor ATAC-seq read density at the top 10,000 binding sites of the indicated Hox TF at day 3. Data are ordered based on normalized read density (tags per million per site) and divided into quartiles. Boxes display the central 50% (quartile 2 and quartile 3), and the top and bottom whiskers represent the top 25% and bottom 25% (the top and bottom quartiles) of the data, respectively.

To visualize the overall variation in Hox TF genome-wide binding profiles, we performed PCA on Hox TF ChIP-seq read counts associated with the top 10,000 binding sites for at least one Hox TF (Fig. 8A, Fig. S5D,E). PC1, which explains 43% of the variance between the TFs, separated HOXC13 from the central Hox TFs, HOX9 paralogs and HOXC10. On the other hand, PC2 and PC3, which cumulatively explain 32% of the variance, separated the TFs into three clusters. Although distinguishable, HOXC6 and HOXC8 clustered close to each other. HOXC9 clustered by itself, whereas HOXC10, HOXA9 and HOXD9 clustered together.

The binding pattern of HOXC TFs to the *HoxC* gene cluster demonstrated their differential abilities to engage inaccessible chromatin (Fig. 8B). HOXC6 only binds *Hoxc4-5* genes, which are transcriptionally active, whereas HOXC10, HOXC9 and HOXC13 bind progressively deeper into the cluster at repressed *HoxC* genes, which are covered in the catalytic product of PRC2 (Mazzoni et al., 2013; Narendra et al., 2015). We note that our visualization of Hox gain-of-function binding activities at the *HoxC* cluster serves only as a convenient example of the relative abilities of Hox TFs to bind inaccessible chromatin. In the embryo, progressive removal of polycomb repressive complexes from the Hox clusters, and activation of posterior Hox genes, might be more dependent on other signaling-responsive TFs (Mazzoni et al., 2013).

Thus, differences in genome-wide binding profiles of the Hox TFs reflect sequence preferences, as well as the differential abilities of the Hox TFs to bind previously inaccessible chromatin. Posterior Hox TFs can bind different genomic sites by either having similar sequence preferences and differing abilities to bind inaccessible chromatin (HOXC9 versus HOXC10), or differing in both aspects (HOXC9 versus HOXC13).

We wondered if variation in the HD is largely responsible for the differential abilities of Hox TFs to engage inaccessible chromatin. Thus, we generated chimeric Hox proteins by following the previously published logic of swapping the N-terminus (N) and DNA-binding domain+C-terminus (HD+C) (Lacombe et al., 2013). We chose HOXC10 and HOXC13 because of their low and high ability to bind inaccessible chromatin, respectively (Fig. 8C). PCA of HOXC10, HOXC13 and chimeric Hox proteins revealed that chimeras bind more similarly to the Hox protein that shares their DNA-binding domain (Fig. 8D). However, chimeric Hox proteins do not have identical genomic binding patterns to either HOXC10 or HOXC13, demonstrating that the N and HD-C together control overall genomic binding. Next, we investigated whether chimeras bind to inaccessible chromatin. This analysis revealed that chimeric Hox proteins with the HD-C of HOXC13 have a very high preference for inaccessible chromatin (Fig. 8E). Thus, the DNA-binding domain and C-terminus are sufficient for binding inaccessible chromatin.

Finally, to gain an overview of the relative dependence on the pre-existing chromatin environment of each Hox TF, we applied Bichrom to analyze the data (Srivastava et al., 2020 preprint). Bichrom is a neural network-based method that integrates DNA sequence and previous chromatin information to explain the observed genomic binding patterns of an induced TF. We train Bichrom to predict the binding patterns of each Hox TF using DNA sequence features and chromatin tracks from day 2 progenitors (ATAC-seq and ChIP-seq for H3K4me3, H3K27ac, H3K27me3, H3K9me3 and Pol II). We then compared the predictive performance of Bichrom with that of a neural network that uses only sequence information. If all Hox TFs had similar reliance on chromatin states for binding, we would predict similar recall improvements across TFs when incorporating chromatin data in addition to the sequence. However, our data shows a variation that correlates with their preference for inaccessible chromatin (Fig. 4B, Fig. 6D, Fig. 7B). We found that HOXC9 and HOXC13 networks display minor improvements in the predictive performance when trained with or without neuronal progenitor chromatin data (Fig. S6A). HOXC8 and HOXC10 predictions benefit from included previous chromatin data, and HOXC6 and the other HOX9 paralogs (HOXA9 and HOXD9) display substantial gains in predictive performance when training includes chromatin tracks. These results support the hypothesis that even Hox TFs from the same group rely on previous chromatin states to different degrees for their genomic binding.

### The genomic binding of HOXC6, HOXC9 and HOXC10 correlates with differential gene expression

**Fig. 9.**
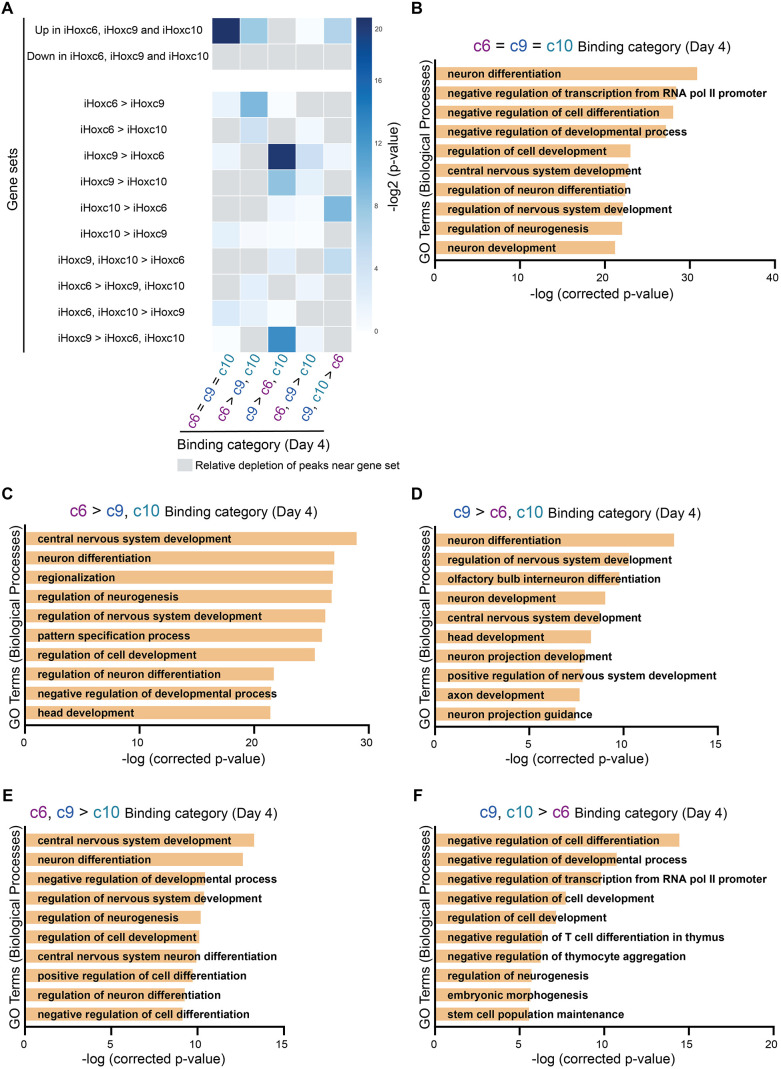
**Differentially expressed gene sets correlate with differential Hox binding events.** (A) Heatmap representing associations between Hox binding categories (day 4; from Fig. S3E) and the indicated gene sets (day 4). Upregulation/downregulation in iHoxc6, iHoxc9 and iHoxc10 (day 4) is relative to day 2 progenitors (before Hox induction). (B-F) Top GO-terms enriched at the indicated Hox binding categories (day 4; from Fig. S3E).

Finally, we investigated whether differentially expressed genes in the iHox neurons correlate with specific Hox binding categories. For this comparison, we focused on the three main spinal cord domains and their ‘canonical’ inducing TFs: HOXC6 for brachial, HOXC9 for thoracic and HOXC10 for lumbar. Specifically, we used the logistic regression-based ChIP-Enrich method to identify significant associations (adjusted *P*-value<0.01) between RNA-seq derived gene sets and Hox TF binding categories. Genes that are equally upregulated in iHoxc6, iHoxc9 and iHoxc10 neurons associated strongly with c6=c9=c10 sites, with some association with c6>c9,c10 and c9,c10>c6 sites as well (Fig. 9A). Genes differentially expressed in iHoxc6, compared with iHoxc9 or iHoxc10 neurons, showed a correlation with c6>c9,c10 sites (Fig. 9A). Similarly, genes differentially expressed in iHoxc9 neurons correlated with c9>c6,c10 sites (Fig. 9A). Thus, Hox TF binding correlates with transcriptional activity. Moreover, the enriched gene ontology (GO) terms at Hox binding sites were relevant to the phenotypes induced by the Hox TFs *in vivo* (Fig. 9B-F), even with all the limitations of GO term analysis for tissue segments. For example, neuron differentiation and central nervous system development appeared as the top GO terms at several binding categories. Also, several GO terms on axon development and guidance appeared at sites preferentially bound by HOXC9 (Fig. 9D), which are crucial MN features well documented to be downstream of Hox patterning.

## DISCUSSION

Hox TFs have crucial roles in body patterning during animal development. However, surprisingly little is known about how vertebrate Hox TFs bind to the genome in a cellular-relevant environment. This is exacerbated for posterior group Hox genes that pattern distinct vertebrate structures, albeit sharing a common *Drosophila* ortholog gene. To gain insights into Hox activity, we performed a multilevel comparison of global binding patterns, chromatin accessibility preferences and transcriptional target genes of seven Hox proteins expressed under the same developmentally relevant conditions. Although the data show intrinsic sequence preferences that differ between Hox TFs, we found that a major determinant of genomic binding diversity among posterior Hox TFs were differential abilities to bind inaccessible chromatin. Therefore, posterior Hox TF patterning may diverge by mostly tuning chromatin accessibility binding rather than sequence preference. Although we have not shown that they bind target sequences on nucleosomes, this behavior is consistent with some posterior Hox TFs being pioneer factors (Zaret and Carroll, 2011).

The central group HOXC6 and HOXC8 TFs induce limb-innervating fate at brachial spinal cord levels. They appear to do so by binding to relatively similar sites in the genome compared with other analyzed Hox binding profiles. HOXC10 induces a similar limb-innervating fate at lumbar levels but it has a binding profile more similar to the thoracic HOXC9 (repressor of limb-level fates). Overall, these results suggest that similar cell fates are not always the product of identical Hox TF binding patterns. HOXC10 sequence preference is in line with the posterior group. However, the ability of HOXC10 to bind to inaccessible chromatin is more similar to central limb-innervating HOXC6 and HOXC8. Thus, HOXC10 diverges from HOXC9 by having a lower preference for inaccessible chromatin. The high preference of HOXC9 for inaccessible chromatin is not shared by other HOX9 paralogs. Thus, the results from this study may point to how members of the same paralog group diverge their patterning abilities. HOXC9 is a strong repressor of anterior Hox genes and limb-innervating fates (Jung et al., 2010,^2014^). *Hox13* paralogs pattern caudal and distal structures by inhibiting cell growth or inducing apoptosis (Economides et al., 2003; Godwin and Capecchi, 1998; Denans et al., 2015; Young et al., 2009). Our data point to an interesting correlation between binding inaccessible chromatin and these developmentally relevant functions.

The current study does not recapitulate all aspects of caudal spinal cord differentiation. During embryonic development, caudal spinal cord neurons are derived from neuromesodermal progenitors exposed to WNT/FGF patterning signals that among other events control Hox gene expression and chromatin (Lippmann et al., 2015; Gouti et al., 2014; Metzis et al., 2018; Mazzoni et al., 2013; Henrique et al., 2015). Thus, neuromesodermal progenitors expressing caudal Hox genes would have a different accessibility landscape. The current experimental set-up is designed to study differential Hox binding in a shared chromatin context. Future studies will be needed to capture Hox binding during development. Additionally, the *in vitro* differentiation strategy and forced expression of a single Hox gene does not recapitulate limb-innervating LMC neuron patterning into specific pools, nor does it induce thoracic PGC fate after *Hoxc9* overexpression (Dasen et al., 2005; Jung et al., 2010). Thus, Hox TFs might depend on neuromesodermal progenitor stages for these processes or they might require a more complex developmental context and patterning signals (Haase et al., 2002; Dasen et al., 2005; Arber et al., 2000). Additionally, *Hoxc10* overexpression induces some *Hoxa/c6* expression in this system. It will be interesting to test whether an *in vivo Hoxc10* overexpression induces additional limb-innervating Hox genes.

In agreement with *in vitro* binding preference studies, central and posterior Hox proteins bind to different motifs in neurons (Noyes et al., 2008; Mann et al., 2009; Berger et al., 2008). Central Hox TFs bind to sites with the central TAAT core motif, whereas posterior Hox TFs bind to TTAT core motifs. MEME-ChIP also discovered bipartite co-factor and Hox motifs with high enrichment in some sets of sites (Fig. 3A). An interaction with TALE co-factors MEIS and PBX can change the affinity and selectivity of Hox DNA binding (Slattery et al., 2011; reviewed by Mann and Chan, 1996; Merabet and Mann, 2016; Mann and Affolter, 1998). Besides, partnering with TALE co-factors modifies Hox binding specificity through the recognition of DNA shape (Abe et al., 2015; Joshi et al., 2007; Zeiske et al., 2018). Differential binding of the canonical MEIS Hox co-factor does not seem to explain differential Hox TF binding patterns (Fig. S7A). MEIS binding appears to follow or reflect Hox binding, as opposed to being exclusively associated with particular subsets of differentially bound sites.

Furthermore, PBX1-4 and MEIS1-3 expression levels are largely similar across the different Hox TF inductions and are thus unlikely to explain binding differences (Fig. 1D, Fig. S2A,B). We attempted similar experiments with PBX factors in two of the inducible Hox lines, but the PBX antibody produces a weak ChIP-seq signal (Fig. S7B). We also failed to detect a CTCF motif at Hox binding sites, which is reported to co-bind with some HOXA/D proteins (Jerković et al., 2017). Our data thus failed to identify a co-factor that explains differential Hox binding during MN differentiation. A systematic evaluation and perturbation of all possible Hox co-factors during cell differentiation will shed some light on this issue.

Our data suggest Hox binding, and thus patterning, models should integrate sequence and chromatin state to explain each Hox activity. Most Hox TFs would bind to cell-specific accessible sites with canonical motifs. Hence, HOXC6, HOXC9 and HOXC10 shared sites tend to be in regions with high prior accessibility during MN differentiation, and these sites appear to contain both types of Hox binding motifs. However, some Hox TFs can associate with sites in less accessible genomic regions. Thus, they become more independent of earlier chromatin patterning events. This ability lies in the HD and C-terminus, which suggests that the HD controls not only sequence preference but also the ability to engage with inaccessible sites. In sum, the data presented here suggest Hox TF patterning abilities can only be explained by integrating not just the sequence preference and co-factor interactions of each Hox TF, but also the pre-existing cell-specific chromatin landscape and the ability of the Hox TF to interact with inaccessible chromatin.

## MATERIALS AND METHODS

### Cell line generation

The inducible Hoxc6, Hoxc8 and Hoxc9 cell lines were generated as described previously (Mazzoni et al., 2011; published in Jung et al., 2010; Narendra et al., 2016; Tan et al., 2016). The inducible cassette exchange (ICE) system was used to generate all cell lines (Iacovino et al., 2011). The resulting inducible cell lines harbored a single copy of the transgene inserted at the expression-competent HPRT locus. Inducible GFP, Hoxa9, Hoxd9, Hoxc10 and Hoxc13 cell lines were generated for this study. Hoxa9, Hoxd9 and Hoxc13 cDNA was amplified using Phusion polymerase (Thermo Scientific) from pCAGGS mHoxA9 (JD-114), pCAGGS mHoxD9 (JD-237) and hHoxc13 cDNA (Dharmacon, accession BC090850), respectively, and Flag and HA tags were introduced during the amplification step at the amino- (N) or carboxyl (C)-terminus, respectively. p2lox plasmids were generated by In-Fusion cloning (Takara) the respective cDNAs into the p2lox plasmid backbone. The recipient mESCs were treated with 1 μg/ml Dox (Sigma-Aldrich, D9891) for 16 h to induce Cre recombinase expression before electroporation of the respective plasmids. After selection with G418 (400 μg/ml, Cellgro), cell lines were characterized by performing antibody staining for Flag (mouse anti-FLAG; Sigma-Aldrich, F1804) and HA (rabbit anti-HA; Abcam, ab9110), and expanded.

### Chimeric Hox plasmid generation

Chimeric Hox plasmids were generated by swapping regions that code for HD-C domains of Hoxc13 and Hoxc10. Additional chimeras were generated by taking into account a conserved region in the N-terminus, located upstream of the HD. HOXC13:HOXC10 chimeras have the following protein sequences: HOXC13:HOXC10.1 has amino acids 1-259 of HOXC13 and amino acids 268-342 of HOXC10 (total protein length is 334 amino acids); and HOXC13:HOXC10.2 has amino acids 1-230 of HOXC13 and amino acids 232-342 of HOXC10 (total protein length is 341 amino acids). HOXC10:HOXC13 chimeras have the following protein sequences: HOXC10:HOXC13.1 has amino acids 1-267 of HOXC10 and amino acids 260-330 of HOXC13 (total protein length is 338 amino acids); and HOXC10:HOXC13.2 has amino acids 1-231 of HOXC10 and amino acids 231-330 of HOXC13 (total protein length is 331 amino acids). cDNAs were amplified using Phusion polymerase and Flag tags were introduced during the amplification step at the N. p2lox plasmids were generated by In-Fusion cloning (Takara) the respective cDNAs into the p2lox plasmid backbone. The ICE system was used to generate cell lines (Iacovino et al., 2011).

### Cell culture

mESC lines were cultured in 2-inhibitors-based medium [advanced Dulbecco's modified eagle medium (DMEM)/F12:Neurobasal (1:1) medium (Gibco), supplemented with 2.5% ESC-grade fetal bovine serum (v/v, Corning), N2 (Gibco), B27 (Gibco), 2 mM L-glutamine (Gibco), 0.1 mM β-mercaptoethanol (Gibco), 1000 U/ml leukemia inhibitory factor (Millipore), 3 μM CHIR (BioVision) and 1 μM PD0325901 (Sigma-Aldrich)] on 0.1% gelatin-coated (Millipore) plates at 37°C and 8% CO_2_. *In vitro* differentiation of mESCs to MNs has been described previously (Tan et al., 2016; Wichterle et al., 2002; Wichterle and Peljto, 2008). Briefly, embryoid bodies (EBs) were obtained by plating trypsinized (Gibco) mESCs in AK medium [advanced DMEM/F12:Neurobasal (1:1) medium (Gibco), 7% KnockOut SR (v/v) (Gibco), 2 mM L-glutamine, 0.1 mM β-mercaptoethanol and penicillin–streptomycin (Gibco)] at 37°C, and 5% CO_2_ (day −2). On day 0, EBs were split 1:2 and AK medium was replenished and supplemented with 1 μM all-trans RA and 0.5 μM smoothened agonist (SAG) (Millipore, 566660). TF induction was performed by adding 3 μg/ml of Dox (Sigma-Aldrich, D9891) on day 2. For RNA-seq and ATAC-seq experiments, 3.5×10^5^ mESCs were plated in 100 mm suspension dishes (Corning). For ChIP-seq experiments, 3-3.5×10^6^ mESCs were plated in 245 mm×245 mm square dishes (Corning).

### ChIP-seq

Cells were collected 24 h and 48 h after Dox treatment (day 3 and 4 of RA/SAG differentiation). Crosslinking was performed at room temperature in 1 mM DSG (ProteoChem) for 15 min, followed by the addition of 1% FA (v/v) for an additional 15 min. After quenching with glycine, cells were washed with 1×PBS, divided into ∼25-30×10^6^ aliquots, pelleted by centrifugation at 275 ***g*** and frozen at −80°C. After thawing cells on ice, lysis was performed in 5 ml of 50 mM HEPES-KOH (pH 7.5), 140 mM NaCl, 1 mM EDTA (pH 8.0), 10% glycerol (v/v), 0.5% Igepal (v/v), 0.25% Triton X-100 (v/v) with 1× protease inhibitors (Roche, 11697498001) for 10 min at 4°C. Cells were centrifuged at 1200 ***g*** for 5 min, resuspended in 5 ml of 10 mM Tris-HCl (pH 8.0), 200 mM NaCl, 1 mM EDTA (pH 8.0), 0.5 mM EGTA (pH 8.0) with 1× protease inhibitors, and incubated for 10 min at 4°C on a rotating platform. Cells were centrifuged at 1200 ***g*** for 5 min and resuspended in 2 ml of sonication buffer [50 mM HEPES (pH 7.5), 140 mM NaCl, 1 mM EDTA (pH 8.0), 1 mM EGTA (pH 8.0), 1% Triton X-100 (v/v), 0.1% sodium deoxycholate (w/v), 0.1% SDS (v/v) with 1× protease inhibitors]. Sonication was performed by splitting each sample in two Bioruptor tubes with sonication beads and using the Bioruptor Pico (Diagenode) for 18 cycles of 30 s on and 30 s off to sheer crosslinked DNA into an average size of ∼200 bp. Immunoprecipitation was performed for 16 h at 4°C on a rotating platform by incubating in 0.5% bovine serum albumin solution with Dynabeads protein G (Thermo Fisher Scientific) conjugated with 5 µg of one of the following antibodies (all at a dilution of 2.5 µg/ml): mouse monoclonal to Flag (Sigma-Aldrich, F1804); rabbit polyclonal to HA (Abcam, ab9110); rabbit polyclonal to V5 (Abcam, ab15828); mouse monoclonal to Pbx1/2/3/4 (Santa Cruz Biotechnology, sc-28313); or goat polyclonal to Meis1/2 (Santa Cruz Biotechnology, sc-10599). Following the immunoprecipitation, washes were performed with the following buffers (ice cold): sonication buffer; sonication buffer with 500 mM NaCl; LiCl wash buffer [20 mM Tris-HCl (pH 8.0), 1 mM EDTA (pH 8.0), 250 mM LiCl, 0.5% Igepal (v/v) and 0.5% sodium deoxycholate (w/v)]; and TE buffer [10 mM Tris-HCl (pH 8.0) and 1 mM EDTA (pH 8.0)]. Elution was performed by incubation in elution buffer [50 mM Tris-HCl (pH 8.0), 10 mM EDTA (pH 8.0) and 1% SDS (v/v)] for 45 min at 65°C. Reversal of crosslinks was performed by incubation for 16 h at 65°C. RNA digestion was performed by the addition of 200 μl of TE and RNase A (Sigma-Aldrich) at a final concentration of 0.2 mg/ml, and incubation for 2 h at 37°C. Proteinase K (Invitrogen) was added at a final concentration of 0.2 mg/ml, supplemented with CaCl_2_, to digest protein at 55°C for 30 min. DNA was purified with phenol:chloroform:isoamyl alcohol (25:24:1; v/v) (Invitrogen) and by performing ethanol precipitation. DNA pellets were resuspended in water. lllumina DNA sequencing libraries were prepared with one third of the ChIP sample or a 1:100 dilution of the input sample in water. Library preparation was performed by end repair, A-tailing and ligating Illumina-compatible Bioo Scientific multiplexed adapters. Unligated adapters were removed using Agencourt AmpureXP beads (Beckman Coulter). Amplification was performed by PCR with Phusion polymerase (New England Biolabs) and TruSeq primers (Sigma-Aldrich). Libraries were gel purified (Qiagen) between 250 and 550 bp in size. Final quantification of the library was performed using a KAPA Library Amplification kit on a Roche LightCycler 480 before pooling. The libraries were sequenced on an Illumina NextSeq 500 using V2 and V2.5 chemistry (75 cycles, single-end 75 bp) or on an Illumina NovaSeq 6000 using the SP Reagent Kit (100 cycles, single-end 100 bp) at the Genomics Core Facility at New York University. The H3K27me3 and H3K27ac ChIP-seq datasets in Fig. 8B were published previously in Mazzoni et al. (2013) and Rhee et al. (2016), respectively.

### RNA-seq

Cells were collected before TF induction (day 2 of RA/SAG differentiation) and 48 h after Dox treatment (day 4 of RA/SAG differentiation). RNA was extracted by using TRIzol LS Reagent (Life Technologies) and purified using the RNAeasy Mini Kit (Qiagen). Agilent High Sensitivity RNA Screentape (Agilent, 5067-5579) was used to check RNA integrity. A 500 ng quantity of RNA was used to prepare RNA-seq libraries and spiked-in with ERCC ExFold Spike-In mixes (Thermo Fisher Scientific, 4456739). RNA-seq libraries were prepared using a TruSeq Stranded mRNA Library Prep kit (Illumina, 20020594). Library size was verified using High Sensitivity DNA ScreenTape (Agilent, 5067-5584). The KAPA Library Amplification kit was used on a Roche LightCycler 480 for library quantification before pooling. The libraries were sequenced on an Illumina NextSeq 500 using V2.5 chemistry (75 cycles, single-end 75 bp) at the Genomics Core Facility at New York University.

### ATAC-seq

Cells were collected before TF induction (day 2 of RA/SAG differentiation) and 48 h after Dox treatment (day 4 of RA/SAG differentiation). Cells (50,000) were aliquoted and washed twice in ice-cold 1× PBS. Cell pellets were resuspended in 10 mM Tris (pH 7.4), 10 mM NaCl, 3 mM MgCl_2_ and freshly added 0.1% NP-40 (v/v), and centrifuged at 500 ***g*** for 10 min at 4°C. Pellets were resuspended in 25 µl of 2× tagmentation DNA buffer, 2.5 µl TDE1 (Nextera DNA Sample Preparation Kit, FC-121–1030) and 22.5 µl of water, and incubated at 37°C for 30 min. The sample was purified using the MinElute PCR Purification Kit (Qiagen, 28004). A qPCR reaction with 1× SYBR Green (Invitrogen), custom-designed primers and 2× NEB Master Mix (New England Labs, M0541) was performed to determine the optimal number of PCR cycles (one third of the maximum measured fluorescence) (Buenrostro et al., 2013). PCR enrichment of the library was performed with custom-designed primers and 2× NEB Master Mix. The libraries were purified using the MinElute PCR Purification Kit. High Sensitivity DNA ScreenTape (Agilent, 5067-5584) was used to verify the fragment length distribution of the library. Library quantification was performed using the KAPA Library Amplification kit on a Roche LightCycler 480. The libraries were sequenced on an Illumina NextSeq 500 using V2 and V2.5 chemistry (150 cycles, paired-end 75 bp) at the Genomics Core Facility at New York University.

### ChIP-seq data processing

ChIP-seq reads were aligned to the mm10 genome using Bowtie (v1.0.1) (Langmead et al., 2009), using options ‘-q --best --strata -m 1 --chunkmbs 1024’. Genome-wide TF binding events were called in each condition using MultiGPS (v0.74) (Mahony et al., 2014). EdgeR (v3.22.5) was used within the MultiGPS framework to test whether genomic sites were differentially bound by TFs (Robinson et al., 2010). Specifically, edgeR uses a negative binomial generalized linear model (GLM) to test whether ChIP-seq reads in one condition are significantly greater than in an alternative condition. A genomic site was defined as shared by TFs if significant binding events were called in both TF ChIP-seq experiments (q-value<0.001) and the TFs did not display differential read enrichment at that site as estimated by edgeR (q-value>0.01). A binding event was defined as ‘TF1>TF2’ if a MultiGPS peak was called in TF1 ChIP-seq (q-value<0.001), TF1 exhibited a greater log fold-change with respect to the input ChIP-seq than TF2, and TF1 and TF2 were significantly differentially bound as defined by edgeR (q-value<0.01). A similar strategy was applied to perform multi-way ChIP-seq comparisons. For example, when comparing HOXC6, HOXC9 and HOXC10 ChIP-seq experiments, ‘shared’ binding sites were defined when significant binding events were detected in all three ChIP-seq datasets and no two TFs were differentially bound with respect to each other. Sites were defined as ‘TF1> TF2, TF3’ if a significant binding event was called for TF1 and a significantly greater number of reads were detected by edgeR in TF1 ChIP-seq compared to both TF2 and TF3. Finally, ‘TF1, TF2>TF3’ events were defined when significant binding events were called in both TF1 and TF2, no differential binding was detected between TF1 and TF2, and both TF1 and TF2 had a significantly greater number of reads than TF3. Only binding categories containing at least 500 binding events were retained. For example, consistent with HOXC10 binding to a subset of HOXC9 sites, fewer than 200 sites were categorized as preferentially bound by HOXC10 alone when comparing HOXC6, HOXC9 and HOXC10 binding. The union of the top 10,000 binding sites for each TF (38,874 unique genomic locations in Fig. 8A) was used to perform PCA.

### RNA-seq data processing

Fastq files obtained from RNA-seq were aligned to the genome using the splice-aware STAR (Spliced Transcripts Alignment to a Reference) aligner (v2.7.0c) (Dobin and Gingeras, 2016). Mapped reads were assigned to NCBI RefSeq annotated mm10 genes using the featureCount function in Rsubread (v1.30.9) (Liao et al., 2019). RefSeq genes with matching Entrez IDs were merged into a single gene by Rsubread. Following read summarization, read counts were normalized using the ‘rlog’ or regularized log transformation in DESeq2 (v1.20.0) (Love et al., 2014). Transformed read counts were used as input features into the dimensionality reduction techniques PCA and MDS. The log_2_ fold change (LFC) in gene expression levels between iHox neurons versus day 2 progenitors or control neurons (no Dox treatment) was estimated using DESeq2. A q-value<0.01 and LFC>2 was used to define differentially expressed genes between day 2 progenitors, control neurons, iHoxc6, iHoxc8, iHoxc9 and iHoxc10 neurons. We filtered out genes that were not expressed in any iHox neuron, retaining 19,019 genes.

### ATAC-seq data processing

Paired-end ATAC-seq reads were mapped to the mouse mm10 genome using Bowtie2 (v2.2.2) (Langmead et al., 2009). Genome-wide ATAC-seq-derived accessible domains were defined using DomainFinder in the SeqCode project (www.github.com/seqcode/seqcode-core/blob/master/src/org/seqcode/projects/seed/DomainFinder.java).

### ChIP-seq and ATAC-seq data visualization

Heatmaps were used to plot the ChIP-seq and ATAC-seq reads at multiple categories of genomic sites in iHox neurons. Heatmaps were made using the MetaMaker program in SeqCode (www.github.com/seqcode/seqcode-core/blob/master/src/org/seqcode/viz/metaprofile/MetaMaker.java). Raw reads from the ChIP-seq data were extended to 100 bp and read counts were binned into 100 bp bins. Binned reads were plotted over 1000 bp windows centered on MultiGPS binding events. Color thresholds used to produce heatmaps were determined from binding data using the MetaPlotLineMaxEstimator program in SeqCode. Specifically, all 100 bp bins were ordered by the number of reads overlapping each bin. The maximum value for the heatmap color scale was set to the number of read counts at the 85th percentile bin. The minimum value for all heatmaps was set to five reads. Finally, all binding events represented were ordered based on peak strength (as defined by MultiGPS *P*-values).

### ATAC-seq composite plots

For each ATAC-seq experiment, reads mapping to mitochondrial DNA or unannotated regions were filtered out. All experiments were performed in replicates; composite read distributions were initially calculated independently for each replicate. Filtered bedfiles were used to calculate the total number of reads overlapping each position in a 2000 bp window surrounding a binding event of interest. The number of reads at each position was summed over the set of genomic sites being analyzed. The reads were total tag normalized, and, finally, the resulting read counts were averaged over replicates. Read density (reads per million per site or reads per million per 1000 sites) was plotted using Seaborn in Python.

### RNA-seq data visualization

The LFC of gene expression levels in iHox neurons versus day 2 progenitors or control neurons (no Dox treatment) was estimated using DESeq2. The LFC values were plotted for previously established marker genes in iHoxc6 neurons versus day 2 progenitors, iHoxc8 neurons versus day 2 progenitors, iHoxc9 neurons versus day 2 progenitors, iHoxc10 neurons versus day 2 progenitors and control neurons versus day 2 progenitors (Fig. 1D). The LFC values were plotted for previously established marker genes in iHoxc6 versus control neurons, iHoxc8 versus control neurons, iHoxc9 versus control neurons and iHoxc10 versus control neurons (Fig. S2A). Volcano-style plots were used to simultaneously plot the LFC and *P*-values of significantly differentially expressed marker genes in the iHox versus control neuron comparisons (Fig. S2B). Volcano plots were used to represent the overall differential expression landscape between iHox and control neurons (Fig. S1D).

### GO term enrichment analysis

The Genomic Regions Enrichment of Annotation Tool (GREAT, v4.0.4) (McLean et al., 2010) was used to perform GO term enrichment analysis (Fig. 9B-F) and generate the graphs in Fig. S4A. GREAT first calculates the probability that a randomly selected cis-regulatory element will be associated with a given GO term. It then uses a binomial test parameterized by this probability to determine whether a predefined subset of ChIP-seq peaks is significantly associated with that GO term. The process is repeated for all GO terms and a list of significant associations is returned (McLean et al., 2010). We used GREAT to perform GO term enrichment analysis for the HOXC6-only, HOXC9-only, HOXC6 and C9, HOXC9 and C10, and shared Hox ChIP-seq binding sites. The top ten significant GO terms were plotted; ordered by their Bonferroni-corrected *P*-values.

### Association between gene sets and binding events

In order to construct gene sets, pairwise comparisons of gene expression levels between iHoxc6, iHoxc9 and iHoxc10 neurons versus day 2 progenitors were performed using DESeq2. Additionally, pairwise comparisons were also performed between the iHox neurons: iHoxc6 versus iHoxc9, iHoxc6 versus iHoxc10 and iHoxc9 versus iHoxc10. Genes that were upregulated in all iHox versus day 2 progenitor comparisons (LFC>2 and adjusted *P*<0.01), as well as not differentially expressed between the iHox neurons (|LFC|>2), were assigned to the gene set ‘shared-upregulated’. A similar logic was used to identify the ‘shared-downregulated’ genes. Pairwise comparisons between iHox neurons were used to identify genes significantly upregulated or downregulated (|LFC|>2 and adjusted *P*<0.01) in individual iHox neurons. Genes that were significantly upregulated or downregulated in two out of three iHox neurons were assigned to gene sets using the same thresholds.

In order to test whether specific categories of ChIP-seq binding events were associated with differentially regulated gene sets, we used ChIP-Enrich (v.1.10.0). Specifically, each peak was assigned to the gene with the nearest transcriptional start site. ChIP-Enrich uses a logistic regression model to test whether gene set membership (controlled by mappable gene length) predicts whether a gene is associated with a ChIP-seq peak (Welch et al., 2014). The significance of the weight associated with gene set membership is estimated using the ‘Wald’ statistic. *P*-values obtained from the Wald statistic were adjusted using the Benjamini-Hochberg multiple testing correction (Welch et al., 2014). For each category of binding events, adjusted *P*-values were plotted for each predefined gene set.

### DNA motif analysis

*De novo* motif discovery was performed using MEME-ChIP (v. 5.1.0) (Machanick and Bailey, 2011) with the following command-line settings: ‘-ccut 100 -dna -meme-p 4 -meme-mod anr -meme-minw 5 -meme-maxw 15 -meme-nmotifs 10 -dreme-e 0.05’. MEME-ChIP was also provided with knowledge of the JASPAR CORE motif database (2016 release) (Mathelier et al., 2016) via the ‘-db’ option for the purposes of Tomtom motif similarity matching and CentriMo analysis. To find *de novo* discovered motifs that matched canonical cognate Hox TF binding motifs, we first extracted MEME or DREME discovered motifs that received significance scores of less than 1e-3 from those tools. We then used STAMP (v. 1.0) (Mahony and Benos, 2007) with settings ‘-cc PCC -align SWU’ to match against the following motif consensus sequences: TAATDR, HHATAAA, TAAT, ATAAA, GTAAA, TAAAC and TAAAT. Motifs were retained as probable cognate Hox motifs if they matched one of the above consensus sequences with a STAMP E-value score of less than 1e-4. Further motif frequency analysis was performed for two motifs that were discovered by DREME in the c6>c9,c10 and c9>c6,c10 categories (Fig. 3B). A motif scanning procedure using log-likelihood scoring was used to find peaks that contained motif hits within 50 bp of the peak positions. Motif hits were defined as sequences scoring above motif scanning thresholds set using a 0.1 false discovery rate derived from 1 million sequences (100 bp) randomly generated from a second-order Markov model of the mouse genome. Over-representation of peaks containing motifs was assessed in comparison with 10,000 sequences (100 bp) randomly sampled from the mouse genome. Finally, SeqUnwinder (v. 0.1.3) (Kakumanu et al., 2017) was used to find motifs that discriminate between various Hox binding site categories using the following command-line settings: ‘--threads 10 --makerandregs -- --win 150 --mink 4 --maxk 5 --r 10 --x 3 --a 400 --hillsthresh 0.1 --memesearchwin 16’.

### Bichrom data analysis

For each Hox TF, we first trained a hybrid convolutional and long short-term memory neural network to predict induced Hox binding using only DNA sequence information (seqnet). We then applied Bichrom to the Hox TFs, which integrates DNA sequence and pre-existing chromatin to predict induced TF binding. In particular, Bichrom takes the trained sequence-only network and incorporates additional chromatin data using a secondary chromatin sub-network (Srivastava et al., 2020 preprint). Day 2 progenitor ATAC-seq, H3K27ac, H3K4me3, H3K9me3, H3K27me3 and PolII were used to define the pre-existing chromatin landscape. For each chromatin experiment, the tag counts at each genomic window were total tag normalized, averaged across replicates and binned into 50 bp bins. The binned chromatin tracks were used as inputs into the chromatin sub-network of Bichrom. To account for computational variation in network performance, we repeated the training process on nine distinct training sets, each corresponding to a separate held-out test set (chromosome). We used the area under the precision-recall curve to measure the genome-wide predictive performance of the sequence-only neural network and Bichrom on the seven Hox TFs. The hyperparameters for all networks were selected using a hyperparameter grid search, and the networks were built using Keras (www.github.com/seqcode/iTF).

## Supplementary Material

Supplementary information

Reviewer comments

## References

[DEV194761C1] Abe N., Dror I., Yang L., Slattery M., Zhou T., Bussemaker H. J., Rohs R. and Mann R. S. (2015). Deconvolving the recognition of DNA shape from sequence. *Cell* 161, 307-318. 10.1016/j.cell.2015.02.008 25843630 PMC4422406

[DEV194761C2] Affolter M., Slattery M. and Mann R. S. (2008). A lexicon for homeodomain-DNA recognition. *Cell* 133, 1133-1135. 10.1016/j.cell.2008.06.008 18585344

[DEV194761C3] Akam M. (1989). Hox and HOM: homologous gene clusters in insects and vertebrates. *Cell* 57, 347-349. 10.1016/0092-8674(89)90909-4 2566382

[DEV194761C4] Amandio A. R., Lopez-Delisle L., Bolt C. C., Mascrez B. and Duboule D. (2020). A complex regulatory landscape involved in the development of mammalian external genitals. *eLife* 9, e52962 10.7554/eLife.52962 32301703 PMC7185996

[DEV194761C5] Arber S., Ladle D. R., Lin J. H., Frank E. and Jessell T. M. (2000). ETS gene Er81 controls the formation of functional connections between group Ia sensory afferents and motor neurons. *Cell* 101, 485-498. 10.1016/S0092-8674(00)80859-4 10850491

[DEV194761C6] Beh C. Y., El-Sharnouby S., Chatzipli A., Russell S., Choo S. W. and White R. (2016). Roles of cofactors and chromatin accessibility in Hox protein target specificity. *Epigenet. Chromatin* 9, 1 10.1186/s13072-015-0049-x PMC4705621 26753000

[DEV194761C7] Berger M. F., Badis G., Gehrke A. R., Talukder S., Philippakis A. A., Peña-Castillo L., Alleyne T. M., Mnaimneh S., Botvinnik O. B., Chan E. T. et al. (2008). Variation in homeodomain DNA binding revealed by high-resolution analysis of sequence preferences. *Cell* 133, 1266-1276. 10.1016/j.cell.2008.05.024 18585359 PMC2531161

[DEV194761C8] Buenrostro J. D., Giresi P. G., Zaba L. C., Chang H. Y. and Greenleaf W. J. (2013). Transposition of native chromatin for fast and sensitive epigenomic profiling of open chromatin, DNA-binding proteins and nucleosome position. *Nat. Methods* 10, 1213-1218. 10.1038/nmeth.2688 24097267 PMC3959825

[DEV194761C9] Dasen J. S. and Jessell T. M. (2009). Hox networks and the origins of motor neuron diversity. *Curr. Top. Dev. Biol.* 88, 169-200. 10.1016/S0070-2153(09)88006-X 19651305

[DEV194761C10] Dasen J. S., Liu J.-P. and Jessell T. M. (2003). Motor neuron columnar fate imposed by sequential phases of Hox-c activity. *Nature* 425, 926-933. 10.1038/nature02051 14586461

[DEV194761C11] Dasen J. S., Tice B. C., Brenner-Morton S. and Jessell T. M. (2005). A Hox regulatory network establishes motor neuron pool identity and target-muscle connectivity. *Cell* 123, 477-491. 10.1016/j.cell.2005.09.009 16269338

[DEV194761C12] Dasen J. S., DE Camilli A., Wang B., Tucker P. W. and Jessell T. M. (2008). Hox repertoires for motor neuron diversity and connectivity gated by a single accessory factor, FoxP1. *Cell* 134, 304-316. 10.1016/j.cell.2008.06.019 18662545

[DEV194761C13] Davis-Dusenbery B. N., Williams L. A., Klim J. R. and Eggan K. (2014). How to make spinal motor neurons. *Development* 141, 491-501. 10.1242/dev.097410 24449832

[DEV194761C14] De Kumar B., Parker H. J., Parrish M. E., Lange J. J., Slaughter B. D., Unruh J. R., Paulson A. and Krumlauf R. (2017). Dynamic regulation of Nanog and stem cell-signaling pathways by Hoxa1 during early neuro-ectodermal differentiation of ES cells. *Proc. Natl. Acad. Sci. USA* 114, 5838-5845. 10.1073/pnas.1610612114 28584089 PMC5468655

[DEV194761C15] Denans N., Iimura T. and Pourquié O. (2015). Hox genes control vertebrate body elongation by collinear Wnt repression. *eLife* 4, e04379 10.7554/eLife.04379 PMC4384752 25719209

[DEV194761C16] Desanlis I., Kherdjemil Y., Mayran A., Bouklouch Y., Gentile C., Sheth R., Zeller R., Drouin J. and Kmita M. (2020). HOX13-dependent chromatin accessibility underlies the transition towards the digit development program. *Nat. Commun.* 11, 2491 10.1038/s41467-020-16317-2 32427842 PMC7237422

[DEV194761C17] Dobin A. and Gingeras T. R. (2016). Optimizing RNA-Seq Mapping with STAR. *Methods Mol. Biol.* 1415, 245-262. 10.1007/978-1-4939-3572-7_13 27115637

[DEV194761C18] Donaldson I. J., Amin S., Hensman J. J., Kutejova E., Rattray M., Lawrence N., Hayes A., Ward C. M. and Bobola N. (2012). Genome-wide occupancy links Hoxa2 to Wnt-β-catenin signaling in mouse embryonic development. *Nucleic Acids Res.* 40, 3990-4001. 10.1093/nar/gkr1240 22223247 PMC3351182

[DEV194761C19] Duboule D. (2007). The rise and fall of Hox gene clusters. *Development* 134, 2549-2560. 10.1242/dev.001065 17553908

[DEV194761C20] Duboule D. and Dollé P. (1989). The structural and functional organization of the murine HOX gene family resembles that of Drosophila homeotic genes. *EMBO J.* 8, 1497-1505. 10.1002/j.1460-2075.1989.tb03534.x 2569969 PMC400980

[DEV194761C21] Duboule D. and Morata G. (1994). Colinearity and functional hierarchy among genes of the homeotic complexes. *Trends Genet.* 10, 358-364. 10.1016/0168-9525(94)90132-5 7985240

[DEV194761C22] Economides K. D., Zeltser L. and Capecchi M. R. (2003). Hoxb13 mutations cause overgrowth of caudal spinal cord and tail vertebrae. *Dev. Biol.* 256, 317-330. 10.1016/S0012-1606(02)00137-9 12679105

[DEV194761C23] Ekker S. C., Jackson D. G., VON Kessler D. P., Sun B. I., Young K. E. and Beachy P. A. (1994). The degree of variation in DNA sequence recognition among four Drosophila homeotic proteins. *EMBO J.* 13, 3551-3560. 10.1002/j.1460-2075.1994.tb06662.x 7914870 PMC395259

[DEV194761C24] Gehring W. J., Qian Y. Q., Billeter M., Furukubo-Tokunaga K., Schier A. F., Resendez-Perez D., Affolter M., Otting G. and Wüthrich K. (1994). Homeodomain-DNA recognition. *Cell* 78, 211-223. 10.1016/0092-8674(94)90292-5 8044836

[DEV194761C25] Godwin A. R. and Capecchi M. R. (1998). Hoxc13 mutant mice lack external hair. *Genes Dev.* 12, 11-20. 10.1101/gad.12.1.11 9420327 PMC316401

[DEV194761C26] Gouti M., Tsakiridis A., Wymeersch F. J., Huang Y., Kleinjung J., Wilson V. and Briscoe J. (2014). In vitro generation of neuromesodermal progenitors reveals distinct roles for wnt signalling in the specification of spinal cord and paraxial mesoderm identity. *PLoS Biol.* 12, e1001937 10.1371/journal.pbio.1001937 25157815 PMC4144800

[DEV194761C27] Haase G., Dessaud E., Garcès A., de Bovis B., Birling M.-C., Filippi P., Schmalbruch H., Arber S. and deLapeyriere O. (2002). GDNF acts through PEA3 to regulate cell body positioning and muscle innervation of specific motor neuron pools. *Neuron* 35, 893-905. 10.1016/S0896-6273(02)00864-4 12372284

[DEV194761C28] Henrique D., Abranches E., Verrier L. and Storey K. G. (2015). Neuromesodermal progenitors and the making of the spinal cord. *Development* 142, 2864-2875. 10.1242/dev.119768 26329597 PMC4958456

[DEV194761C29] Huang Y., Sitwala K., Bronstein J., Sanders D., Dandekar M., Collins C., Robertson G., Macdonald J., Cezard T., Bilenky M. et al. (2012). Identification and characterization of Hoxa9 binding sites in hematopoietic cells. *Blood* 119, 388-398. 10.1182/blood-2011-03-341081 22072553 PMC3257007

[DEV194761C30] Iacovino M., Bosnakovski D., Fey H., Rux D., Bajwa G., Mahen E., Mitanoska A., Xu Z. and Kyba M. (2011). Inducible cassette exchange: a rapid and efficient system enabling conditional gene expression in embryonic stem and primary cells. *Stem Cells* 29, 1580-1588. 10.1002/stem.715 22039605 PMC3622722

[DEV194761C31] Izpisúa-Belmonte J. C., Falkenstein H., Dollé P., Renucci A. and Duboule D. (1991). Murine genes related to the Drosophila AbdB homeotic genes are sequentially expressed during development of the posterior part of the body. *EMBO J.* 10, 2279-2289. 10.1002/j.1460-2075.1991.tb07764.x 1676674 PMC452918

[DEV194761C32] Jerković I., Ibrahim D. M., Andrey G., Haas S., Hansen P., Janetzki C., González Navarrete I., Robinson P. N., Hecht J. and Mundlos S. (2017). Genome-wide binding of posterior HOXA/D transcription factors reveals subgrouping and association with CTCF. *PLoS Genet.* 13, e1006567 10.1371/journal.pgen.1006567 28103242 PMC5289628

[DEV194761C33] Joshi R., Passner J. M., Rohs R., Jain R., Sosinsky A., Crickmore M. A., Jacob V., Aggarwal A. K., Honig B. and Mann R. S. (2007). Functional specificity of a Hox protein mediated by the recognition of minor groove structure. *Cell* 131, 530-543. 10.1016/j.cell.2007.09.024 17981120 PMC2709780

[DEV194761C34] Jung H., Lacombe J., Mazzoni E. O., Liem K. F. Jr, Grinstein J., Mahony S., Mukhopadhyay D., Gifford D. K., Young R. A., Anderson K. V. et al. (2010). Global control of motor neuron topography mediated by the repressive actions of a single hox gene. *Neuron* 67, 781-796. 10.1016/j.neuron.2010.08.008 20826310 PMC2955411

[DEV194761C35] Jung H., Mazzoni E. O., Soshnikova N., Hanley O., Venkatesh B., Duboule D. and Dasen J. S. (2014). Evolving Hox activity profiles govern diversity in locomotor systems. *Dev. Cell* 29, 171-187. 10.1016/j.devcel.2014.03.008 24746670 PMC4024207

[DEV194761C36] Jung H., Baek M., D'Elia K. P., Boisvert C., Currie P. D., Tay B.-H., Venkatesh B., Brown S. M., Heguy A., Schoppik D. et al. (2018). The ancient origins of neural substrates for land walking. *Cell* 172, 667-682.e15. 10.1016/j.cell.2018.01.013 29425489 PMC5808577

[DEV194761C37] Kakumanu A., Velasco S., Mazzoni E. and Mahony S. (2017). Deconvolving sequence features that discriminate between overlapping regulatory annotations. *PLoS Comput. Biol.* 13, e1005795 10.1371/journal.pcbi.1005795 29049320 PMC5663517

[DEV194761C38] Kmita M. and Duboule D. (2003). Organizing axes in time and space; 25 years of colinear tinkering. *Science* 301, 331-333. 10.1126/science.1085753 12869751

[DEV194761C39] Lacombe J., Hanley O., Jung H., Philippidou P., Surmeli G., Grinstein J. and Dasen J. S. (2013). Genetic and functional modularity of Hox activities in the specification of limb-innervating motor neurons. *PLoS Genet.* 9, e1003184 10.1371/journal.pgen.1003184 23359544 PMC3554521

[DEV194761C40] Lanfear R. (2010). Are the deuterostome posterior Hox genes a fast-evolving class? *Adv. Exp. Med. Biol.* 689, 111-122. 10.1007/978-1-4419-6673-5_8 20795326

[DEV194761C41] Langmead B., Trapnell C., Pop M. and Salzberg S. L. (2009). Ultrafast and memory-efficient alignment of short DNA sequences to the human genome. *Genome Biol.* 10, R25 10.1186/gb-2009-10-3-r25 19261174 PMC2690996

[DEV194761C42] Lewis E. B. (1978). A gene complex controlling segmentation in Drosophila. *Nature* 276, 565-570. 10.1038/276565a0 103000

[DEV194761C43] Liao Y., Smyth G. K. and Shi W. (2019). The R package Rsubread is easier, faster, cheaper and better for alignment and quantification of RNA sequencing reads. *Nucleic Acids Res.* 47, e47 10.1093/nar/gkz114 30783653 PMC6486549

[DEV194761C44] Lippmann E. S., Williams C. E., Ruhl D. A., Estevez-Silva M. C., Chapman E. R., Coon J. J. and Ashton R. S. (2015). Deterministic HOX patterning in human pluripotent stem cell-derived neuroectoderm. *Stem Cell Rep.* 4, 632-644. 10.1016/j.stemcr.2015.02.018 PMC4400649 25843047

[DEV194761C45] Love M. I., Huber W. and Anders S. (2014). Moderated estimation of fold change and dispersion for RNA-seq data with DESeq2. *Genome Biol.* 15, 550 10.1186/s13059-014-0550-8 25516281 PMC4302049

[DEV194761C46] Machado C. B., Kanning K. C., Kreis P., Stevenson D., Crossley M., Nowak M., Iacovino M., Kyba M., Chambers D., Blanc E. et al. (2014). Reconstruction of phrenic neuron identity in embryonic stem cell-derived motor neurons. *Development* 141, 784-794. 10.1242/dev.097188 24496616 PMC3912827

[DEV194761C47] Machanick P. and Bailey T. L. (2011). MEME-ChIP: motif analysis of large DNA datasets. *Bioinformatics* 27, 1696-1697. 10.1093/bioinformatics/btr189 21486936 PMC3106185

[DEV194761C48] Mahony S. and Benos P. V. (2007). STAMP: a web tool for exploring DNA-binding motif similarities. *Nucleic Acids Res.* 35, W253-W258. 10.1093/nar/gkm272 17478497 PMC1933206

[DEV194761C49] Mahony S., Edwards M. D., Mazzoni E. O., Sherwood R. I., Kakumanu A., Morrison C. A., Wichterle H. and Gifford D. K. (2014). An integrated model of multiple-condition ChIP-Seq data reveals predeterminants of Cdx2 binding. *PLoS Comput. Biol.* 10, e1003501 10.1371/journal.pcbi.1003501 24675637 PMC3967921

[DEV194761C50] Mann R. S. and Affolter M. (1998). Hox proteins meet more partners. *Curr. Opin. Genet. Dev.* 8, 423-429. 10.1016/S0959-437X(98)80113-5 9729718

[DEV194761C51] Mann R. S. and Chan S.-K. (1996). Extra specificity from extradenticle: the partnership between HOX and PBX/EXD homeodomain proteins. *Trends Genet.* 12, 258-262. 10.1016/0168-9525(96)10026-3 8763497

[DEV194761C52] Mann R. S., Lelli K. M. and Joshi R. (2009). Hox specificity unique roles for cofactors and collaborators. *Curr. Top. Dev. Biol.* 88, 63-101. 10.1016/S0070-2153(09)88003-4 19651302 PMC2810641

[DEV194761C53] Mathelier A., Fornes O., Arenillas D. J., Chen C.-Y., Denay G., Lee J., Shi W., Shyr C., Tan G., Worsley-Hunt R. et al. (2016). JASPAR 2016: a major expansion and update of the open-access database of transcription factor binding profiles. *Nucleic Acids Res.* 44, D110-D115. 10.1093/nar/gkv1176 26531826 PMC4702842

[DEV194761C54] Mazzoni E. O., Mahony S., Iacovino M., Morrison C. A., Mountoufaris G., Closser M., Whyte W. A., Young R. A., Kyba M., Gifford D. K. et al. (2011). Embryonic stem cell-based mapping of developmental transcriptional programs. *Nat. Methods* 8, 1056-1058. 10.1038/nmeth.1775 22081127 PMC3228994

[DEV194761C55] Mazzoni E. O., Mahony S., Peljto M., Patel T., Thornton S. R., Mccuine S., Reeder C., Boyer L. A., Young R. A., Gifford D. K. et al. (2013). Saltatory remodeling of Hox chromatin in response to rostrocaudal patterning signals. *Nat. Neurosci.* 16, 1191-1198. 10.1038/nn.3490 23955559 PMC3799941

[DEV194761C56] McGinnis W. and Krumlauf R. (1992). Homeobox genes and axial patterning. *Cell* 68, 283-302. 10.1016/0092-8674(92)90471-N 1346368

[DEV194761C57] Mclean C. Y., Bristor D., Hiller M., Clarke S. L., Schaar B. T., Lowe C. B., Wenger A. M. and Bejerano G. (2010). GREAT improves functional interpretation of cis-regulatory regions. *Nat. Biotechnol.* 28, 495-501. 10.1038/nbt.1630 20436461 PMC4840234

[DEV194761C58] Merabet S. and Mann R. S. (2016). To be specific or not: the critical relationship between Hox and TALE proteins. *Trends Genet.* 32, 334-347. 10.1016/j.tig.2016.03.004 27066866 PMC4875764

[DEV194761C59] Metzis V., Steinhauser S., Pakanavicius E., Gouti M., Stamataki D., Ivanovitch K., Watson T., Rayon T., Mousavy Gharavy S. N., Lovell-Badge R. et al. (2018). Nervous system regionalization entails axial allocation before neural differentiation. *Cell* 175, 1105-1118.e17. 10.1016/j.cell.2018.09.040 30343898 PMC6218657

[DEV194761C60] Narendra V., Rocha P. P., An D., Raviram R., Skok J. A., Mazzoni E. O. and Reinberg D. (2015). CTCF establishes discrete functional chromatin domains at the Hox clusters during differentiation. *Science* 347, 1017-1021. 10.1126/science.1262088 25722416 PMC4428148

[DEV194761C61] Narendra V., Bulajić M., Dekker J., Mazzoni E. O. and Reinberg D. (2016). CTCF-mediated topological boundaries during development foster appropriate gene regulation. *Genes Dev.* 30, 2657-2662. 10.1101/gad.288324.116 28087711 PMC5238725

[DEV194761C62] Noyes M. B., Christensen R. G., Wakabayashi A., Stormo G. D., Brodsky M. H. and Wolfe S. A. (2008). Analysis of homeodomain specificities allows the family-wide prediction of preferred recognition sites. *Cell* 133, 1277-1289. 10.1016/j.cell.2008.05.023 18585360 PMC2478728

[DEV194761C63] Peljto M. and Wichterle H. (2011). Programming embryonic stem cells to neuronal subtypes. *Curr. Opin. Neurobiol.* 21, 43-51. 10.1016/j.conb.2010.09.012 20970319 PMC3050008

[DEV194761C64] Peljto M., Dasen J. S., Mazzoni E. O., Jessell T. M. and Wichterle H. (2010). Functional diversity of ESC-derived motor neuron subtypes revealed through intraspinal transplantation. *Cell Stem Cell* 7, 355-366. 10.1016/j.stem.2010.07.013 20804971 PMC2933095

[DEV194761C65] Porcelli D., Fischer B., Russell S. and White R. (2019). Chromatin accessibility plays a key role in selective targeting of Hox proteins. *Genome Biol.* 20, 115 10.1186/s13059-019-1721-4 31159833 PMC6547607

[DEV194761C66] Regulski M., Harding K., Kostriken R., Karch F., Levine M. and McGinnis W. (1985). Homeo box genes of the Antennapedia and bithorax complexes of Drosophila. *Cell* 43, 71-80. 10.1016/0092-8674(85)90013-3 2416463

[DEV194761C67] Rhee H. S., Closser M., Guo Y., Bashkirova E. V., Tan G. C., Gifford D. K. and Wichterle H. (2016). Expression of terminal effector genes in mammalian neurons is maintained by a dynamic relay of transient enhancers. *Neuron* 92, 1252-1265. 10.1016/j.neuron.2016.11.037 27939581 PMC5193225

[DEV194761C68] Robinson M. D., Mccarthy D. J. and Smyth G. K. (2010). edgeR: a Bioconductor package for differential expression analysis of digital gene expression data. *Bioinformatics* 26, 139-140. 10.1093/bioinformatics/btp616 19910308 PMC2796818

[DEV194761C69] Rousso D. L., Gaber Z. B., Wellik D., Morrisey E. E. and Novitch B. G. (2008). Coordinated actions of the forkhead protein Foxp1 and Hox proteins in the columnar organization of spinal motor neurons. *Neuron* 59, 226-240. 10.1016/j.neuron.2008.06.025 18667151 PMC2547125

[DEV194761C70] Slattery M., Riley T., Liu P., Abe N., Gomez-Alcala P., Dror I., Zhou T., Rohs R., Honig B., Bussemaker H. J. et al. (2011). Cofactor binding evokes latent differences in DNA binding specificity between Hox proteins. *Cell* 147, 1270-1282. 10.1016/j.cell.2011.10.053 22153072 PMC3319069

[DEV194761C71] Srivastava D., Aydin B., Mazzoni E. O. and Mahony S. (2020). An interpretable bimodal neural network characterizes the sequence and preexisting chromatin predictors of induced TF binding. *bioRxiv*, 672790 10.1101/672790 PMC7788824 33413545

[DEV194761C72] Sweeney L. B., Bikoff J. B., Gabitto M. I., Brenner-Morton S., Baek M., Yang J. H., Tabak E. G., Dasen J. S., Kintner C. R. and Jessell T. M. (2018). Origin and segmental diversity of spinal inhibitory interneurons. *Neuron* 97, 341-355.e3. 10.1016/j.neuron.2017.12.029 29307712 PMC5880537

[DEV194761C73] Tan G. C., Mazzoni E. O. and Wichterle H. (2016). Iterative role of Notch signaling in spinal motor neuron diversification. *Cell Rep.* 16, 907-916. 10.1016/j.celrep.2016.06.067 27425621 PMC4975379

[DEV194761C74] Welch R. P., Lee C., Imbriano P. M., Patil S., Weymouth T. E., Smith R. A., Scott L. J. and Sartor M. A. (2014). ChIP-Enrich: gene set enrichment testing for ChIP-seq data. *Nucleic Acids Res.* 42, e105 10.1093/nar/gku463 24878920 PMC4117744

[DEV194761C75] Wichterle H. and Peljto M. (2008). Differentiation of mouse embryonic stem cells to spinal motor neurons. *Curr. Protoc. Stem Cell Biol.* 5, 1H.1.1-1H.1.9. 10.1002/9780470151808.sc01h01s5 18770634

[DEV194761C76] Wichterle H., Lieberam I., Porter J. A. and Jessell T. M. (2002). Directed differentiation of embryonic stem cells into motor neurons. *Cell* 110, 385-397. 10.1016/S0092-8674(02)00835-8 12176325

[DEV194761C77] Wu Y., Wang G., Scott S. A. and Capecchi M. R. (2008). Hoxc10 and Hoxd10 regulate mouse columnar, divisional and motor pool identity of lumbar motoneurons. *Development* 135, 171-182. 10.1242/dev.009225 18065432

[DEV194761C78] Young T., Rowland J. E., van de Ven C., Bialecka M., Novoa A., Carapuco M., van Nes J., de Graaff W., Duluc I., Freund J.-N. et al. (2009). Cdx and Hox genes differentially regulate posterior axial growth in mammalian embryos. *Dev. Cell* 17, 516-526. 10.1016/j.devcel.2009.08.010 19853565

[DEV194761C79] Zaret K. S. and Carroll J. S. (2011). Pioneer transcription factors: establishing competence for gene expression. *Genes Dev.* 25, 2227-2241. 10.1101/gad.176826.111 22056668 PMC3219227

[DEV194761C80] Zeiske T., Baburajendran N., Kaczynska A., Brasch J., Palmer A. G. III, Shapiro L., Honig B. and Mann R. S. (2018). Intrinsic DNA shape accounts for affinity differences between hox-cofactor binding sites. *Cell Rep.* 24, 2221-2230. 10.1016/j.celrep.2018.07.100 30157419 PMC6166240

