# Supplementary material for: Differential abilities to engage inaccessible chromatin diversify vertebrate Hox binding patterns
Source: Development. 2020 Nov 23;147(22):dev194761. doi: 10.1242/dev.194761 (PMC7710020; doi:10.1242/dev.194761)
Supplement: Supplementary information [file develop-147-194761-s1.pdf]

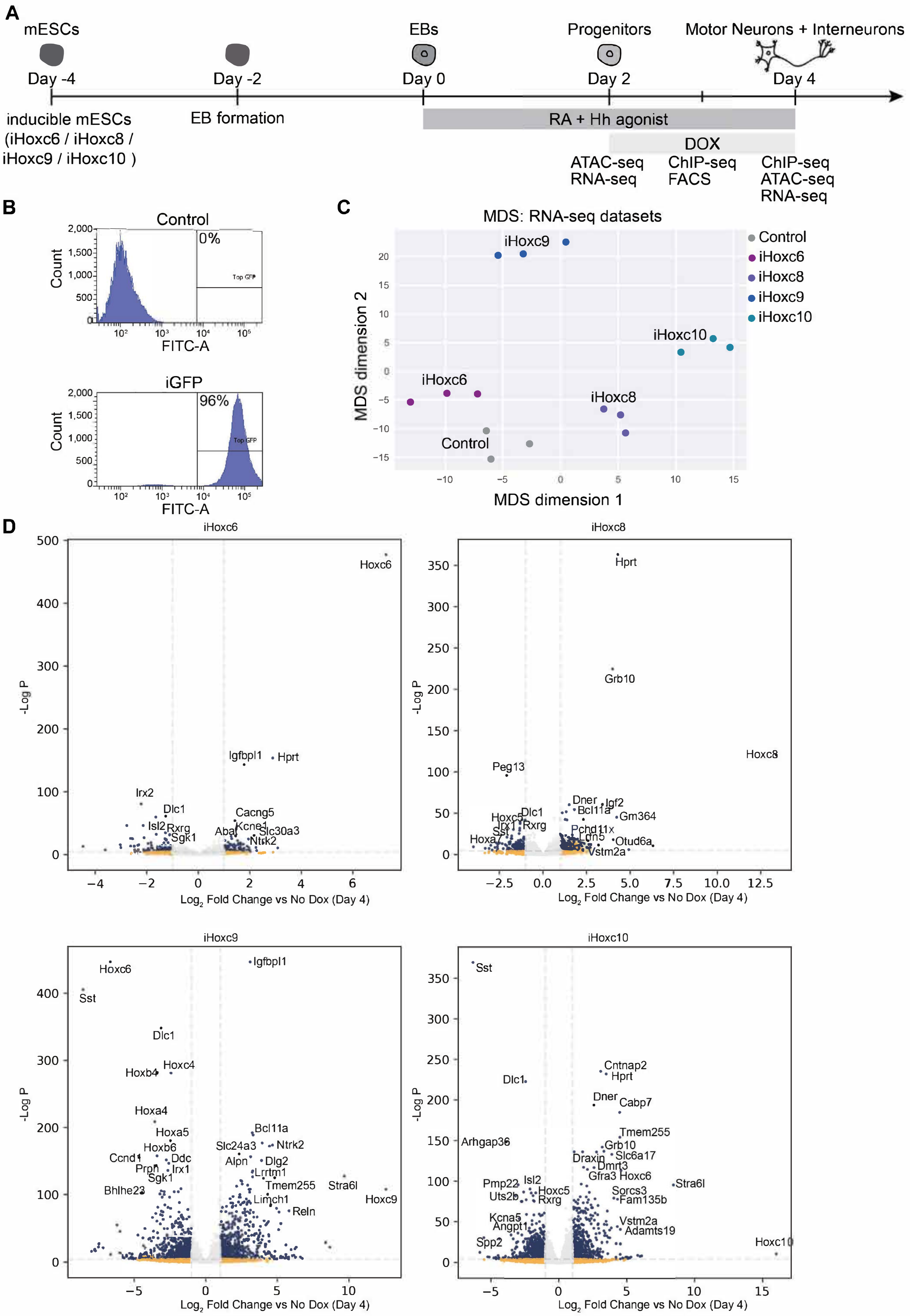

Development • Supplementary information

Fig. S1: HOX TFs induce distinct gene expression profiles.

(A) Overview of the experimental procedure. ESCs differentiate into MNs and interneurons in response to RA and Hedgehog patterning signals. Expression of inducible genes was induced by treating cells with Dox. Cells were collected at distinct time points for RNA-seq, ChIP-seq, ATAC-seq and FACS analysis. (mESCs: mouse embryonic stem cells. EBs: embryoid bodies) (B) FACS analysis of a no Dox control and inducible GFP (iGFP) line on day 3 (iGFP line treated with Dox for 24h prior to FACS). Percentage of GFP+ cells indicated on the graph (n=1 independent differentiation). (C) Multidimensional scaling (MDS) of the RNA-seq datasets (day 4) reveals similarities in the gene expression profiles induced by HOX TFs (each dot represents independent differentiations). (D) Volcano plots showing changes in gene expression in iHoxc6, iHoxc8, iHoxc9 and iHoxc10 relative to no Dox control neurons (day 4) (n=3 independent differentiations).

Development • Supplementary information

A

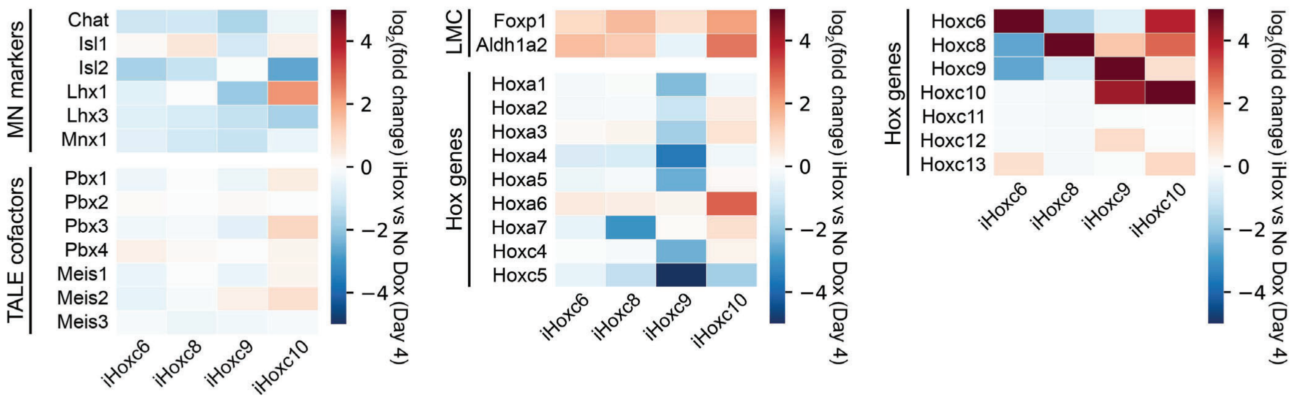

B

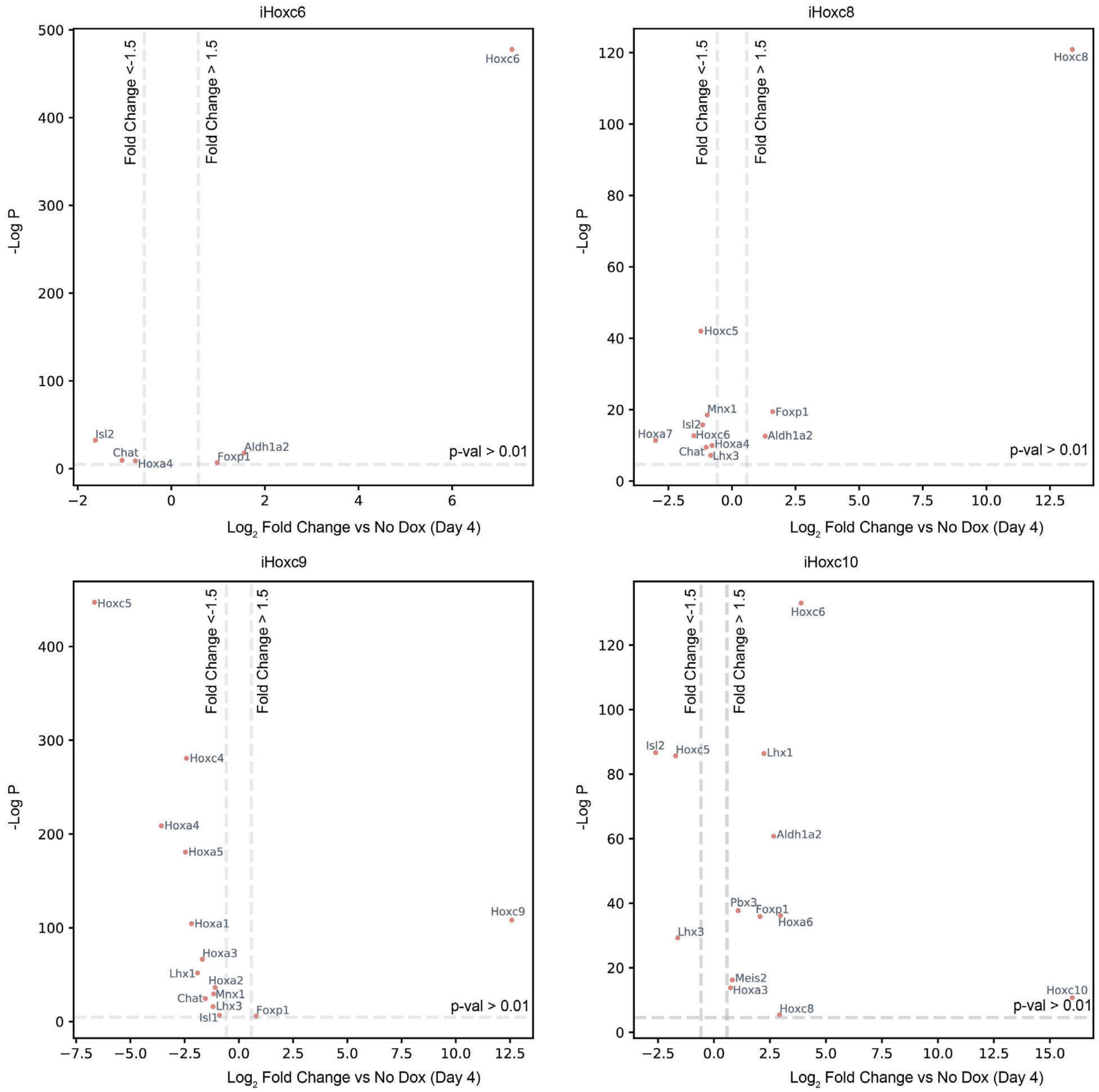

C

Fragments per Kilobase Per Million (FPKM), Gene Lengths calculated using Rsubread

Gene	pMN_r1	pMN_r2	pMN_r3	noDox_r1	noDox_r2	noDox_r3	iHoxc6_r1	iHoxc6_r2	iHoxc6_r3	iHoxc8_r1	iHoxc8_r2	iHoxc8_r3	iHoxc9_r1	iHoxc9_r2	iHoxc9_r3	iHoxc10_r1	iHoxc10_r2	iHoxc10_r3
Hoxc5	15.89	25.41	26.02	161.40	126.28	128.51	98.21	138.92	80.36	53.01	71.50	54.40	1.41	1.87	0.81	48.66	36.91	42.81
Hoxc6	0.11	0.17	0.24	3.28	1.58	1.16	324.27	303.89	314.14	0.97	0.46	0.71	1.21	1.44	1.32	31.21	31.58	27.34
Hoxc8	0.06	0.17	0.03	0.01	0.00	0.03	0.00	0.00	0.00	165.47	171.09	148.19	0.07	0.01	0.05	0.15	0.12	0.07
Hoxc9	0.06	0.08	0.02	0.02	0.02	0.02	0.00	0.00	0.00	0.02	0.02	0.00	128.21	134.77	121.64	0.04	0.02	0.05
Hoxc10	0.00	0.00	0.00	0.00	0.00	0.00	0.00	0.00	0.00	0.00	0.00	0.00	0.00	0.18	0.02	227.18	226.11	228.20

UTR	pMN_r1	pMN_r2	pMN_r3	noDox_r1	noDox_r2	noDox_r3	iHoxc6_r1	iHoxc6_r2	iHoxc6_r3	iHoxc8_r1	iHoxc8_r2	iHoxc8_r3	iHoxc9_r1	iHoxc9_r2	iHoxc9_r3	iHoxc10_r1	iHoxc10_r2	iHoxc10_r3
Hoxc5 UTR	10.37	10.59	14.75	72.43	61.86	69.76	50.06	49.06	47.23	27.94	34.32	25.02	0.73	0.76	0.50	20.26	22.09	22.48
Hoxc6 UTR	0.12	0.27	0.26	2.26	0.60	0.57	1.39	1.17	1.40	0.90	0.38	0.70	1.32	1.52	1.16	27.72	30.53	25.23
Hoxc8 UTR	0.09	0.09	0.03	0.07	0.00	0.00	0.00	0.00	0.00	14.50	13.73	14.73	0.12	0.13	0.11	0.03	0.13	0.06
Hoxc9 UTR	0.02	0.06	0.02	0.02	0.02	0.02	0.00	0.00	0.00	0.02	0.00	0.00	0.02	0.02	0.05	0.02	0.04	0.05
Hoxc10 UTR	0.00	0.00	0.00	0.00	0.00	0.00	0.00	0.00	0.00	0.00	0.00	0.00	0.00	0.00	0.00	0.04	0.04	0.09

Development • Supplementary information

Fig. S2: HOX TFs induce the expected markers during *in vitro* spinal cord differentiation.

(A) RNA–seq heatmap showing the expression of representative genes in iHoxc6, iHoxc8, iHoxc9 and iHoxc10 relative to no Dox control neurons (day 4) (n=3 independent differentiations). (B) Plots showing significantly differentially expressed representative genes from Fig. S2A. (C) Normalized (FPKM) read counts for Hox genes and their UTRs in the indicated conditions (in 3 independent differentiations) (pMN: progenitors (Day 2)).

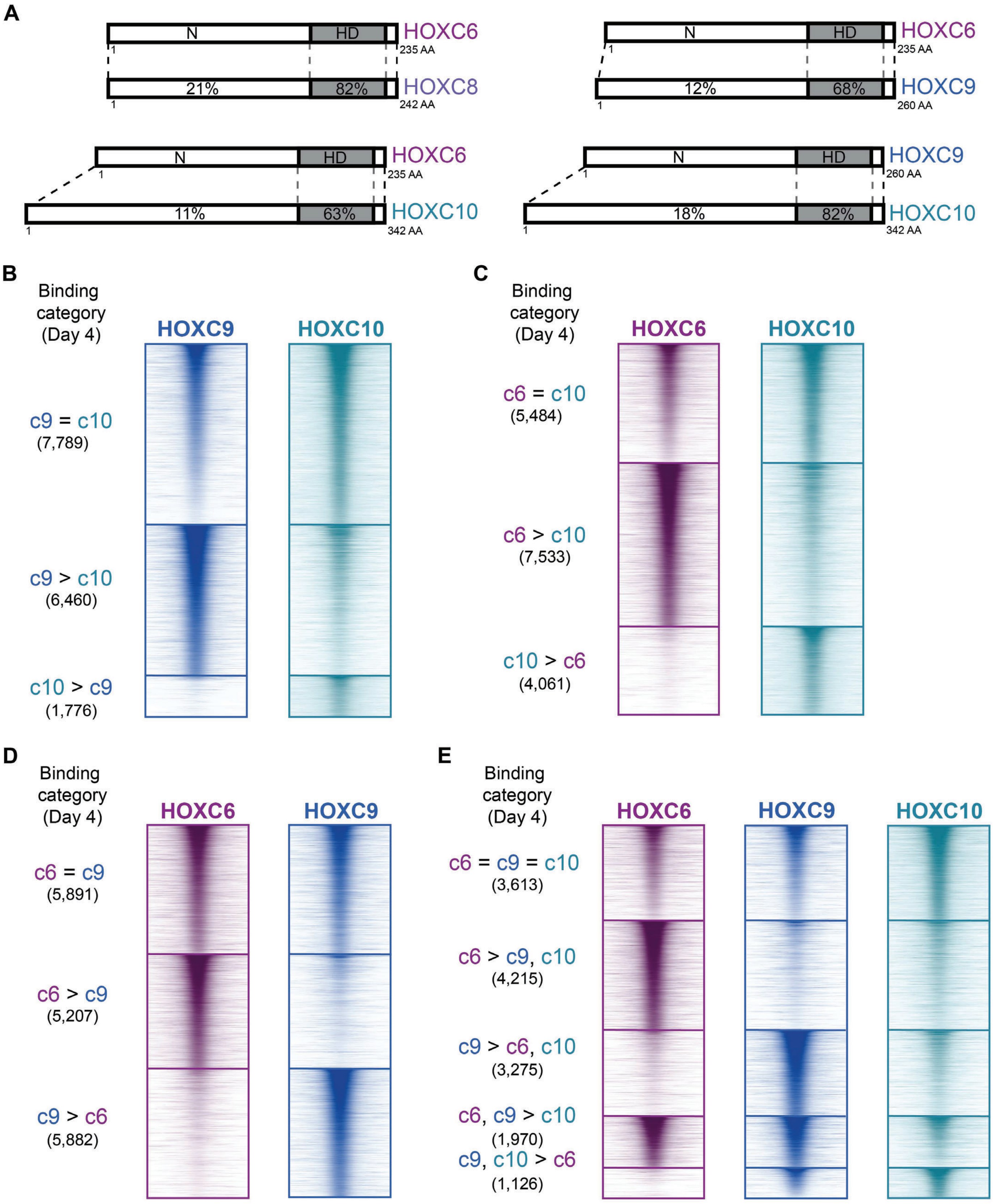

Development • Supplementary information

Fig. S3: HOXC6, HOXC9 and HOXC10 TFs have different genome-wide binding profiles at Day 4.

(A) Schematic indicating the percentage of conserved amino acids in the homeodomain (HD) and the rest of the protein between the indicated HOX TFs. (B-D) ChIP-seq heatmap showing binding comparisons in differentiating neurons at day 4 (n=2 independent differentiations). Sites bound by both indicated HOX TFs are indicated as ‘=’ sites. Preferentially bound sites by HOXC6, HOXC9 or HOXC10 are indicated as ‘c6 >’, ‘c9 >’, and ‘c10 >’, respectively. (E) ChIP-seq heatmap showing binding comparisons of HOXC6, HOXC9 and HOXC10 in differentiating neurons at day 4 (n=2 independent differentiations). Sites bound by all three HOX TFs are indicated as ‘c6 = c9 = c10’ sites. Preferentially bound sites by HOXC6, HOXC9, HOXC6 and HOXC9 or HOXC9 and HOXC10 are indicated as ‘c6 > c9, c10’, ‘c9 > c6, c10’, ‘c6, c9 > c10’, and ‘c9, c10 > c6’, respectively.

Development • Supplementary information

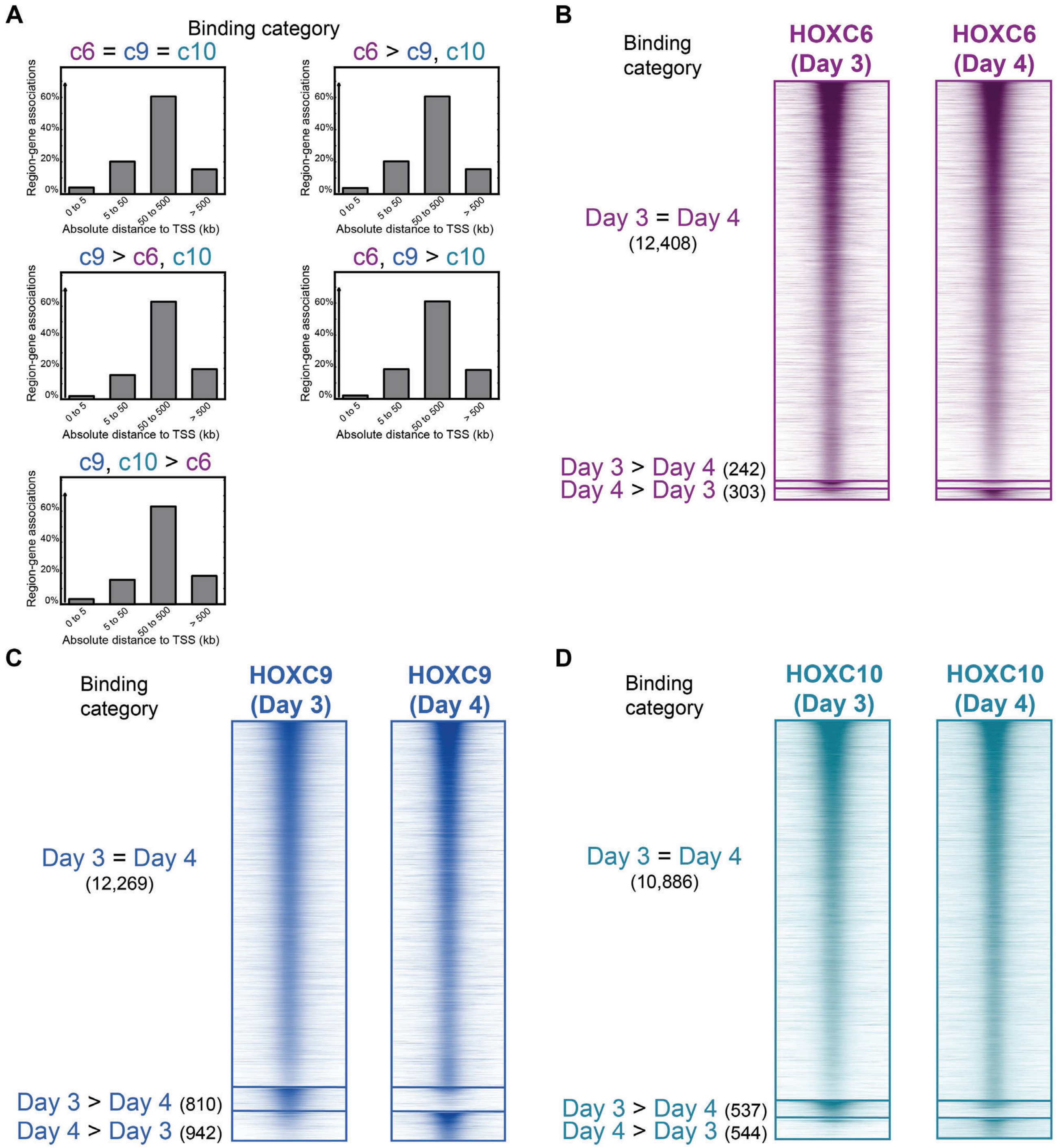

Fig. S4: HOXC6, HOXC9 and HOXC10 TFs have largely the same binding patterns at Day 3 vs Day 4.

(A) Region-gene association graphs at the indicated HOX binding categories from Fig. 2E. (B-D) ChIP-seq heatmaps showing binding comparisons of the indicated HOX TFs at day 3 versus day 4 (n=2 independent differentiations). Sites equally bound at both time points noted as ‘Day 3 = Day 4’ sites. Differentially bound sites noted as ‘Day 3 > Day 4’ and ‘Day 4 > Day 3’.

Development • Supplementary information

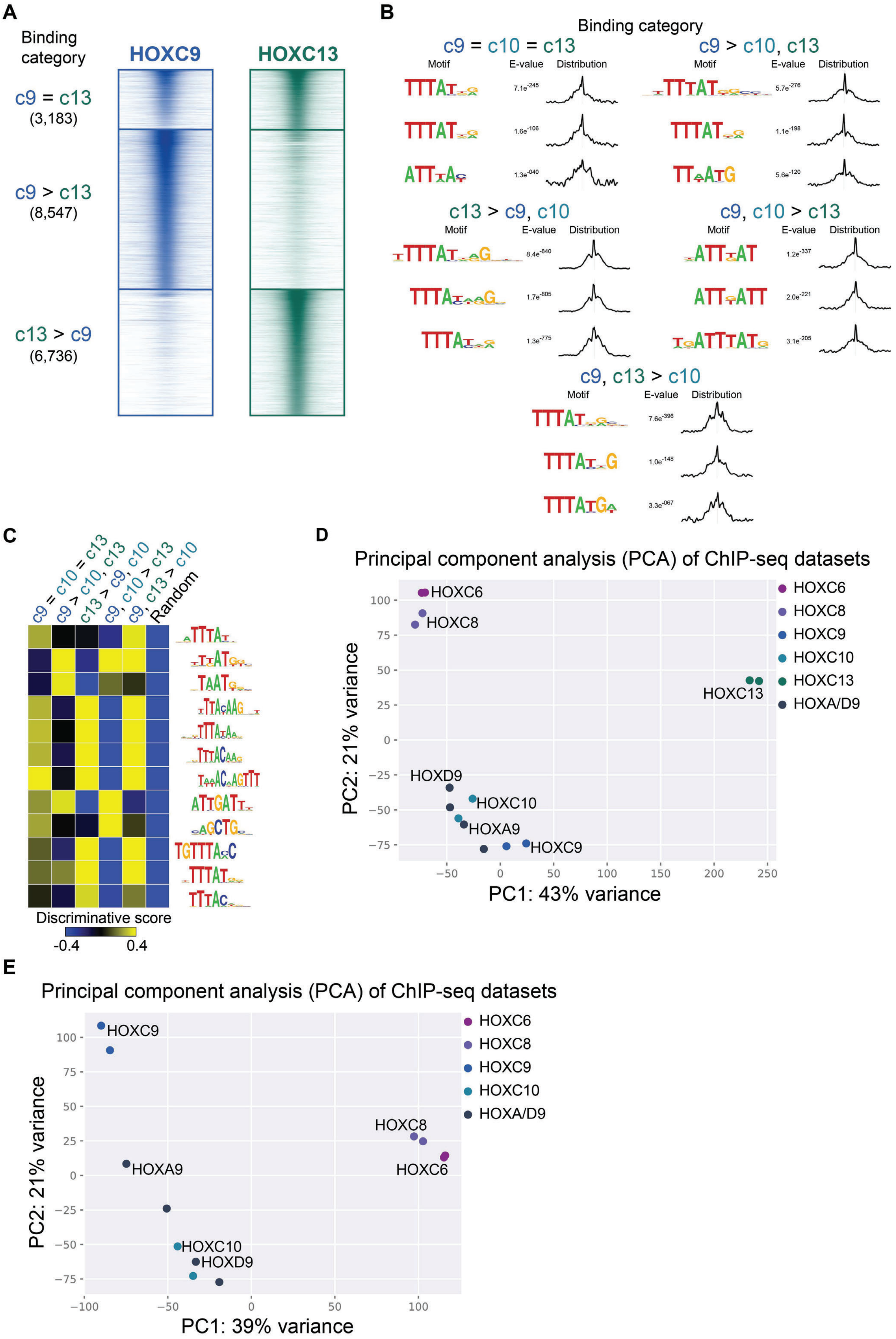

Development • Supplementary information

Fig. S5: HOXC9 and HOXC13 genome-wide binding profiles differ from other posterior HOX TFs.

(A) ChIP-seq heatmap showing binding comparisons of HOXC9 and HOXC13 in differentiating neurons at day 3 (n=2 independent differentiations). Sites bound by both indicated HOX TFs noted as ‘c9 = c13’ sites. Preferentially bound sites by HOXC9 or HOXC13 noted as ‘c9 > c13’ or ‘c13 > c9’. (B) Selected top enriched motifs discovered via MEME-ChIP at the indicated HOX binding categories from Fig. 7A. Distributions to the right of each motif show the distribution of each motif occurrence with respect to the midpoint of each peak (500bp windows). (C) SeqUnwinder analysis characterizing motifs that are discriminative between the various classes of HOXC9, HOXC10 and HOXC13 binding sites from Fig. 7A. (D-E) Principal Component Analysis (PCA) of the ChIP-seq datasets reveals similarities in the binding patterns of HOX TFs (each dot represents independent differentiations).

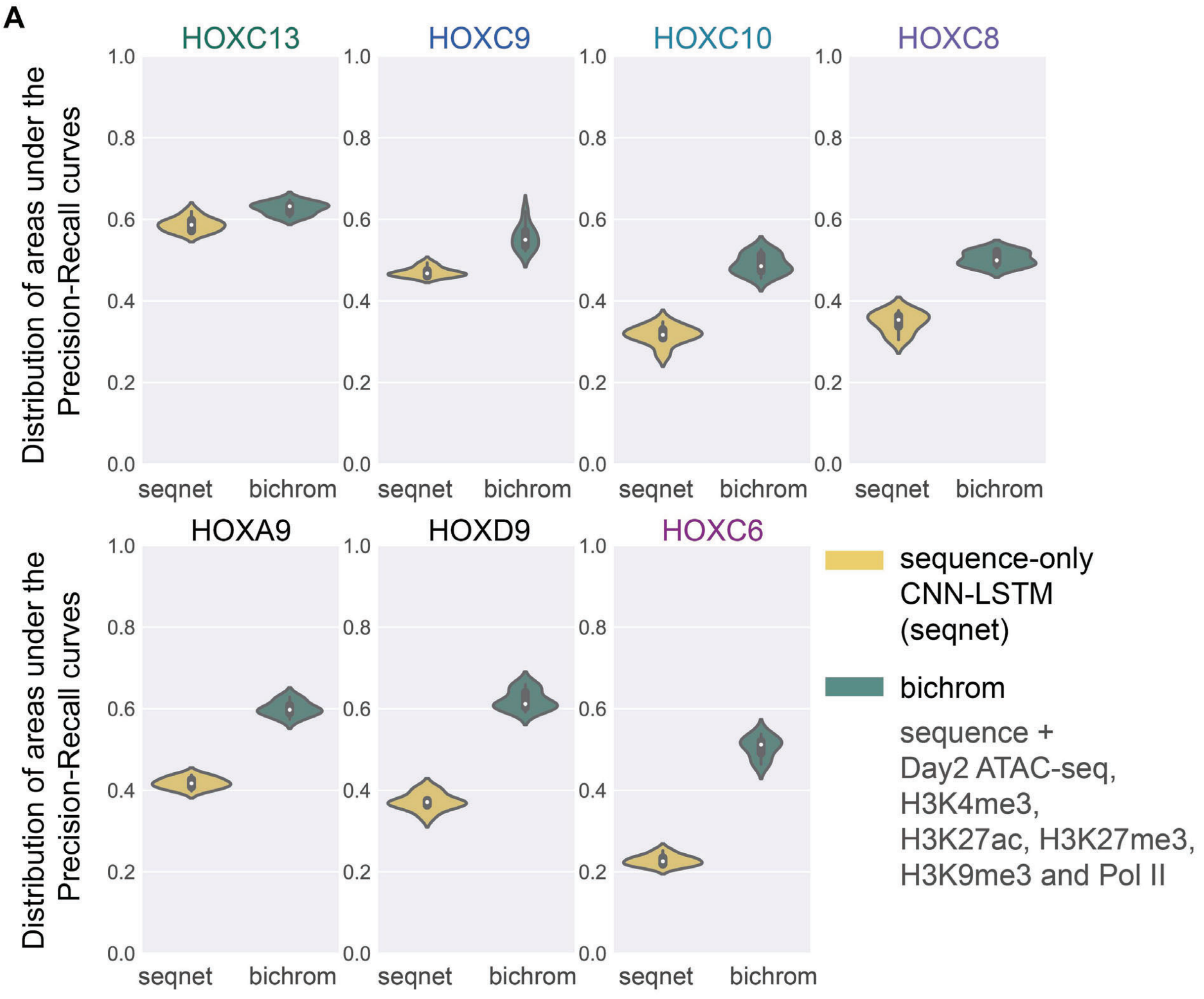

Fig. S6: Neural network-based analysis quantifies HOX TFs' relative dependence on prior chromatin states.

(A) The distribution of areas under the precision-recall curve (auPRCs) for 9 iterations of a CNN-LSTM sequence-only network (seqnet, yellow) compared to a bimodal network that incorporates both sequence and day 2 (progenitor) chromatin data to predict genome-wide induced HOX TF binding (Bichrom, green). The chromatin data used includes day 2 ATAC-seq and ChIP-seq for H3K4me3, H3K27ac, H3K27me3, H3K9me3 and Pol II. For each TF, both seqnet and Bichrom are trained using 9 distinct training sets, each corresponding to an independent held out test-set. The auPRC distributions are represented using violin plots.

Development • Supplementary information

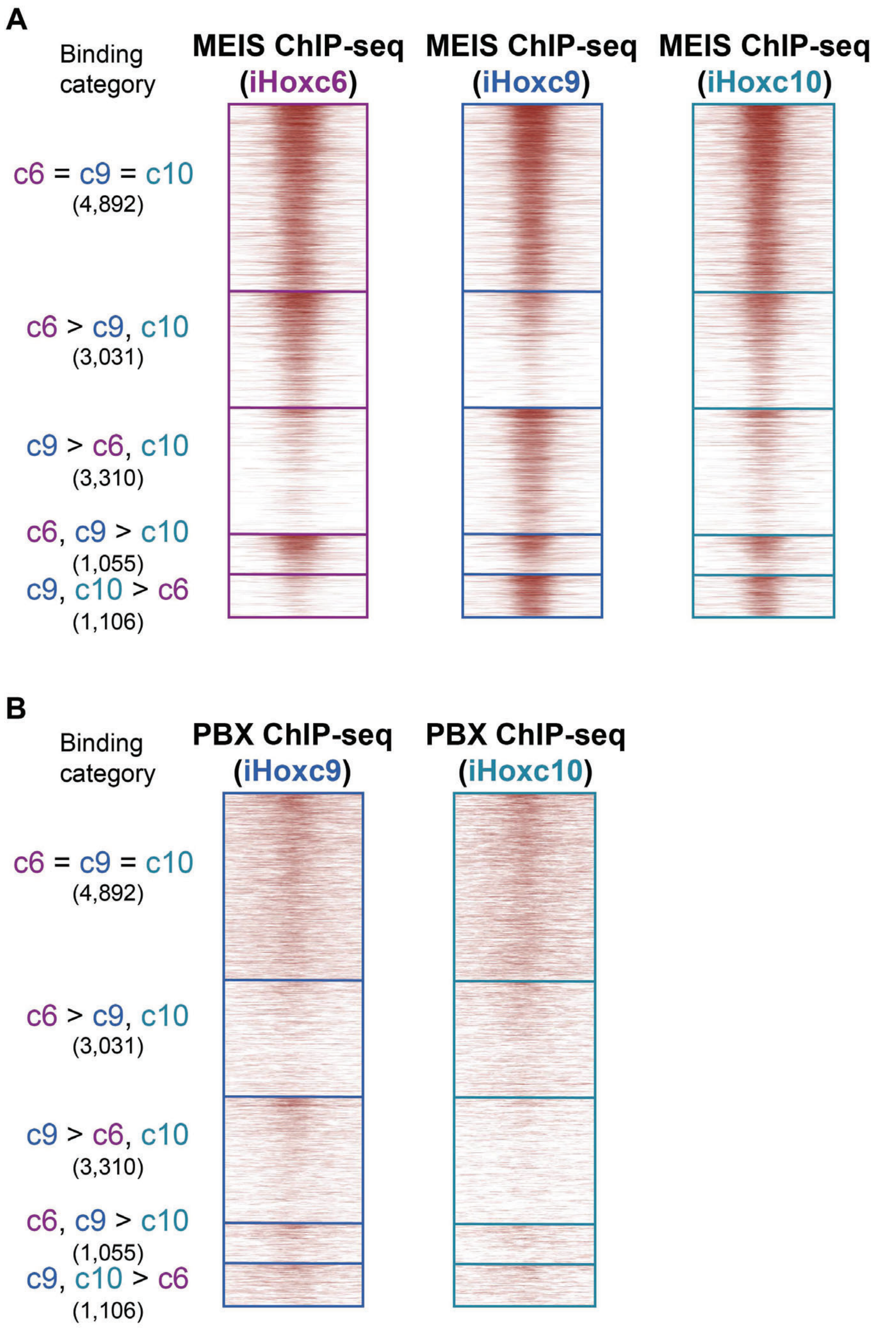

Fig. S7: Overlap of TALE cofactor binding with HOXC6, HOXC9 and HOXC10.

(A-B) ChIP-seq of MEIS and PBX performed in the indicated cell line reveals co-binding with the respective HOX TF at the indicated HOX binding categories from Fig. 2E (n=1 independent differentiation).
