# Supplementary material for: Differential abilities to engage inaccessible chromatin diversify vertebrate Hox binding patterns
Source: Development. 2020 Nov 23;147(22):dev194761. doi: 10.1242/dev.194761 (PMC7710020; doi:10.1242/dev.194761)
Supplement: Reviewer comments [file develop-147-194761_review_history.pdf]

Differential abilities to engage inaccessible chromatin diversify vertebrate Hox binding patterns

Milica Bulajić, Divyanshi Srivastava, Jeremy S. Dasen, Hynek Wichterle, Shaun Mahony and Esteban O. Mazzoni
DOI: 10.1242/dev.194761

Editor: James Briscoe

Review timeline
Original submission: 6 July 2020
Editorial decision: 28 July 2020
First revision received: 9 September 2020
Accepted: 25 September 2020

Original submission

First decision letter

MS ID#: DEVELOP/2020/194761

MS TITLE: Differential abilities to engage inaccessible chromatin diversify vertebrate HOX binding patterns

AUTHORS: Milica Bulajic, Divyanshi Srivastava, Jeremy Dasen, Hynek Wichterle, Shaun Mahony, and Esteban Mazzoni

I have now received all the referees' reports on the above manuscript, and have reached a decision. The referees' comments are appended below, or you can access them online: please go to BenchPress and click on the 'Manuscripts with Decisions' queue in the Author Area.

As you will see, the referees express considerable interest in your work, but have some comments and questions that will need to be addressed before we can consider publication. Addressing these comments should be straightforward and will clarify and strengthen the conclusions of your study.If you are able to revise the manuscript along the lines suggested, I will be happy receive a revised version of the manuscript. Your revised paper will be re-reviewed by one or more of the original referees, and acceptance of your manuscript will depend on your addressing satisfactorily the reviewers' major concerns. Please also note that Development will normally permit only one round of major revision.

We are aware that you may currently be unable to access the lab to undertake experimental revisions. If it would be helpful, we encourage you to contact us to discuss your revision in greater detail. Please send us a point-by-point response indicating where you are able to address concerns raised (either experimentally or by changes to the text) and where you will not be able to do so within the normal timeframe of a revision. We will then provide further guidance. Please also note that we are happy to extend revision timeframes as necessary.

Please attend to all of the reviewers' comments and ensure that you clearly highlight all changes made in the revised manuscript. Please avoid using 'Tracked changes' in Word files as these are lost in PDF conversion. I should be grateful if you would also provide a point-by-point response detailing how you have dealt with the points raised by the reviewers in the 'Response to Reviewers' box. If you do not agree with any of their criticisms or suggestions please explain clearly why this is so.

© 2020. Published by The Company of Biologists under the terms of the Creative Commons Attribution License (https://creativecommons.org/licenses/by/4.0/).

Reviewer 1

Advance summary and potential significance to field

see below

Comments for the author

The manuscript by Bulajic et al. is concerned with the analysis of HOX protein binding, with the aim of understanding how differential binding can influence the fates of motoneurons (MN). The authors focus on a subset of HOX proteins (C6, 8, 9 and C10) due to their intrinsic capacities to induce various AP-related neuronal identities within the developing spinal cord. The work is carried out in a well-controlled in vitro (ES) differentiation system and all four proteins are flagged with the same epitope to better compare results. ChIP-seq of the four tagged proteins at different time points reveals important differences between the binding of HOXC9 and HOXC10, despite similarities in the binding sites, unlike HOXC6 and HOXC8. They also show that the binding profiles of central HOX proteins is different from that of AbdB-related proteins.

When binding sites are examined, sequence divergence cannot explain the differential binding between HOXC9 and HOX10 (both show an over-representation of TTTAT core sequence) and hence the binding of HOXC9 and HOXC10 cannot be associated with any obvious difference in DNA sequence. When looking at ATAC-seq results in progenitors, it appears that HOXC9 binding sites are the least accessible. This 'preference' of HOXC9 for 'inaccessible' chromatin sites is also observed in an independent cell line, where the chromatin landscape is different from MN progenitors, strengthening the argument. The authors conclude that not all HOX proteins have the same capacity to bind 'closed' chromatin and that HOXC9 stands out in this respect. Further analyses reveal that other group 9 protein display the same capacity as well as group 13 proteins, which even have the strongest capacity to act as pioneer proteins, as reported recently by the Kmita and Drouin laboratories.

Altogether I find the work very well done and of substantial general interest. The authors use an in vitro system, but it is well characterized and it was previously shown to faithfully reflect the in vivo processes at work. The major conclusion, which is that HOX proteins differential binding can in some cases be explained by their capacities to access closed chromatin, and that posterior proteins are better at this than anterior ones (Hox13 in particular), is certainly worth publishing in Development. The fact that a potential 'pioneer' effect of HOX proteins was recently reported (Desanlis et al., 2020 and Amandio et al. 2020) does not detract at all from the interest of this paper. I would even say that it is essential that different datasets leading to convergent conclusions are being published in visible journals such as to make the point stronger and definitive. Accordingly, I recommend this paper for publication in Development with no reservation and only minor modifications.

Major concern:
Figure 8B: I do have a slight problem with panel B of figure 8. As it stands, it shows that HOXC13 can bind to regions that are not yet labelled with ATAC-seq, and this with a wider range than HOXC9. It also shows that most sites that can be 'opened' by HOXC9' (i.e. within the K27me3 domain) are also opened by HOXC13 (and not vice versa). This is a good piece of evidence, yet I do have two questions:

1) Did the authors check that ALL chromatin is polycomb positive, i.e. that there isn't any cellular heterogeneity with some cells devoid of K27me3 at this locus and hence that the differential binding could still be due to different DNA sequences rather than to chromatin accessibility? For example, would the authors have a corresponding K27 acetylation profile? The ATAC-seq profile clearly indicates that the cluster is indeed more 'open' in the anterior part, but there is some background in the posterior part too. Is this a concern?

2) More importantly, the way the figure is presented suggests that HOXC13 could be used to open the chromatin at the HOXC cluster and hence be responsible for the colinear activation. But this is an artificial HOXC13 protein and one can see on the profile that the endogenous HOXC13 gene is expectedly not yet active (K27me3 and no ATAC-seq signal on the homeobox exon). While I

© 2020. Published by The Company of Biologists under the terms of the Creative Commons Attribution License (https://creativecommons.org/licenses/by/4.0/).

guess this is obvious for the authors it is a bit confusing for the Hoxologists involved in cluster-wide regulation. This could be solved by mentioning that the HOXC cluster is used in this panel as an example of closed chromatin being challenged by HOX proteins, but that any other genomic loci may have been used to substantiate this argument in fact and that this does not mean to reflect the situation occurring in the embryo at this cluster (i.e. at a time when no HOX13 proteins are present yet..).

Minors:
*Page 3, line 16: The original reference (if necessary) to the expansion of AbdB genes in vertebrates is Ispizua-Belmonte et al. 1991, EMBO J.
*Page 4, line 1: two brakets..))
*Page 4, end, and Page 12, five lines from bottom: I do not think that these references should be given in one single ‘package’. Mallo, Deschamps and Pourquie’s contributions were gain of function approaces carried out with large (uncontrolled) amounts of exogenous proteins. The exact mechanism described by Bulajic et al. in this submitted paper precisely explains why drastic phenotypes could be obtained when HOX13 proteins are delivered in excess, due to their pioneering activities and hence why it is absolutely necessary to deal with physiological (close to-) amounts of exogenous proteins whenever gain of function approaches are used.
In contrast, Capecchi’s work involved physiological conditions (loss of function) and the phenotype, while significant, was much less severe. It involved a gain of ca. 2 caudal vertebra… And a similar phenotype was NOT observed neither with the Hoxd13 KO, nor with the HoxC13 KO mice (combinations thereof were difficult to produce for other reasons). Therefore, the statement on the termination of the body, while potentially correct, could be tone down a little bit.
*While the potential pioneer effect of HOX13 proteins was shown genome wide by Desanlis et al., (2020), a documented example was reported by Amandio et al. eLife, (2020; Figure 8) on the opening of a physiologically important enhancer sequence by HOX13 protein.
*Finally, I was wondering why the authors never mention the term ‘pioneer factor’? If there is a ‘biological’ reason (i.e. if they think that it does not deserve this qualification because of a precise reason), why not mentioning it in the discussion?

Reviewer 2

Advance summary and potential significance to field

The manuscript by Bulajic et al. reports on the analysis of HoxC differential binding during in vitro motor neuron differentiation. Through the comparative analysis of HoxC6, 8, 9, 10 and 13, the authors convincingly show that the differential binding of the HoxC transcription factors cannot be simply explained by differences in DNA sequence preference. Interestingly, they found that these HoxC TFs have distinct abilities to bind to inaccessible chromatin. Moreover, they reveal that following induction of individual HoxC TF, HoxC9 and HoxC13 have a marked preference for inaccessible chromatin compared to the other HoxC TFs tested. Finally, they show that HoxC9 and HoxC13 induction results in increase chromatin accessibility at their targets.

While previous evidence pointed to Hox TFs having the ability to trigger chromatin accessibility both in flies and mice, the finding making the present work of particular significance is that this property varies among the HoxC TFs. This difference is observed not only for the different HoxC TFs but also among members of the same paralogous group (namely paralogous group 9), which is particularly interesting knowing the partial functional overlap often observed between Hox paralogs even when these paralogs have highly similar expression patterns.

In summary, this work provides important new information regarding Hox functional properties and their associated developmental processes, which should be of interest to the developmental biology community.

Comments for the author
Minor comment:
The authors may want to elaborate on the possible significance of the variation in Hox-dependent chromatin accessibility in the context of posterior prevalence as well as the distinct functional outcomes of Hox TFs belonging to the same paralogous group.

© 2020. Published by The Company of Biologists under the terms of the Creative Commons Attribution License (https://creativecommons.org/licenses/by/4.0/).

Reviewer 3

Advance summary and potential significance to field

In this report, Bilajic et al tackle an important developmental biology question, namely how posterior Hox transcription factors (TFs) bind the genome to install a specific rostro-caudal positional identity on spinal cord cells during embryonic development.
To overcome the limitations of studying Hox binding in vivo they switch to an in vitro system based on the directed differentiation of mouse embryonic stem cells (mESCs) to ventral spinal cord neurons. To study Hox binding, they have generated inducible cell lines for Hoxc6, Hoxc8, Hoxc9 and Hoxc10 (iHox) that are important for the establishment of a brachial, thoracic and lumbar spinal cord identity. Each iHox mESC line is differentiated to ventral spinal cord neurons through the addition of retinoic acid that is inducing a neural anterior spinal cord identity (Hox5) and shh that is important for ventral identity. The authors induce the expression of the specific Hox TF at the neural progenitor state by the addition of doxycycline to the cell culture medium.
Their data reveal that Hoxc6 and Hoxc10, which are important for limb innervating motor neurons (MNs), they do not bind the genome with identical patterns. They show that this is mainly caused due to their differential ability to bind motifs that are occluded in inaccessible chromatin. They present evidence that Hoxc9 has a greater ability to bind inaccessible chromatin compared to other posterior Hox TFs and that this is also the case in comparison to other Hox genes belonging to the same paralogue group. They finally propose a model where the different ability of Hox TFs to bind inaccessible chromatin is what drives the patterning diversification during development.

Comments for the author

The authors are experts in the field of HOX regulation and have produced seminal work in the field. The differentiation of mouse embryonic stem cells to MNs by H. Wichterle had been a key advancement. However it is clear that using this protocol the MNs acquire mostly hindbrain and/or anterior spinal cord identity (up to Hox5). Thus the main concern is that the authors study Hox TF binding activity in the context of previously established in vitro cellular chromatin landscapes that correspond to hindbrain or cervical spinal cord and not more posterior identities. In order to extrapolate the results and have some valid conclusions that also apply to the embryo it is of critical importance that the in vitro environment recapitulates as much as possible the one found in vivo. Recent studies by Metzis et al, Cell, 2018 have shown that posteriorization of epiblast cells occurs before neural induction and that the chromatin landscape of posterior progenitor cells expressing Cdx2, is completely different to the one found in anterior neural progenitors. These recent findings by Metzis et al, are also in agreement with the findings published by Mazzoni et al, Nat Neuroscience, 2013. There the authors showed that remodelling of Hox chromatin in response to WNT/FGF signals induces the expression of Cdx2, which is important for the opening of the Hox5-9 cluster and emphasized the importance of using the correct patterning signals to induce chromatin remodelling early on.
Additionally, it has been shown by other groups that addition of WNT/FGF at an early time point of differentiation induces the formation of neuromesodermal progenitors (summarized in Henrique et al 2014), which are important for the generation of posterior spinal cord neurons of brachial, thoracic and lumbar identity (Gouti et al, 2014, Lippmann et al, 2015). Collectively, all these findings come in agreement with the distinct developmental origin of posterior spinal cord cells and nicely explain why it has not been possible to generate more posterior neurons using the Wichterle protocol. The authors will have at least to discuss this literature in their discussion and highlight the caveat in their study. In the future it will be necessary to assess the chromatin landscape and binding of Hox TFs under conditions that allow the activation of the endogenous posterior Hox genes. This will provide conclusive evidence and give a final answer to the important question of how specific spinal cord identities are established in the spinal cord.

Specific points
- How efficient is the induction of iHox genes using the tet-on system. What is the percentage of cells that express the specific Hox gene?
- The authors show that Hoxc9 overexpression represses more anterior Hox genes (Hox1-5). What is the situation with the other Hox genes (Hoxc6, Hoxc8 and Hoxc10)? How the authors explain that they don't repress more anterior Hox genes ?

© 2020. Published by The Company of Biologists under the terms of the Creative Commons Attribution License (https://creativecommons.org/licenses/by/4.0/).

- In the iHoxc10 line the expression of Hoxa6 and Hoxc6 is very high. This is unexpected and the question arises if this is physiologically relevant. Is this caused by the high levels of transgene expression? Can the authors check in vivo using chick in ovo electroporation if this will be the case?
- Is the expression of a specific HOX TF sufficient to install the specific positional identity? Are the endogenous genes activated?
- It has been previously shown by Dasen et al, Nature, 2003 that over expression of Hoxc9 in the brachial neural tube of chick embryos is sufficient to repress Hoxc6 expression and induce autonomic MN identity.

Thus, one would expect that PGC identity is induced in the iHoxc9 line. Have the authors looked into the expression of Bmp5, nNOS and pSMAD associated with PGCs?

First revision

Author response to reviewers' comments

We are deeply thankful to the Editors, Staff and Reviewers for their effort and work during these challenging times. We hope you are well and healthy.

Note: Reviewers comments

Authors comments

Reviewer 1 Advance Summary and Potential Significance to Field:
Reviewer 1 Comments for the Author:
The manuscript by Bulajic et al. is concerned with the analysis of HOX protein binding, with the aim of understanding how differential binding can influence the fates of motoneurons (MN). The authors focus on a subset of HOX proteins (C6, 8, 9 and C10) due to their intrinsic capacities to induce various AP-related neuronal identities within the developing spinal cord. The work is carried out in a well-controlled in vitro (ES) differentiation system and all four proteins are flagged with the same epitope to better compare results.

ChIP-seq of the four tagged proteins at different time points reveals important differences between the binding of HOXC9 and HOXC10, despite similarities in the binding sites, unlike HOXC6 and HOXC8. They also show that the binding profiles of central HOX proteins is different from that of AbdB-related proteins.

When binding sites are examined, sequence divergence cannot explain the differential binding between HOXC9 and HOX10 (both show an over-representation of TTTAT core sequence) and hence the binding of HOXC9 and HOXC10 cannot be associated with any obvious difference in DNA sequence. When looking at ATAC-seq results in progenitors, it appears that HOXC9 binding sites are the least accessible. This ‘preference’ of HOXC9 for ‘inaccessible’ chromatin sites is also observed in an independent cell line, where the chromatin landscape is different from MN progenitors, strengthening the argument. The authors conclude that not all HOX proteins have the same capacity to bind ‘closed’ chromatin and that HOXC9 stands out in this respect.
Further analyses reveal that other group 9 protein display the same capacity as well as group 13 proteins, which even have the strongest capacity to act as pioneer proteins, as reported recently by the Kmita and Drouin laboratories.

Altogether I find the work very well done and of substantial general interest. The authors use an in vitro system, but it is well characterized and it was previously shown to faithfully reflect the in vivo processes at work. The major conclusion, which is that HOX proteins differential binding can in some cases be explained by their capacities to access closed chromatin, and that posterior proteins are better at this than anterior ones (Hox13 in particular), is certainly worth publishing in Development. The fact that a potential ‘pioneer’ effect of HOX proteins was recently reported (Desanlis et al., 2020 and Amandio et al. 2020) does not detract at all from the interest of this

© 2020. Published by The Company of Biologists under the terms of the Creative Commons Attribution License (https://creativecommons.org/licenses/by/4.0/).

paper. I would even say that it is essential that different datasets leading to convergent conclusions are being published in visible journals such as to make the point stronger and definitive. Accordingly, I recommend this paper for publication in Development with no reservation and only minor modifications.

We would like to thank the Reviewer for taking the time to evaluate the manuscript critically and for the positive comments about our work.

During the review process, we finish what we think is an important experiment that deepens the manuscript’s main message. There are two possible explanations for differential pioneer behavior across the posterior *Hox* group:

1) Changes in the homeodomain sequence that do not affect transcription factor sequence preference but change pioneer abilities. 2) Pioneering function resides in the N-terminus, outside of the homeodomain.

To discriminate between these two possibilities, we introduce a new set of experiments characterizing chimeric posterior HOX TF binding. We chose HOXC10 and HOXC13 because of their low and high ability to bind inaccessible chromatin, respectively. We found that the homeodomain and C-terminus of the protein control HOX TF ability to bind to inaccessible chromatin (Reviewer Fig. 1) (New Manuscript Fig. 8C-E). This alternative will force the transcription factor and developmental biology fields to look at homeodomains beyond DNA-sequence preference, with the additional pioneer dimension.

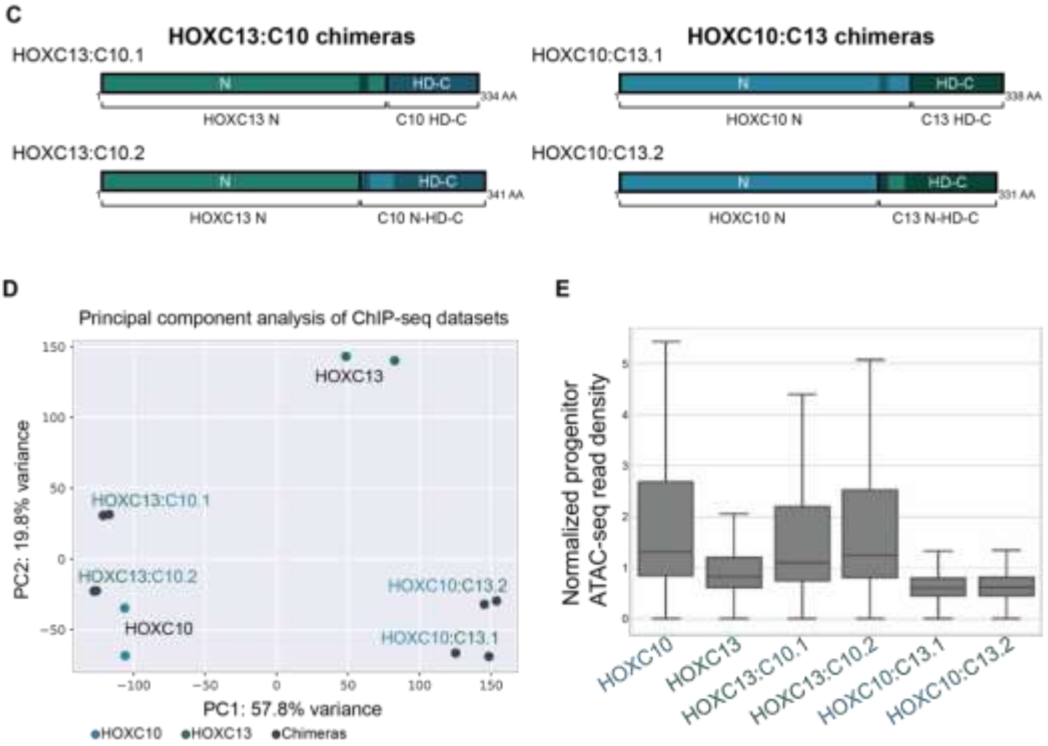

Reviewer Fig. 1 (New Manuscript Fig. 8C-E): (C) Schematic describing HOXC13:C10 and HOXC10:C13 chimeric HOX proteins. HD: Homeodomain. (D) Principal Component Analysis (PCA) of the ChIP-seq datasets reveals similarities in the binding patterns of HOX TFs. (E) The distribution of Day 2 progenitor ATAC-seq read density at the top 10,000 binding sites of the indicated HOX TF at Day 3. Data is ordered based on normalized read density (tags per million per site) and divided into quartiles. The box displays the central 50% (quartile 2 and quartile 3) of the data, while the top and bottom whiskers represent the top 25% and bottom 25% (the top and the bottom quartiles) of the data, respectively.

Major concern:
Figure 8B: I do have a slight problem with panel B of figure 8. As it stands, it shows that HOXC13

© 2020. Published by The Company of Biologists under the terms of the Creative Commons Attribution License (https://creativecommons.org/licenses/by/4.0/).

can bind to regions that are not yet labelled with ATAC-seq, and this with a wider range than HOXC9. It also shows that most sites that can be 'opened' by HOXC9' (i.e. within the K27me3 domain) are also opened by HOXC13 (and not vice versa). This is a good piece of evidence, yet I do have two questions:

1) Did the authors check that ALL chromatin is polycomb positive, i.e. that there isn't any cellular heterogeneity with some cells devoid of K27me3 at this locus and hence that the differential binding could still be due to different DNA sequences rather than to chromatin accessibility? For example, would the authors have a corresponding K27 acetylation profile? The ATAC-seq profile clearly indicates that the cluster is indeed more 'open' in the anterior part, but there is some background in the posterior part too. Is this a concern?

This point stems from our desire to show a differential binding example for all HOX TFs in a single locus. Also, we know that this piece of information would be potentially interesting to other labs working on *Hox* regulation. But thanks to the Reviewer, we see how it can generate some confusion.

We did not mean to give the impression that HOXC9 binding sites are more associated with H3K27me3. In fact, we confirmed there is no preference for H3K27me3. For all HOX TFs, less than 1% of the top 10,000 binding sites overlap preexisting H3K27me3. However, the caveat here is that only 3,795 domains were called genome wide for H3K27me3 in Day 2 progenitor motor neurons (PMID: 23955559), thereby making it difficult to draw meaningful conclusions from an overlap analysis.

In addition, we also analyzed the overlap with preexisting H3K27ac. H3K27ac is depleted at HOXC9 and HOXC13 sites when compared to HOXC6, HOXC8, HOXC10, and HOXA/D9 (Reviewer Table 1). This difference mirrors the differential prior accessibility at their binding sites, enforcing HOXC9 and HOXC13's ability to engage less active regulatory elements.

TF	HOXC6	HOXC8	HOXC9	HOXC10	HOXC13	HOXA9	HOXD9
Overlap	23.3	18.3	14.3	22.9	8.15	24.4	28.7

Reviewer Table 1: Percentage overlap of H3K27ac with the top 10,000 HOX TF binding sites.

Hox genes are bivalent in pluripotent cells, and thus contain some accessibility signal even in undifferentiated embryonic stem cells. As differentiation progresses, activated *Hox* genes gain accessibility while posterior repressed *Hox* genes lose some. The remaining accessibility is particularly evident at transcription start sites, as shown in Figure 8B. Thus, we did not find the small accessibility peaks at posterior *Hox* genes worrisome.

We included Day 2 progenitor H3K27ac in Figure 8B, confirming that there is no background enrichment of H3K27ac in the posterior part of the *HoxC* cluster (Reviewer Fig. 2) (modified Manuscript Fig. 8B).

© 2020. Published by The Company of Biologists under the terms of the Creative Commons Attribution License (https://creativecommons.org/licenses/by/4.0/).

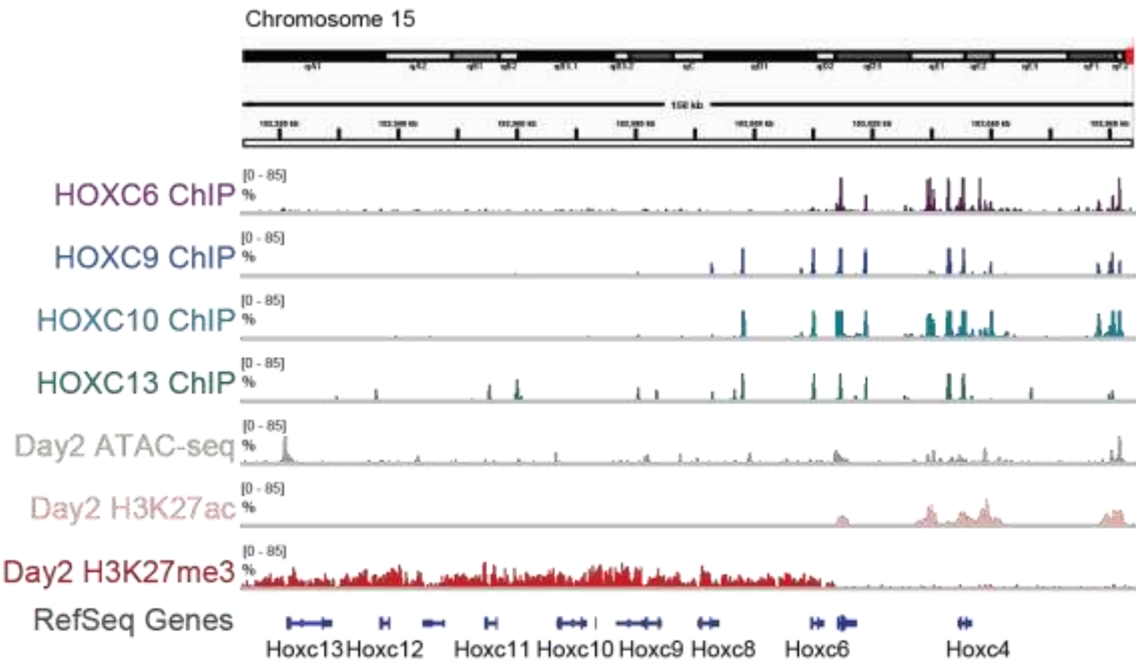

Reviewer Fig. 2 (modified Manuscript Fig. 8B): Browser screenshots of the indicated HOX ChIP-seqs, Day 2 ATAC-seq, H3K27ac and H3K27me3 ChIP-seqs at the *HoxC* gene cluster. All tracks are rescaled to 85th percentile. H3K27ac and H3K27me3 ChIP-seq datasets previously published in PMID: 27939581 and PMID: 23955559, respectively.

Finally, chromatin states in single cells, and even each allele, is an exciting question that requires single-cell technologies for chromatin immunoprecipitation experiments. Although we are trying to set up single-cell H3K27me3, we do not have it ready yet. Thus, we cannot address this issue experimentally yet.

2) More importantly, the way the figure is presented suggests that HOXC13 could be used to open the chromatin at the HOXC cluster and hence be responsible for the colinear activation. But this is an artificial HOXC13 protein and one can see on the profile that the endogenous HOXC13 gene is expectedly not yet active (K27me3 and no ATAC-seq signal on the homeobox exon). While I guess this is obvious for the authors, it is a bit confusing for the Hoxologists involved in cluster-wide regulation. This could be solved by mentioning that the HOXC cluster is used in this panel as an example of closed chromatin being challenged by HOX proteins, but that any other genomic loci may have been used to substantiate this argument in fact, and that this does not mean to reflect the situation occurring in the embryo at this cluster (i.e. at a time when no HOX13 proteins are present yet..).
The Reviewer is correct, and we now see how it will confuse readers. We now clearly state that our visualization of the *Hox* cluster here is used mostly as an example only possible in the *in vitro* system or gain- of-function experiments and does not reflect the situation in the embryo.

Minors:
*Page 3, line 16: The original reference (if necessary) to the expansion of AbdB genes in vertebrates is Ispizua- Belmonte et al. 1991, EMBO J.

Thank you for the reference, we added it to the revised manuscript.

*Page 4, line 1: two brakets..))

Thank you for such attention to detail, we meant to note the relevant reviews by stating: (reviewed in (Duboule, 2007, Lanfear, 2010)).

*Page 4, end, and Page 12, five lines from bottom: I do not think that these references should be

© 2020. Published by The Company of Biologists under the terms of the Creative Commons Attribution License (https://creativecommons.org/licenses/by/4.0/).

given in one single ‘package’. Mallo, Deschamps and Pourquie’s contributions were gain of function approaches carried out with large (uncontrolled) amounts of exogenous proteins. The exact mechanism described by Bulajic et al. in this submitted paper precisely explains why drastic phenotypes could be obtained when HOX13 proteins are delivered in excess, due to their pioneering activities and hence why it is absolutely necessary to deal with physiological (close to-) amounts of exogenous proteins whenever gain of function approaches are used.
In contrast, Capecchi’s work involved physiological conditions (loss of function) and the phenotype, while significant, was much less severe. It involved a gain of ca. 2 caudal vertebra… And a similar phenotype was NOT observed neither with the Hoxd13 KO, nor with the HoxC13 KO mice (combinations thereof were difficult to produce for other reasons). Therefore, the statement on the termination of the body, while potentially correct, could be tone down a little bit.

This is an excellent suggestion that help us to put the ectopic *Hoxc13* expression into context. We made the appropriate changes to the text to reflect the literature.

*While the potential pioneer effect of HOX13 proteins was shown genome wide by Desanlis et al., (2020), a documented example was reported by Amandio et al. eLife, (2020; Figure 8) on the opening of a physiologically important enhancer sequence by HOX13 protein.

Thank you for pointing at the right reference. We added Amandio et al. eLife, (2020) in the revised manuscript.

*Finally, I was wondering why the authors never mention the term ‘pioneer factor’? If there is a ‘biological’ reason (i.e. if they think that it does not deserve this qualification because of a precise reason), why not mentioning it in the discussion?

The Reviewer is correct in noticing our lack of usage of the term ‘pioneer factor’. We refrained from using it because many, including the coiner of the term K. Zaret, define “pioneer TFs” as those that prefer to bind DNA wrapped on nucleosomes. Although our work clearly shows that some HOX TFs can bind to inaccessible chromatin and increase accessibility after binding, we do not conclusively demonstrate that HOX TFs bind directly to nucleosomal DNA. Thus, we are reluctant to use the term “pioneer” in case it suggests a particular mechanism to some readers. However, we recognize that the functional outcome of HOXC9/HOXC13 binding is similar to that of classic pioneer TFs (i.e., previously inaccessible chromatin becomes accessible). Perhaps there is a need for a new TF classification. We added a note in the discussion to elaborate on this matter.

Reviewer 2 Advance Summary and Potential Significance to Field:
The manuscript by Bulajic et al. reports on the analysis of HoxC differential binding during in vitro motor neuron differentiation. Through the comparative analysis of HoxC6, 8, 9, 10 and 13, the authors convincingly show that the differential binding of the HoxC transcription factors cannot be simply explained by differences in DNA sequence preference. Interestingly, they found that these HoxC TFs have distinct abilities to bind to inaccessible chromatin. Moreover, they reveal that following induction of individual HoxC TF, HoxC9 and HoxC13 have a marked preference for inaccessible chromatin compared to the other HoxC TFs tested. Finally, they show that HoxC9 and HoxC13 induction results in increase chromatin accessibility at their targets.

While previous evidence pointed to Hox TFs having the ability to trigger chromatin accessibility both in flies and mice, the finding making the present work of particular significance is that this property varies among the HoxC TFs. This difference is observed not only for the different HoxC TFs but also among members of the same paralogous group (namely paralogous group 9), which is particularly interesting knowing the partial functional overlap often observed between Hox paralogs even when these paralogs have highly similar expression patterns.

In summary, this work provides important new information regarding Hox functional properties and their associated developmental processes, which should be of interest to the developmental biology community.

Thank you Reviewer 2, for taking the time to review our manuscript and the enthusiastic reaction to our work.

© 2020. Published by The Company of Biologists under the terms of the Creative Commons Attribution License (https://creativecommons.org/licenses/by/4.0/).

Reviewer 2 Comments for the Author: Minor comment:
The authors may want to elaborate on the possible significance of the variation in Hox-dependent chromatin accessibility in the context of posterior prevalence as well as the distinct functional outcomes of Hox TFs belonging to the same paralogous group.
We added a section about the significance of our results in the context of posterior prevalence and paralog groups in the discussion.
During the review process, we finish what we think is an important experiment that deepens the manuscript's main message. There are two possible explanations for differential pioneer behavior across the posterior *Hox* group:
1) Changes in the homeodomain sequence that do not affect transcription factor sequence preference but change pioneer abilities. 2) Pioneering function resides in the N-terminus, outside of the homeodomain.
To discriminate between these two possibilities, we introduce a new set of experiments characterizing chimeric posterior HOX TF binding. We chose HOXC10 and HOXC13 because of their low and high ability to bind inaccessible chromatin, respectively. We found that the homeodomain and C-terminus of the protein control HOX TF ability to bind to inaccessible chromatin (Reviewer Fig. 1) (New Manuscript Fig. 8C-E). This alternative will force the transcription factor and developmental biology fields to look at homeodomains beyond DNA-sequence preference, with the additional pioneer dimension.

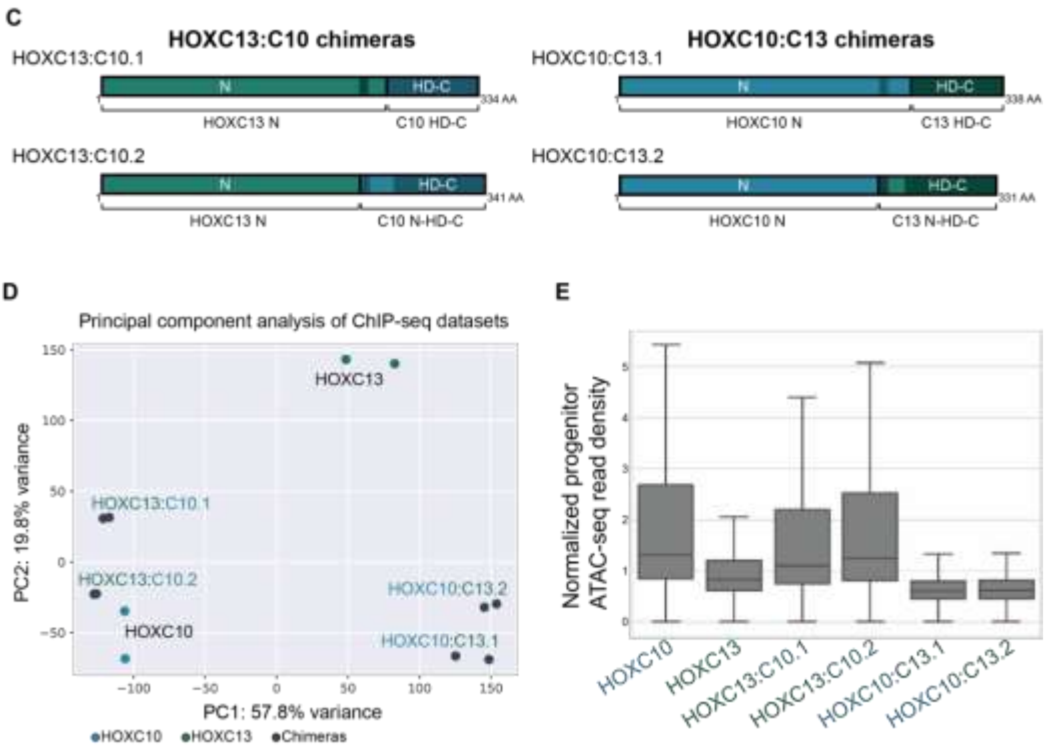

Reviewer Fig. 1 (New Manuscript Fig. 8C-E): (C) Schematic describing HOXC13:C10 and HOXC10:C13 chimeric HOX proteins. HD: Homeodomain. (D) Principal Component Analysis (PCA) of the ChIP-seq datasets reveals similarities in the binding patterns of HOX TFs. (E) The distribution of Day 2 progenitor ATAC-seq read density at the top 10,000 binding sites of the indicated HOX TF at Day 3. Data is ordered based on normalized read density (tags per million per site) and divided into quartiles. The box displays the central 50% (quartile 2 and quartile 3) of the data, while the top and bottom whiskers represent the top 25% and bottom 25% (the top and the bottom quartiles) of the data, respectively.

Reviewer 3 Advance Summary and Potential Significance to Field:
In this report, Bilajic et al tackle an important developmental biology question, namely how posterior Hox transcription factors (TFs) bind the genome to install a specific rostro-caudal positional identity on spinal cord cells during embryonic development.
To overcome the limitations of studying Hox binding in vivo they switch to an in vitro system based

© 2020. Published by The Company of Biologists under the terms of the Creative Commons Attribution License (https://creativecommons.org/licenses/by/4.0/).

on the directed differentiation of mouse embryonic stem cells (mESCs) to ventral spinal cord neurons. To study Hox binding, they have generated inducible cell lines for Hoxc6, Hoxc8, Hoxc9 and Hoxc10 (iHox) that are important for the establishment of a brachial, thoracic and lumbar spinal cord identity. Each iHox mESC line is differentiated to ventral spinal cord neurons through the addition of retinoic acid that is inducing a neural anterior spinal cord identity (Hox5) and shh that is important for ventral identity. The authors induce the expression of the specific Hox TF at the neural progenitor state by the addition of doxycycline to the cell culture medium.

Their data reveal that Hoxc6 and Hoxc10, which are important for limb innervating motor neurons (MNs), they do not bind the genome with identical patterns. They show that this is mainly caused due to their differential ability to bind motifs that are occluded in inaccessible chromatin. They present evidence that Hoxc9 has a greater ability to bind inaccessible chromatin compared to other posterior Hox TFs and that this is also the case in comparison to other Hox genes belonging to the same paralogue group. They finally propose a model where the different ability of Hox TFs to bind inaccessible chromatin is what drives the patterning diversification during development.

We would like to thank the Reviewer 3 as well for taking the time to read and critically evaluate the manuscript. We appreciate the Reviewer noticing both the strengths and weaknesses of our manuscript.

During the review process, we finish what we think is an important experiment that deepens the manuscript's main message. There are two possible explanations for differential pioneer behavior across the posterior *Hox* group:
1) Changes in the homeodomain sequence that do not affect transcription factor sequence preference but change pioneer abilities. 2) Pioneering function resides in the N-terminus, outside of the homeodomain.
To discriminate between these two possibilities, we introduce a new set of experiments characterizing chimeric posterior HOX TF binding. We chose HOXC10 and HOXC13 because of their low and high ability to bind inaccessible chromatin, respectively. We found that the homeodomain and C-terminus of the protein control HOX TF ability to bind to inaccessible chromatin (Reviewer Fig. 1) (New Manuscript Fig. 8C-E). This alternative will force the transcription factor and developmental biology fields to look at homeodomains beyond DNA-sequence preference, with the additional pioneer dimension.

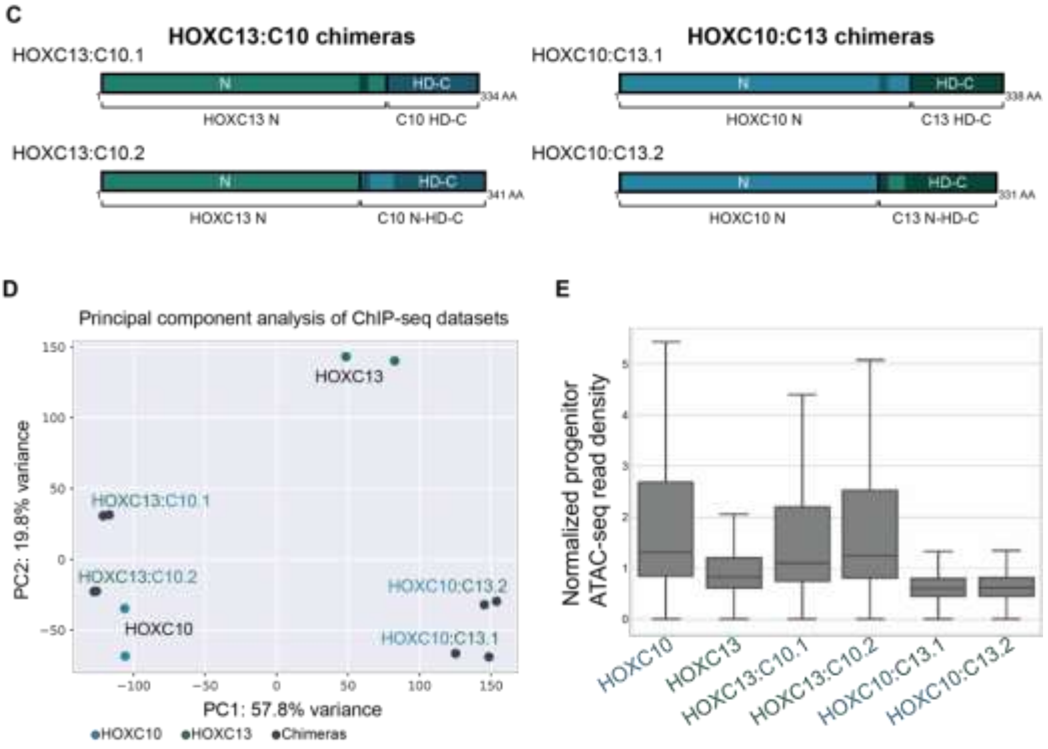

Reviewer Fig. 1 (New Manuscript Fig. 8C-E): (C) Schematic describing HOXC13:C10 and HOXC10:C13

© 2020. Published by The Company of Biologists under the terms of the Creative Commons Attribution License (https://creativecommons.org/licenses/by/4.0/).

chimeric HOX proteins. HD: Homeodomain. (D) Principal Component Analysis (PCA) of the ChIP-seq datasets reveals similarities in the binding patterns of HOX TFs. (E) The distribution of Day 2 progenitor ATAC-seq read density at the top 10,000 binding sites of the indicated HOX TF at Day 3. Data is ordered based on normalized read density (tags per million per site) and divided into quartiles. The box displays the central 50% (quartile 2 and quartile 3) of the data, while the top and bottom whiskers represent the top 25% and bottom 25% (the top and the bottom quartiles) of the data, respectively.

Reviewer 3 Comments for the Author:
The authors are experts in the field of HOX regulation and have produced seminal work in the field. The differentiation of mouse embryonic stem cells to MNs by H. Wichterle had been a key advancement. However, it is clear that using this protocol the MNs acquire mostly hindbrain and/or anterior spinal cord identity (up to Hox5). Thus the main concern is that the authors study Hox TF binding activity in the context of previously established in vitro cellular chromatin landscapes that correspond to hindbrain or cervical spinal cord and not more posterior identities. In order to extrapolate the results and have some valid conclusions that also apply to the embryo it is of critical importance that the in vitro environment recapitulates as much as possible the one found in vivo. Recent studies by Metzis et al, Cell, 2018 have shown that posteriorization of epiblast cells occurs before neural induction and that the chromatin landscape of posterior progenitor cells expressing Cdx2, is completely different to the one found in anterior neural progenitors. These recent findings by Metzis et al, are also in agreement with the findings published by Mazzoni et al, Nat Neuroscience, 2013. There the authors showed that remodelling of Hox chromatin in response to WNT/FGF signals induces the expression of Cdx2, which is important for the opening of the Hox5-9 cluster and emphasized the importance of using the correct patterning signals to induce chromatin remodelling early on.
Additionally, it has been shown by other groups that addition of WNT/FGF at an early time point of differentiation induces the formation of neuromesodermal progenitors (summarized in Henrique et al 2014), which are important for the generation of posterior spinal cord neurons of brachial, thoracic and lumbar identity (Gouti et al, 2014, Lippmann et al, 2015). Collectively, all these findings come in agreement with the distinct developmental origin of posterior spinal cord cells and nicely explain why it has not been possible to generate more posterior neurons using the Wichterle protocol. The authors will have at least to discuss this literature in their discussion and highlight the caveat in their study.

We agree with the Reviewer that the overexpression of caudal HOX TFs is done in an unnatural rostro-caudal chromatin environment, and not in neuromesodermal progenitors. We failed to discuss these issues in the manuscript and have now added a segment to the discussion describing the experimental limitations.

In the future it will be necessary to assess the chromatin landscape and binding of Hox TFs under conditions that allow the activation of the endogenous posterior Hox genes. This will provide conclusive evidence and give a final answer to the important question of how specific spinal cord identities are established in the spinal cord.

This study aimed to eliminate TF-extrinsic factors that could affect HOX binding to reveal TF-intrinsic preferences. Comparing rostral HOX binding in cervical motor neurons to caudal HOX binding in posterior neuromesodermal progenitors will result in challenging comparisons due to the multiple variable changes across experiments. Through gain-of-function experiments in chick and during *in vitro* differentiation, HOX overexpression in the “wrong” rostro-caudal motor position induces expression changes in register to their patterning activity. Thus, although in a somehow unnatural environment, we believe we can extract meaningful conclusions about HOX TF binding. After these reductionist studies, neuromesodermal progenitors are undoubtedly the way forward to understand how HOX genes pattern caudal structures. We added this limitation to the discussion.

Specific points
-How efficient is the induction of iHox genes using the tet-on system. What is the percentage of cells that express the specific Hox gene?

The tet-on system (PMID: 22039605) has been used in several previous publications which showed robust expression of tagged HOX, OLIG2, NEUROG2, and NICD proteins after Doxycycline treatment

© 2020. Published by The Company of Biologists under the terms of the Creative Commons Attribution License (https://creativecommons.org/licenses/by/4.0/).

in the same differentiation system (PMID: 22081127, PMID: 20826310, PMID: 27425621).
All inducible *Hox* are inserted in the same landing pad at HPRT. Thus, to obtain a robust measurement of the system's induction efficiency and strength, we quantified the percentage of cells which express inducible GFP from the same landing pad. We differentiated and induced GFP as done for *Hox* expression and quantified GFP at the same day we perform ChIPs (Reviewer Fig. 3) (new Manuscript Fig. S1B).

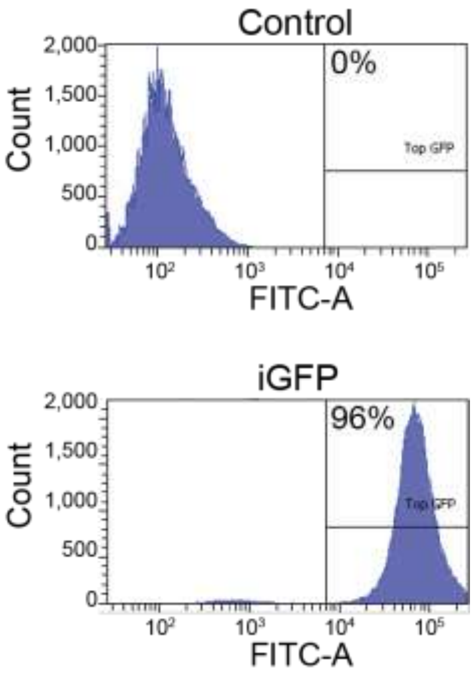

Reviewer Fig. 3 (new Manuscript Fig. S1B): FACS analysis of a No Dox Control and inducible GFP (iGFP) line on Day 3 of RA/SAG differentiation (iGFP line treated with Dox for 24h prior to FACS). Percentage of GFP+ cells indicated on the graph.

-The authors show that Hoxc9 overexpression represses more anterior Hox genes (Hox1-5). What is the situation with the other Hox genes (Hoxc6, Hoxc8 and Hoxc10)? How the authors explain that they don't repress more anterior Hox genes?

As the Reviewer suggests, HOXC9 represses rostral *Hox* genes *in vivo* and *in vitro*. However, brachial and cervical *Hox* genes are co-expressed in certain combinations *in vivo*. In addition, the late motor neuron pool specification where there is some cross-repression is not reproduced during *in vitro* differentiation (already discussed). Thus, we were not expecting a robust rostral *Hox* gene repression by *Hoxc6* and *Hoxc8*.

As far as HOXC10 repressing cervical *Hox* genes, we cannot find evidence for either scenario in previous work. However, *Hoxc9* expression does repress rostral *Hox* genes in these conditions. Thus, we expect HOXC10 to do so in this system if it would have the intrinsic ability to do so. We therefore have no evidence to conclude HOXC10 has the intrinsic ability to globally repress rostral *Hox* gene expression.

-In the iHoxc10 line the expression of Hoxa6 and Hoxc6 is very high. This is unexpected and the question arises if this is physiologically relevant. Is this caused by the high levels of transgene expression? Can the authors check in vivo using chick in ovo electroporation if this will be the case?

While HOXC10 induces some expression of other limb-level *Hox* genes, the relative fold change is misleading in Figure 1D. There are extremely few *Hox6* reads at Day 2 but some at the motor neuron stage, inflating the relative ratio (Reviewer Fig. 4) (modified Manuscript Fig. 1D). We included a table with FPKM values in Fig.
S2C, which hopefully clarifies this issue (Reviewer Table 2) (modified Manuscript Fig. S2C).

© 2020. Published by The Company of Biologists under the terms of the Creative Commons Attribution License (https://creativecommons.org/licenses/by/4.0/).

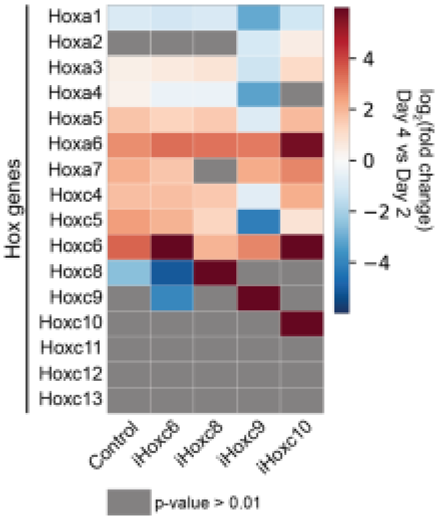

Reviewer Fig. 4 (modified Manuscript Fig. 1D): RNA-seq heatmap showing the expression of *Hox* genes in the No Dox control, iHoxc6, iHoxc8, iHoxc9 and iHoxc10 relative to progenitors (Day 2).

Fragments per Kilobase Per Million (FPKM), Gene Lengths calculated using Rsubread

Gene	pMN_r1	pMN_r2	pMN_r3	noDox_r1	noDox_r2	noDox_r3	iHoxc6_r1	iHoxc6_r2	iHoxc6_r3	iHoxc8_r1	iHoxc8_r2	iHoxc8_r3	iHoxc9_r1	iHoxc9_r2	iHoxc9_r3	iHoxc10_r1	iHoxc10_r2	iHoxc10_r3
Hoxc5	15.89	25.41	26.02	161.40	126.28	128.51	98.21	138.92	80.36	53.01	71.50	54.40	1.41	1.87	0.81	48.66	36.91	42.81
Hoxc6	0.11	0.17	0.24	3.28	1.58	1.16	324.27	303.89	314.14	0.97	0.46	0.71	1.21	1.44	1.32	31.21	31.58	27.34
Hoxc8	0.06	0.17	0.03	0.01	0.00	0.03	0.00	0.00	0.00	165.47	171.09	148.19	0.07	0.01	0.05	0.15	0.12	0.07
Hoxc9	0.06	0.08	0.02	0.02	0.02	0.02	0.00	0.00	0.00	0.02	0.02	0.00	128.21	134.77	121.64	0.04	0.02	0.05
Hoxc10	0.00	0.00	0.00	0.00	0.00	0.00	0.00	0.00	0.00	0.00	0.00	0.00	0.00	0.18	0.02	227.18	226.11	228.20

UTR	pMN_r1	pMN_r2	pMN_r3	noDox_r1	noDox_r2	noDox_r3	iHoxc6_r1	iHoxc6_r2	iHoxc6_r3	iHoxc8_r1	iHoxc8_r2	iHoxc8_r3	iHoxc9_r1	iHoxc9_r2	iHoxc9_r3	iHoxc10_r1	iHoxc10_r2	iHoxc10_r3
Hoxc5 UTR	10.37	10.59	14.75	72.43	61.86	69.76	50.06	49.06	47.23	27.94	34.32	25.02	0.73	0.76	0.50	20.26	22.09	22.48
Hoxc6 UTR	0.12	0.27	0.26	2.26	0.60	0.57	1.39	1.17	1.40	0.90	0.38	0.70	1.32	1.52	1.16	27.72	30.53	25.23
Hoxc8 UTR	0.09	0.09	0.03	0.07	0.00	0.00	0.00	0.00	0.00	14.50	13.73	14.73	0.12	0.13	0.11	0.03	0.13	0.06
Hoxc9 UTR	0.02	0.06	0.02	0.02	0.02	0.02	0.00	0.00	0.00	0.02	0.00	0.00	0.02	0.02	0.05	0.02	0.04	0.05
Hoxc10 UTR	0.00	0.00	0.00	0.00	0.00	0.00	0.00	0.00	0.00	0.00	0.00	0.00	0.00	0.00	0.00	0.04	0.04	0.09

Reviewer Table 2 (modified Manuscript Fig. S2C): Normalized (FPKM) read counts for *Hox* genes and their UTRs in the indicated conditions (in 3 biological replicates) (pMN: progenitors (Day2)).

As the Reviewer, we also found it interesting that HOXC10 induces *Hox6* expression. Although HOXC10 binds to other rostral *Hox* regions, it only induces *Hox* genes also associated with limb-level fates. Thus, we speculate *Hoxc10* induction of the LMC program might result in a previously unnoticed weak feedback.
Alternatively, it is a limitation of the ESC-to-MN differentiation system we use. We added this caveat to our discussion.
The Reviewer proposes an interesting experiment in chick to confirm *Hox6* induction by HOXC10. A few years ago, we sadly dismantled our chick set up. Due to the restricted lab access because of the pandemic, it will take us months to find all the instruments and perform the experiment. Because it is not the central point of the paper, we believe it is not necessary to support the conclusions and thus something we can leave for future studies.

-Is the expression of a specific HOX TF sufficient to install the specific positional identity? Are the endogenous genes activated?
To address this question, we mapped RNA-seq reads from Day 2 progenitors, Day 4 No Dox controls and Day 4 iHoxc6, iHoxc8, iHoxc9, and iHoxc10 onto the UTRs of endogenous *Hoxc5*-*Hoxc10* genes because *Hox* transgenes do not contain UTRs. Reads mapping to endogenous gene UTRs suggest that the endogenous *Hoxc8* gene seems to be induced in iHoxc8 neurons (Reviewer Table 2) (modified Manuscript Fig. S2C).

© 2020. Published by The Company of Biologists under the terms of the Creative Commons Attribution License (https://creativecommons.org/licenses/by/4.0/).

Fragments per Kilobase Per Million (FPKM), Gene Lengths calculated using Rsubread

Gene	pMN_r1	pMN_r2	pMN_r3	noDox_r1	noDox_r2	noDox_r3	iHoxc6_r1	iHoxc6_r2	iHoxc6_r3	iHoxc8_r1	iHoxc8_r2	iHoxc8_r3	iHoxc9_r1	iHoxc9_r2	iHoxc9_r3	iHoxc10_r1	iHoxc10_r2	iHoxc10_r3
Hoxc5	15.89	25.41	26.02	161.40	126.28	128.51	98.21	138.92	80.36	53.01	71.50	54.40	1.41	1.87	0.81	48.66	36.91	42.81
Hoxc6	0.11	0.17	0.24	3.28	1.58	1.16	324.27	303.89	314.14	0.97	0.46	0.71	1.21	1.44	1.32	31.21	31.58	27.34
Hoxc8	0.06	0.17	0.03	0.01	0.00	0.03	0.00	0.00	0.00	165.47	171.09	148.19	0.07	0.01	0.05	0.15	0.12	0.07
Hoxc9	0.06	0.08	0.02	0.02	0.02	0.02	0.00	0.00	0.00	0.02	0.02	0.00	128.21	134.77	121.64	0.04	0.02	0.05
Hoxc10	0.00	0.00	0.00	0.00	0.00	0.00	0.00	0.00	0.00	0.00	0.00	0.00	0.00	0.18	0.02	227.18	226.11	228.20

UTR	pMN_r1	pMN_r2	pMN_r3	noDox_r1	noDox_r2	noDox_r3	iHoxc6_r1	iHoxc6_r2	iHoxc6_r3	iHoxc8_r1	iHoxc8_r2	iHoxc8_r3	iHoxc9_r1	iHoxc9_r2	iHoxc9_r3	iHoxc10_r1	iHoxc10_r2	iHoxc10_r3
Hoxc5 UTR	10.37	10.59	14.75	72.43	61.86	69.76	50.06	49.06	47.23	27.94	34.32	25.02	0.73	0.76	0.50	20.26	22.09	22.48
Hoxc6 UTR	0.12	0.27	0.26	2.26	0.60	0.57	1.39	1.17	1.40	0.90	0.38	0.70	1.32	1.52	1.16	27.72	30.53	25.23
Hoxc8 UTR	0.09	0.09	0.03	0.07	0.00	0.00	0.00	0.00	0.00	14.50	13.73	14.73	0.12	0.13	0.11	0.03	0.13	0.06
Hoxc9 UTR	0.02	0.06	0.02	0.02	0.02	0.02	0.00	0.00	0.00	0.02	0.00	0.00	0.02	0.02	0.05	0.02	0.04	0.05
Hoxc10 UTR	0.00	0.00	0.00	0.00	0.00	0.00	0.00	0.00	0.00	0.00	0.00	0.00	0.00	0.00	0.00	0.04	0.04	0.09

Reviewer Table 2 (modified Manuscript Fig. S2C): Normalized (FPKM) read counts for *Hox* genes and their UTRs in the indicated conditions (in 3 biological replicates) (pMN: progenitors (Day2)).

-It has been previously shown by Dasen et al, Nature, 2003 that over expression of Hoxc9 in the brachial neural tube of chick embryos is sufficient to repress Hoxc6 expression and induce autonomic MN identity. Thus, one would expect that PGC identity is induced in the iHoxc9 line. Have the authors looked into the expression of Bmp5, nNOS and pSMAD associated with PGCs?

Like the Reviewer, we thought the same. In our original description characterizing *Hoxc9* thoracic fate induction by global repression, we looked hard for PGC generation (PMID: 20826310). We confirmed *Hoxc9* gain-of-function repressed all rostral *Hox* and the LMC program. However, iHoxc9 does not induce Bmp5 or nNos (Reviewer Fig. 5). Because PGCs express low *Foxp1* during development, we think the gain-of-function experiment in this system struggles to achieve the balance and correct *Foxp1* expression levels needed for PGC identity at the time points we investigated. It was, and still is, frustrating because what can now be called "spinal organoids" were producing some PGCs (PMID: 20804971).
To our knowledge, no PGCs were generated from mouse and human pluripotent stem cells by the "Wichterle protocol." Echoing other Reviewer 3 suggestions and comments, thoracic identity is, for sure better suited for neuromesodermal protocols. We added this caveat to the limitations of the approach in the discussion.

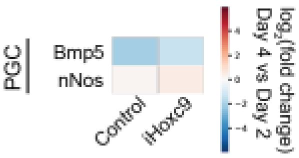

Reviewer Fig. 5: RNA-seq heatmap showing the expression of representative PGC marker genes in the No Dox control and iHoxc9 relative to progenitors (Day 2).

<u>Second decision letter</u>

MS ID#: DEVELOP/2020/194761

MS TITLE: Differential abilities to engage inaccessible chromatin diversify vertebrate HOX binding patterns

AUTHORS: Milica Bulajic, Divyanshi Srivastava, Jeremy Dasen, Hynek Wichterle, Shaun Mahony, and Esteban Mazzoni
ARTICLE TYPE: Research Article

I am happy to tell you that your manuscript has been accepted for publication in Development, pending our standard ethics checks.

© 2020. Published by The Company of Biologists under the terms of the Creative Commons Attribution License (https://creativecommons.org/licenses/by/4.0/).

Reviewer 1

Advance summary and potential significance to field

I am happy with the detailed revisions provided by the authors and fully support publication.

Comments for the author

I am happy with the detailed revisions provided by the authors and fully support publication.

Reviewer 2

Advance summary and potential significance to field

The manuscript by Bulajic et al. reports on the analysis of HoxC differential binding during in vitro motor neuron differentiation. Through the comparative analysis of HoxC6, 8, 9, 10 and 13, the authors convincingly show that the differential binding of the HoxC transcription factors cannot be simply explained by differences in DNA sequence preference. Interestingly, they found that these HoxC TFs have distinct abilities to bind to inaccessible chromatin. Moreover, they reveal that following induction of individual HoxC TF, HoxC9 and HoxC13 have a marked preference for inaccessible chromatin compared to the other HoxC TFs tested. Finally, they show that HoxC9 and HoxC13 induction results in increase chromatin accessibility at their targets.

While previous evidence pointed to Hox TFs having the ability to trigger chromatin accessibility both in flies and mice, the finding making the present work of particular significance is that this property varies among the HoxC TFs. This difference is observed not only for the different HoxC TFs but also among members of the same paralogous group (namely paralogous group 9), which is particularly interesting knowing the partial functional overlap often observed between Hox paralogs even when these paralogs have highly similar expression patterns.

In summary, this work provides important new information regarding Hox functional properties and their associated developmental processes, which should be of interest to the developmental biology community.

Comments for the author

The authors have appropriately answered all comments made by the reviewers and I fully support publication of the revised version of their manuscript.

Reviewer 3

Advance summary and potential significance to field

The authors have addressed my main concerns and I am happy to support the publication of the manuscript in Development. I wish to the authors every success in their future studies.

Comments for the author

--

© 2020. Published by The Company of Biologists under the terms of the Creative Commons Attribution License (https://creativecommons.org/licenses/by/4.0/).
